# SO_2_ Transfer Enabled by an Easy‐to‐Handle Ionic Liquid

**DOI:** 10.1002/chem.71160

**Published:** 2026-05-29

**Authors:** Johanna S. Sturm, Letizia Lanfredi, Severin Erbertseder, Marius Balizs, Patrick Voßnacker, Simon Steinhauer, Merlin Kleoff, Anja Wiesner, Niklas Limberg, Patrick Pröhm, Franz‐Lucas Haut, Sebastian Riedel

**Affiliations:** ^1^ Institut für Chemie und Biochemie Anorganische Chemie Freie Universität Berlin Berlin Germany; ^2^ Institut für Chemie und Biochemie Organische Chemie Freie Universität Berlin Berlin Germany

**Keywords:** green chemistry, ionic liquid, reagent design, synthetic methods, sulfur dioxide

## Abstract

Herein, the ionic liquid [NEt_3_Me][Cl(SO_2_)*_n_*] is reported as a reversible sulfur dioxide storage medium and transfer reagent for organic synthesis. Vapor pressure measurements revealed that the ionic liquid can store up to 3.66 equivalents of SO_2_ at room temperature, which can be released in a controlled manner upon heating or depressurization. The physicochemical properties were investigated, and long‐term stability was demonstrated, as no decomposition was observed for more than six months. Based on its solid‐state structure, the bonding analysis indicated a covalent sulfur‐chlorine interaction, which is consistent with conducted Raman studies and quantum chemical calculations. Atom economy and cost estimates are compared with those of commonly used SO_2_ surrogates, highlighting the developed transfer reagent as an inexpensive and (atom‐)economic alternative for laboratory‐scale applications. The synthetic utility of [NEt_3_Me][Cl(SO_2_)*_n_*] is further demonstrated, enabling the synthesis of valuable organosulfur compounds such as 3‐sulfolenes, sulfonamides, sulfones, and sulfonyl fluorides in good to excellent yields. Importantly, the incorporation of SO_2_ is scalable, rendering [NEt_3_Me][Cl(SO_2_)*_n_*] as a safe and user‐friendly SO_2_ transfer reagent for synthetic applications.

## Graphical abstract

**Best of both worlds**: The ionic liquid [NEt_3_Me][Cl(SO_2_)*_n_*] unites the atom economy and low cost of sulfur dioxide with the safety and applicability of common surrogates, streamlining SO_2_ transfer to access to 3‐sulfolenes, sulfonamides, and SuFEx reagents.

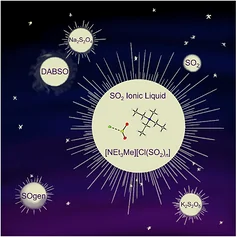

## 1 Introduction

Gaseous reagents are indispensable in synthetic chemistry, enabling efficient incorporation of functional groups and playing a key role in the production of industrially relevant feedstock chemicals [1]. However, the handling and storage of these usually highly reactive reagents display significant safety concerns in both laboratory‐scale and industrial settings [2]. Consequently, the development of secure and practical storage strategies for such gases continues to attract considerable research interest. One promising approach is to use ionic liquids as storage media [3]. Ionic liquids (ILs)—defined as salts with melting points below 100°C—offer access to green and tailor‐made solvents and reagents. Their physicochemical properties, including vapor pressure, thermal stability, electric conductivity, and viscosity, can be systematically adjusted through judicious selection of the cationic and anionic components. As demonstrated previously, highly reactive gases such as chlorine (Cl_2_) and hydrogen chloride (HCl) can be effectively stored using triethylmethylammonium chloride, [NEt_3_Me]Cl, forming the corresponding ILs [NEt_3_Me][Cl(Cl_2_)*_n_*] and [NEt_3_Me][Cl(HCl)*_n_*], respectively [4, 5, 6]. These storage media exhibit low vapor pressure, low viscosity, and long‐term stability, enabling controlled gas release by heat or reduced pressure. This allows on‐demand use as transfer reagents for chlorination or hydrochlorination reactions, respectively, while providing user‐friendly alternatives to otherwise highly reactive and difficult‐to‐handle gaseous reagents [7, 8].

Building on our recent progress in providing highly reactive reagents, such as Cl_2_ and HCl, as ILs for synthetic transformations, we sought to extend the concept of reversible gas storage to sulfur dioxide (SO_2_), an essential reagent with broad applicability in synthetic chemistry. Sulfur‐containing molecules are essential across materials science [9], agrochemistry [10], and medicinal chemistry, with sulfur being one of the most prevalent elements in approved pharmaceuticals [11, 12]. Consequently, the development of synthetic methods to incorporate SO_2_ into organic compounds has gained significant attention to construct value‐added compounds through photo‐ and electrochemical methods [13], radical and multicomponent reactions [14, 15, 16], or transition metal‐catalyzed transformations [14, 15, 17]. More recently, Sulfur fluoride exchange (SuFEx) chemistry has emerged as a powerful technology, as sulfonyl fluorides (RSO_2_F) effectively modulate the reactivity and the properties of bioactive molecules [18, 19, 20]. This enables the synthesis of diverse functionalized molecules under physiologically relevant conditions, with applications in drug discovery and polymer science [21]. Despite the advances realized in sulfonyl fluoride synthesis, some of the most straightforward approaches rely on the utilization of SO_2_. Gaseous SO_2_ is inexpensive, abundant, reacts under mild conditions, and an excess of SO_2_ gas can easily be removed. However, due to its toxicity, corrosivity, and high vapor pressure at room temperature (3.5 bar at 20°C) [22], handling SO_2_ gas is inherently hazardous and requires pressure‐resistant equipment as well as stringent safety precautions.

**FIGURE 1:**
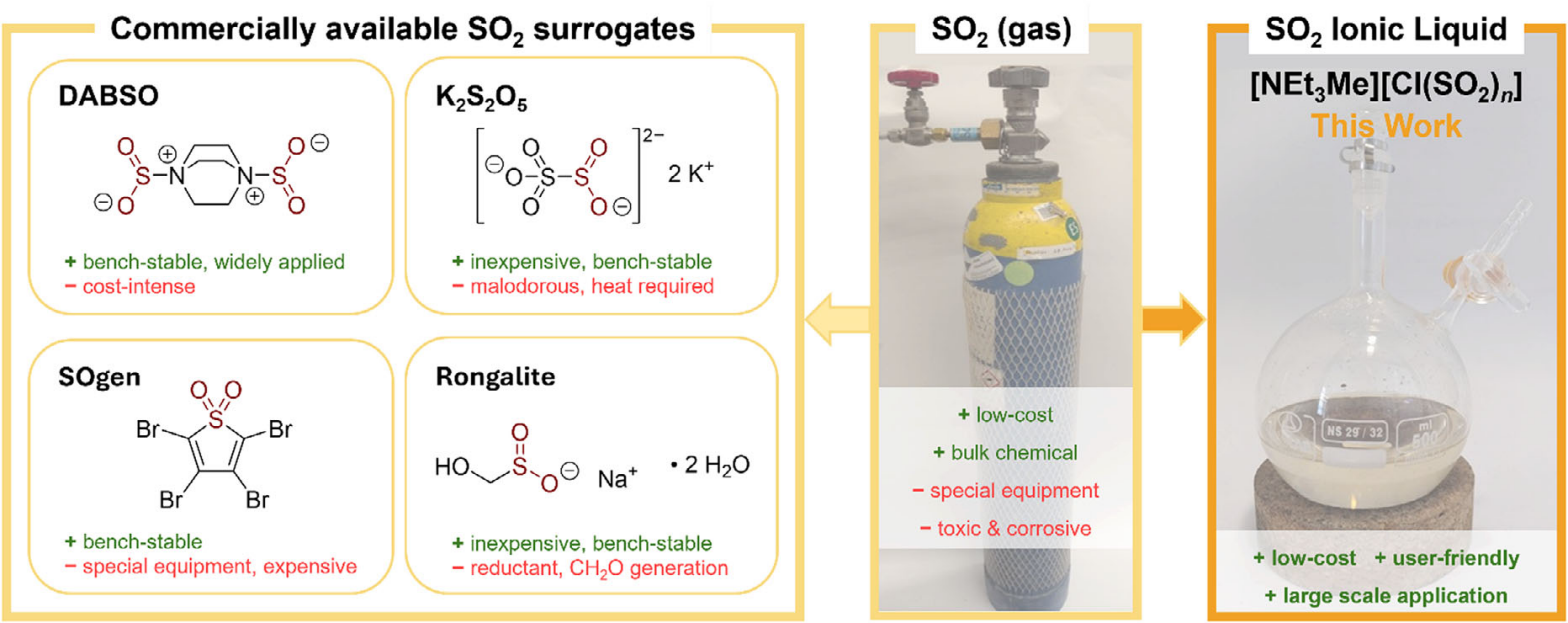
Overview of selected SO_2_ surrogates (*left*), SO_2_ gas as (atom)‐economic but hard‐to‐handle reagent (*center*), and our developed ionic liquid [NEt_3_Me][Cl(SO_2_)*_n_*] (*right*, **this work**) as a user‐friendly and inexpensive alternative for SO_2_ transfer.

Due to practical limitations associated with handling SO_2_ gas on a laboratory scale, user‐friendly solutions of SO_2_ in organic solvents such as MeCN, CH_2_Cl_2_, and THF are commercially available, albeit at high cost [23]. On the other hand, convenient and stable SO_2_ surrogates have been developed and applied to various synthetic transformations (Figure 1) [14, 15]. The most prominent example is the bis(sulfur dioxide) adduct of 1,4‐diazabicyclo[2.2.2]octan (DABCO), known as DABSO—a bench‐stable solid with no vapor pressure that has been widely applied for the synthesis of sulfur‐containing scaffolds [24, 25]. Unfortunately, DABSO is a relatively expensive reagent, and the presence of the amine base DABCO could lead to undesired side reactions [14, 26]. Another organic surrogate is tetrabromothiophene *S,S*‐dioxide (SOgen), relying on the release of SO_2_ through a cascade reaction composed of a [4+2] cycloaddition with a dienophile followed by a retro‐[4+1] cycloaddition reaction [26]. Importantly, the application of SOgen requires specialized equipment for ex situ SO_2_ generation (two‐chamber systems) as well as the addition of a styrene as dienophile. In addition to its low atom economy and high costs, the reaction mechanism does not allow reuse of the surrogate after SO_2_ transfer [14]. More affordable alternatives are based on inorganic sulfites, such as K_2_S_2_O_5_ and Na_2_S_2_O_4_, and typically require heat or acidic reaction media, as well as the use of radical intermediates or precious transition metal catalysts to incorporate SO_2_ [27]. Although interest in these SO_2_ surrogates is increasing, the mechanism and reactivity of metabisulfites are not yet fully understood and dithionate can act as a reductant [28, 29, 30]. In addition, Na_2_SO_3_ could be used for the controlled release of SO_2_ when exposed to sulfuric acid (H_2_SO_4_), but a special reactor is required and the gas extrusion has been found to be an overall slow process [31, 32]. Another inexpensive alternative is sodium hydroxymethanesulfinate dihydrate (Rongalite), which has also been utilized as a powerful reducing agent, yet producing formaldehyde as a potentially problematic byproduct [33, 34, 35, 36].

Given the challenges associated with using SO_2_ gas and established SO_2_ surrogates, exploring user‐friendly alternatives is highly desirable. Ideally, such a transfer reagent should not produce reactive byproducts, require specialized equipment or strict safety precautions during handling, and be inexpensive, scalable, atom‐economical, and reusable. Inspired by our ongoing research program on chlorination reactions mediated by ILs [7, 8] and the preliminary work of Kumar et al. [37], we envisioned quaternary ammonium chlorides [NR_4_]Cl as promising SO_2_ transfer reagents. In particular, we aimed to employ [NEt_3_Me]Cl as an inexpensive and readily available gas storage medium that enables the controlled handling and release of SO_2_, generating ammonium chloride as the sole, rather unreactive byproduct, which can be easily separated from the reaction mixture for reuse.

## 2 Results and Discussion

At the outset of this work, [NEt_3_Me]Cl was reacted with gaseous SO_2_. This reaction results in an immediate exothermic reaction accompanied by a phase change of the solid ammonium salt into a low‐viscosity colorless liquid (Figure 1, right). During SO_2_ loading, the reaction mixture pressure decreases, allowing the gradual addition of SO_2_ until equilibrium is reached and the vapor pressure remains constant. The ionic liquid was analyzed by NMR and Raman spectroscopy and identified as the corresponding ammonium chlorosulfite [NEt_3_Me][Cl(SO_2_)*_n_*] (*n * =  1−3.66). The vapor pressure increases proportionally to the loading of the IL.

### 2.1 Vapor Pressure

**FIGURE 2:**
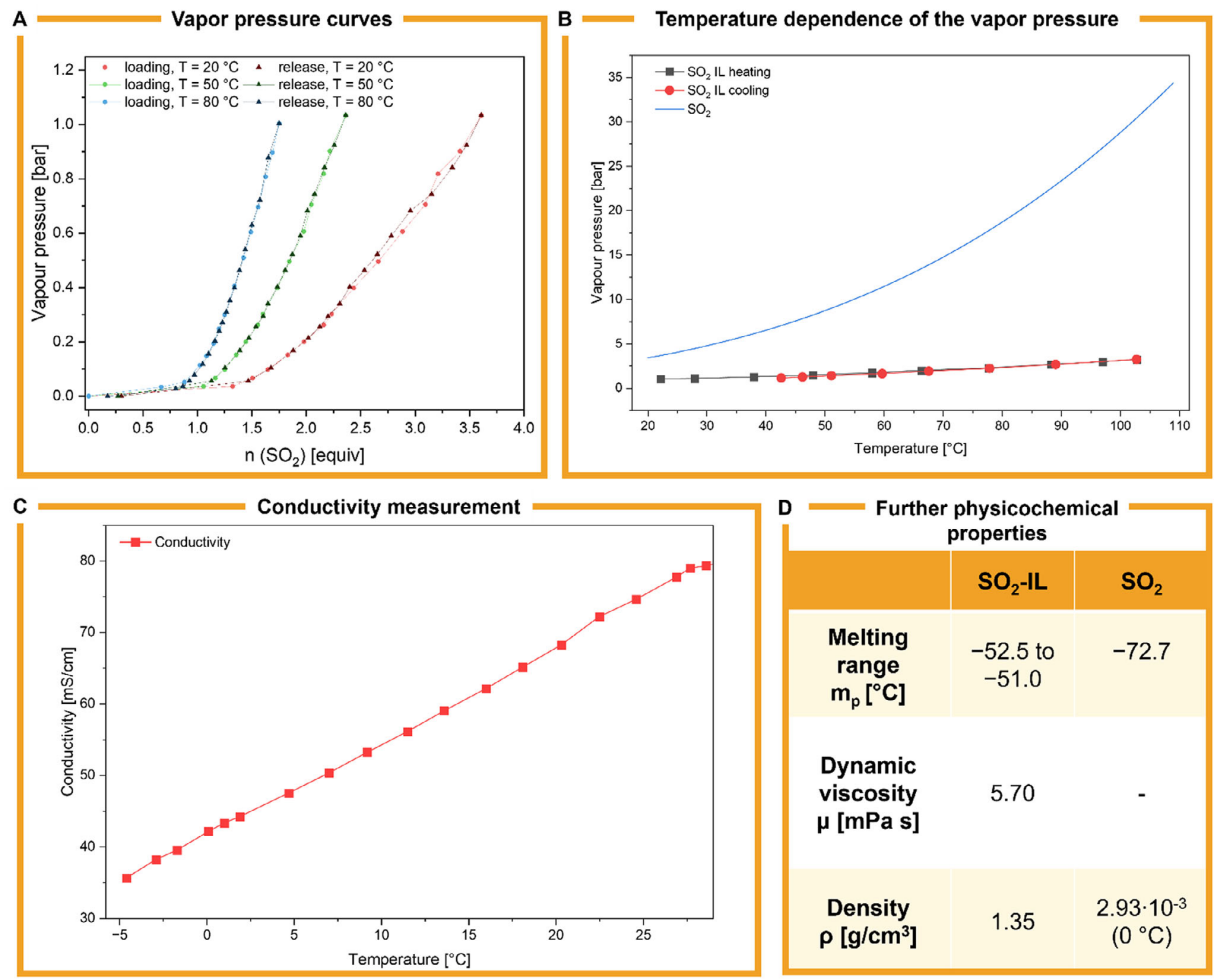
(A). Vapor pressure curves of [NEt_3_Me][Cl(SO_2_)*_n_*] at 20°C, 50°C, and 80°C. (B). Temperature dependence of the vapor pressure of [NEt_3_Me][Cl(SO_2_)_3.66_] in comparison to gaseous SO_2_ [22]. (C). Conductivity measurement of [NEt_3_Me][Cl(SO_2_)_3.66_]. (D). Summary of further physicochemical properties of [NEt_3_Me][Cl(SO_2_)_3.66_] [1].

Depending on the vapor pressure and temperature of the IL, different SO_2_ loadings can be achieved. To investigate this, vapor pressure curves were recorded at 20°C, 50°C, and 80°C (Figure 2A). SO_2_ was added stepwise and, after the equilibrium vapor pressure had been established, its amount was determined gravimetrically. The loading process was performed up to a vapor pressure of 1 bar. Then, SO_2_ was removed under reduced pressure and the loading was determined by the same procedure. The alignment of the loading and release curves indicates that the system is in equilibrium.

The vapor pressure increases with temperature as well as with the SO_2_ loading. The highest IL loading at 1 bar and 20°C is 3.61 equiv. or 1.53 g SO_2_ per 1 g ammonium salt, while it is 2.36 equiv. (1.00 g/g) at 50°C and 1.75 equiv. (0.74 g/g) at 80°C. Accordingly, the addition of SO_2_ equivalents at 20°C leads to slower increase in vapor pressure, than at 50 or 80°C. Consequently, SO_2_ can easily be released by increasing the ILs temperature at constant gas pressure. This has been confirmed by cyclic experiments, in which the IL was heated from 20°C to 80°C at 1 atm, freeing 1.7 equiv. SO_2_ (Figure S6) and demonstrating the reversibility of the storage process. To validate the storage capacities, an excess of SO_2_ was condensed onto the ammonium salt. Upon warming to 20°C, the excess SO_2_ was allowed to evaporate until a pressure of 1 atm was established. The IL was found to store up to 3.66 equiv. of SO_2_, which is in good agreement with the results of the vapor pressure experiment. Compared to the mol/mol ratio of the bichloride [NEt_3_Me][Cl(HCl)_2.53_] [4] and trichloride [NEt_3_Me][Cl(Cl_2_)_1.68_] [6], this is an even larger amount of gas that can be stored within the storage medium. The temperature dependence of the vapor pressure of the IL was determined by heating [NEt_3_Me][Cl(SO_2_)*_n_*] in a pressure‐safe container (Figure 2B). Within the temperature range from 20 to 90°C, the IL shows a rise in pressure from 1.0 to 2.6 bar, while that of gaseous SO_2_ rises from 3.4 to 23.4 bar, underlining the enormous safety advantage of the SO_2_‐IL [22]. This small increase in vapor pressure with temperature is a significant safety advantage compared to gaseous SO_2_.

### 2.2 Physicochemical Properties

To further characterize this IL and evaluate its applicability, physicochemical properties were investigated (Figure 2D). The melting range was measured to be −52.5°C to −51.0°C, highlighting its potential use in reactions at low temperatures while remaining liquid. With a dynamic viscosity of 5.70 mPa·s at 20°C, [NEt_3_Me][Cl(SO_2_)*_n_*] is belong the ILs with lowest viscosity [38, 39]. This is a significant advantage, especially in application, since it can easily be transferred with a syringe. The density of the IL was determined to be 1.35 g/cm^3^.

The long‐term stability of [NEt_3_Me][Cl(SO_2_)*_n_*] was investigated by ^1^H NMR studies. Therefore, samples of the IL were stored under the following conditions: a) daylight exposure, b) light exclusion, and c) 50°C and light exclusion (Figures S13–S15). No decomposition was observed over a period of ten months, making [NEt_3_Me][Cl(SO_2_)*_n_*] a stable storage system and SO_2_ surrogate, even at elevated temperatures.

Since sulfur‐containing additives hold many advantages in Lithium‐ion batteries, the electrochemical properties of the SO_2_ IL were evaluated [40, 41]. Temperature‐dependent conductivity measurements showcased a linear increase in conductivity with increasing temperature (Figure 2C). Above 28°C, degassing of the IL was observed, resulting in a stagnation in conductivity and making reliable measurement difficult. Compared to other ILs, [NEt_3_Me][Cl(SO_2_)*_n_*] exhibits a comparably high conductivity, which can be explained by its small ions and their high mobility [41, 42, 43].

### 2.3 Bond Analysis

**FIGURE 3:**
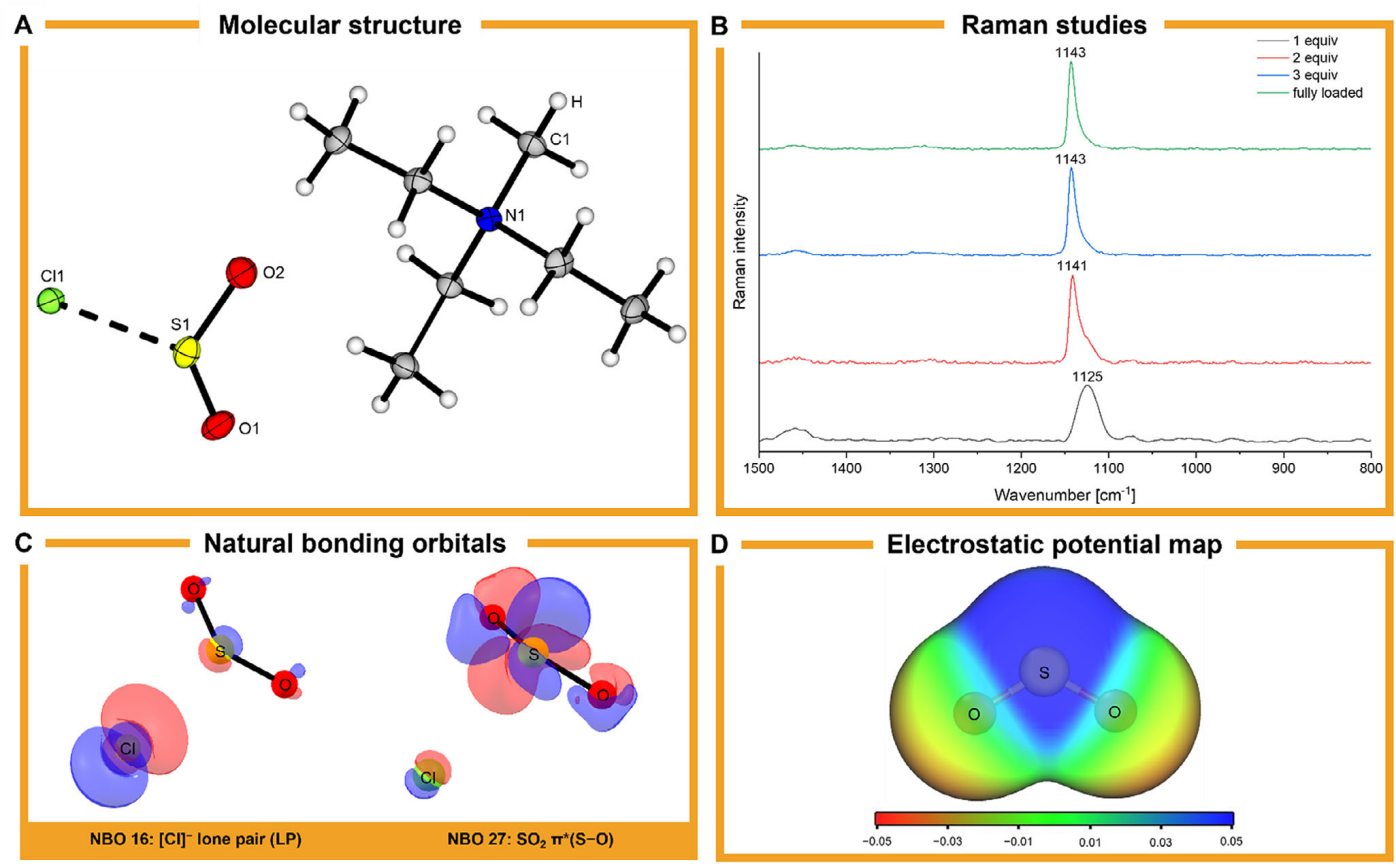
(A). Molecular structure of [NEt_3_Me][Cl(SO_2_)] in the solid state, obtained from a solution in CH_2_Cl_2_. Displacement ellipsoids are set at 50% probability. Obtained bond lengths [pm]: S1−O1 143.8(1), S1−O2 144.4(1), Cl1−S1 256.2(1), bond angle O−S−O 114.5(1)°. (B). Raman spectra of [NEt_3_Me][Cl(SO_2_)] (grey), [NEt_3_Me][Cl(SO_2_)_2_] (red), [NEt_3_Me][Cl(SO_2_)_3_] (blue), and [NEt_3_Me][Cl(SO_2_)_3.66_] (green). (C). Plotted natural bonding orbitals for the [Cl(SO_2_)]^−^ anion. Red and blue isosurfaces represent positive and negative orbital lobes (Isovalue = 0.4 a.u.). The interacting orbitals are the lone pair of chloride (NBO 16) and the π*‐Orbital of S−O (NBO 27). (D). Electrostatic potential around sulfur dioxide, SO_2_, mapped on an isosurface (0.0035 a.u.) of its electron density, ranging from −0.05 a.u (red) to 0.05 a.u (blue). Calculated at B3LYP‐D3BJ/def2‐QZVPP level in gas phase.

The molecular structure of [NEt_3_Me][Cl(SO_2_)] was determined by x‐ray diffraction of single crystals obtained from a saturated solution in CH_2_Cl_2_ at −80°C (Figure 3A). The compound crystallizes in the monoclinic space group *P*2_1_/*n*. The structure shows a slightly distorted tetrahedral arrangement of the alkyl groups around the N atom of the [NEt_3_Me]^+^ ion. The SO_2_ moiety is coordinated by the chloride ion at a distance of 256.2(1) pm. The S─O bonds are 143.8(1) and 144.4(1) pm and therefore slightly elongated compared to the 143.1(2) pm bond length of isolated molecular SO_2_ [44, 45]. This indicates that the interaction between sulfur and chloride slightly weakens the S─O bonds. The O─S─O bond angle in the coordinated SO_2_ is 114.5(1)°, which is smaller than the 118.5(10)° bond angle in isolated SO_2_ [44, 45]. These elongated bond lengths and the decreased bond angle are consistent with the literature‐known [37] findings reported for [NEt_4_][Br(SO_2_)] [46, 47].^1^ Although, all attempts to obtain crystals of [NEt_3_Me][Cl(SO_2_)*_n_*] with more than one SO_2_ equivalent failed, this was achieved for the larger [NEt_4_]^+^, [PNP]^+^, and [PPh_4_]^+^ cations (Figure S1–S4).

To evaluate the bonding situation of larger [Cl(SO_2_)*_n_*]^−^ anions, the IL was analyzed using Raman spectroscopy at different loadings of SO_2_ (Figure 3B). A comparison of the symmetric sulfur‐oxygen stretching vibration shows a slight shift to lower wavenumbers compared to the corresponding vibration of isolated SO_2_ at 1143 cm^−1^. The strongest shift was observed in the single coordinated compound (*n*  =  1), with a band at 1125 cm^−1^. The double coordinated compound (*n*  =  2) exhibits a vibration at 1141 cm^−1^, while the triple coordinated (*n*  =  3) and fully loaded IL (*n*  =  3.66) matches the vibration of isolated SO_2_. Kumar et al. described a similar behavior of the symmetric SO stretching vibration for [NEt_4_][Cl(SO_2_)*_n_*] and [NEt_4_][Br(SO_2_)*_n_*], which they explained by electrostatic interactions between the halide ion and SO_2_ [37]. Furthermore, ^1^H NMR spectra of the IL at different loadings were measured according to the Spange approach [48]. As the loading increases, the signals are further low field shifted, indicating an increasing interaction between the cation and its environment (Figures S9 and S10) [48, 49]. The same trend was observed when dissolved in CD_2_Cl_2_ (Figures S11 and S12).

DFT calculations at the B3LYP/def2‐QZVPPD level were used to investigate the bonding situation further. The structures of an isolated [Cl(SO_2_)*_n_*]^−^ with n = 1, 2, 3, and 4 were optimized and show an increasing distance between Cl^−^ and SO_2_ as the number of SO_2_ molecules rises, ranging from 253.3 to 298.8 ppm. Additionally, the S─O symmetric stretching vibration shifts to higher wavenumbers as the bond length decreases, ranging from 1116 to 1163 cm^−1^. These results confirm the experimental findings.

Electrostatic potential maps (ESP) of SO_2_ (Figure 3D) and isolated [Cl(SO_2_)]^−^ (Figure S20) were used to gain insight into their electronic structures. SO_2_ has a more negative electrostatic potential at the oxygen atoms, and electron density is depleted around the sulfur atom. The remaining lone pair renders the out‐of‐plane side of the sulfur the most accessible for nucleophiles. In [Cl(SO_2_)*_n_*]^−^ the electrostatic potential of the sulfur atom is increased due to charge transfer.

Natural Bond Orbital (NBO) analysis was performed for [Cl(SO_2_)]^−^ (Figure 3C). The interacting orbitals were identified as the orbital that mainly consists of the lone pair on the chloride ion and the partially occupied π*‐orbital of the S─O bond. These orbitals form the basis of the charge transfer interaction of the chlorosulfite [50]. According to second‐order perturbation theory of the Fock matrix in the NBO basis, this interaction corresponds to an energy of 192 kJ/mol, indicating a strong donor‐acceptor interaction between chloride and the sulfur atom. Unlike typical halogen bonding, where the halide interacts with a σ*‐orbital, the charge transfer occurs between halide and π*‐orbital, resulting in a comparably small weakening of the S−O bond. This is reflected by the small shifts observed in the Raman bands.

Quantum Theory of Atoms in Molecules (QTAIM) analysis was performed for isolated SO_2_ and the [Cl(SO_2_)]^−^ complex (Figure S21) The corresponding bond critical points (BCPs) and their properties are summarized in Table S7. Due to molecular symmetry, two nearly identical BCPs are observed for SO_2_ with electron densities of 2.117 and 2.116 eA^−3^, an electron localization function (ELF) value of 0.29, and the quotient of potential energy density and Lagrangian kinetic energy density, |V|/G, with 1.6. Even though the Laplacian shows high positive values of 25.35 and 25.31 eA^−5^, the S─O bonds exhibit covalent bond character [51]. The ellipticity reveals anisotropic bonding, which is consistent with π bond character between S and O. For [Cl(SO_2_)]^−^, the S─O bonds exhibit reduced electron densities and lower Laplacian values, which, together with a decreased ellipticity, is in agreement with experimental findings of diminished π bond character and a slightly weakened S─O bond. The BCP between S and Cl shows an electron density of 0.386 eA^−3^, a Laplacian of 1.70 eA^−5^, an ELF value of 0.44, and a |V|/G ratio of 1.36. These values are consistent with the formation of a covalent bond with strong charge‐shift character between sulfur and chloride due to the many lone pairs on both atoms.

To further investigate the interaction energy in [Cl(SO_2_)*_n_*]^−^, the chloride ion affinity (CIA) was calculated with the trimethylsilylium cation as anchor point (Table S5). AlCl_3_ and BCl_3_ were included as computational references indicating good agreement with their experimental values. The CIA of SO_2_ was determined as 123.0 kJ/mol, which is between that of Cl_2_ (136.0 kJ/mol) and HCl (115.2 kJ/mol). This finding reflects the Lewis acidic character of SO_2_, arising from the electron‐withdrawing effect of the oxygen atoms making the sulfur electron‐deficient and indicates an energetically favored adduct formation of SO_2_ and Cl^−^. While additional factors must be considered, CIAs can be used as an initial hint to estimate if an adduct i.e. an IL, might be formed.

### 2.4 Atom Economy and Cost Estimation

To compare the large‐scale applicability of [NEt_3_Me][Cl(SO_2_)*_n_*] with DABSO, SOgen, and sulfite salts, we calculated the atom economy and the costs of the reagents (Table 1) [52]. These calculations show that the IL has the highest atom economy based on mass (60.7% at maximum SO_2_ loading). Furthermore, the ammonium salt can be recharged with SO_2_ after use, allowing the IL to be reused in line with the principles of green chemistry principles.

**TABLE 1:** Atom economy and price for [NEt_3_Me][Cl(SO_2_)_3.66_], DABSO, SOgen, K_2_S_2_O_5_, and Na_2_S_2_O_4_.

	SO_2_	SO_2_‐IL	DABSO	SOgen	K_2_S_2_O_5_	Na_2_S_2_O_4_
**SO_2_ Equivalents**	1	3.66	2	1	2	1
**Atom Economy [%]**	100	60.7	53.3	11.7	57.6	36.8
**Price [€/g]** [53, 54, 55]	0.01	0.05	2.59	10.25	0.22	0.10
**Price [€/mol]** [53, 54, 55]	0.40	17.90	623.44	5635.37	48.91	18.25

The developed IL is also economically attractive, particularly given its low‐cost components and scalability. Although the sulfite salts for Na_2_S_2_O_4_ (0.10 €/g) and K_2_S_2_O_5_ (0.22 €/g) are in the same cost range as [NEt_3_Me][Cl(SO_2_)_3.66_] (0.05 €/g), the IL offers a higher atom economy. In contrast, DABSO (3.59 €/g) and SOgen (10.25 €/g) are significantly more expensive. The low price and high atom economy make it possible to use IL cost‐effectively even in reactions requiring a large excess of SO_2_.

### 2.5 Application as SO_2_‐Transfer Reagent

**SCHEME 1:**
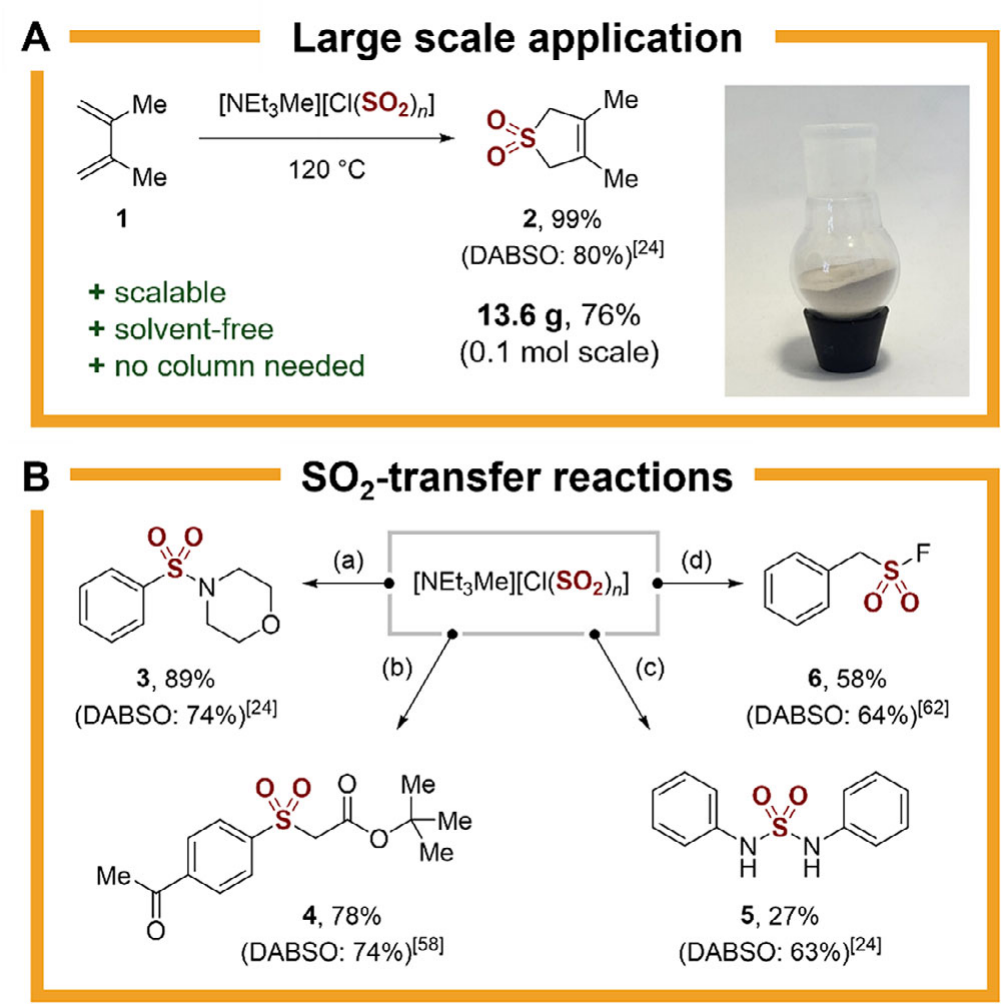
Versatile synthetic applications of [NEt_3_Me][Cl(SO_2_)*_n_*] for on‐demand SO_2_‐transfer. Yields refer to the product obtained after purification. The yields in parentheses have been reported utilizing DABSO as SO_2_‐transfer reagent. Reaction conditions: (A). [NEt_3_Me][Cl(SO_2_)_3.3_] (1.8 equiv.), 2,3‐dimethyl‐1,3‐butadiene (1.0 equiv.), neat, 120°C, 66 h (99% on 0.5 mmol scale, 76% on 122 mmol scale); (B). a) [NEt_3_Me][Cl(SO_2_)_3.3_] (1.5 equiv.), PhMgBr (1.0 equiv.) *then* SO_2_Cl_2_ (1.2 equiv.) *then* morpholine (10 equiv.), THF, –40°C to 23°C, 4 h (89%); b) [NEt_3_Me][Cl(SO_2_)_3_] (0.4 equiv.), 4‐iodoacetophenone (1.0 equiv.), Pd(OAc)_2_ (5.0 mol%), CataCXium A (8.0 mol%), NEt_3_ (3.0 equiv.), *i*‐PrOH, 75°C, 18 h, then *t*‐butyl bromoacetate (2.0 equiv.), DMF, RT, 3.5 h, (78%); c) [NEt_3_Me][Cl(SO_2_)_3_] (1.3 equiv.), aniline (2.0 equiv.), I_2_ (1.5 equiv.), MeCN, 0°C to RT, 15 h, (27%); d) [NEt_3_Me][Cl(SO_2_)_3_] (0.35 equiv.), BnMgCl (1.0 equiv.), NFSI (1.5 equiv.), THF, 0°C to RT, 5 h (58%).

Next, we investigated the potential application of [NEt_3_Me][Cl(SO_2_)*_n_*] as an inexpensive and easy‐to‐handle SO_2_‐transfer reagent toward the synthesis of organosulfur compounds. As illustrated in Scheme 1A, the synthesis of 3‐sulfolene **2** could be realized through [4+1]‐cheletropic reaction with 2,3‐dimethyl‐1,3‐butadiene (**1**) in a remarkable yield of 99%. Notably, the reaction affords the product in generally higher yields compared to DABSO (80%) [24] and could be easily performed on decagram‐scale (0.12 mol, equimolar ratio of both reactants) under solvent‐free conditions, affording **2** after recrystallization in a similar yield of 76% (13.6 g). The scalability of 3‐sulfolenes is highly desirable as they serve as useful synthetic intermediates and their hydrogenated congeners, sulfolanes, are commonly applied as high‐boiling, polar solvents in gas production and oil refining [56, 57].

Given the capacity of SO_2_ to rapidly engage in C─S bond formation with organometallic species, we elaborated this process for the on‐demand synthesis of sulfones and sulfonamides. It was found that exposure of [NEt_3_Me][Cl(SO_2_)_3.3_] to phenylmagnesium bromide (PhMgBr) at −40°C effectively generated a sulfinate intermediate, which could be converted into its corresponding sulfonyl chloride by the addition of sulfuryl chloride (SO_2_Cl_2_). The one‐pot procedure was terminated by the addition of morpholine (excess), giving rise to sulfonamide **3** in 89% yield based on PhMgBr as the limiting reagent. Importantly, our developed reagent provided the product in generally higher yields compared to DABSO (74% of **3**, Scheme 1B) [24]. Recently, the application of transition metal catalysis has broadened the scope of sulfonylation reactions omitting the necessity of hazardous and moisture‐sensitive organometallic reagents [27]. Preliminary work by Willis and coworkers has demonstrated the applicability of DABSO for the synthesis of non‐symmetrical sulfones via a Pd‐catalyzed cross‐coupling reaction [58]. When subjecting [NEt_3_Me][Cl(SO_2_)_3_] to the developed reaction conditions, sulfone **4** was obtained from 4‐iodoacetophenone and *tert*‐butyl bromoacetate in similar yields (78%) in comparison to DABSO (74%). In addition, twofold S−N bond formation with aniline occurred under oxidative reaction conditions employing iodine (I_2_) to yield *N*,*N*´‐diphenylsulfamide (**5**), albeit at lower yields than with DABSO as the transfer reagent [24]. Eventually, we turned our attention to the synthesis of sulfonyl fluorides, which have been identified as powerful functional handles for bioconjugation via sulfur(VI)‐fluoride exchange (SuFEx) processes [18, 59, 60, 61]. For this, the sulfinate species formed *in situ* from [NEt_3_Me][Cl(SO_2_)_3_] and benzylmagnesium chloride was reacted with *N*‐fluorobenzenesulfonimide (NFSI) to produce sulfonyl fluoride **6** in 58% yield, demonstrating that our developed reagent is a cost‐effective alternative to DABSO for SuFEx reagent synthesis [62].

## 3 Conclusion

In conclusion, we have discovered the ionic liquid [NEt_3_Me][Cl(SO_2_)*_n_*] capable of reversibly storing up to 3.66 equivalents of SO_2_ while maintaining a remarkably low vapor pressure, even at elevated temperatures. Solid‐state crystal structure and Raman studies indicate an interaction between chloride anion and SO_2_ moiety, resulting in a shift of the SO vibration. Further quantum chemical calculations were performed to analyse the bonding situation, and NBO analysis showed the shift of electron density from the orbital containing the chloride lone pair into the π*‐orbital of the S─O bond. QTAIM calculations classified this bond as a covalent bond with strong charge‐shift character. Its long‐term stability and low viscosity, makes the ionic liquid highly attractive for applications as a convenient, safe, and easy‐to‐handle SO_2_ transfer reagent. In this context, [NEt_3_Me][Cl(SO_2_)*_n_*] was successfully employed in the synthesis of 3‐sulfolenes, sulfonamides, and SuFEx reagents, establishing it as a practical alternative to commonly used SO_2_ surrogates. Furthermore, its high atom economy, low cost, and the intrinsic ability of an ionic liquid to function as a solvent, render it particularly appealing for large‐scale applications.

## Conflicts of Interest

The authors declare no conflicts of interest.

## Supporting information



The authors have cited additional references within the Supporting Information [63, 64, 65, 66, 67, 68, 69, 70, 71, 72, 73, 74, 75, 76, 77, 78, 79, 80, 81, 82, 83, 84, 85]. Deposition Number(s)  2528652 (for [NEt_3_Me][Cl(SO_2_)]), 2528646 (for [PNP][Cl(SO_2_)]•CH_2_Cl_2_), 2528647 (for [PPh_4_][Cl(SO_2_)_2_].), 2528651 (for [NEt_4_][Cl(SO_2_)_3.5_]_2_) contain the supplementary crystallographic data for this paper. These data are provided free of charge by the joint Cambridge Crystallographic Data Centre and Fachinformationszentrum Karlsruhe. **Supporting File 1**: chem71160‐sup‐0001‐SuppMat.pdf.

**Supporting File 2**: chem71160‐sup‐0002.cif.

**Supporting File 3**: chem71160‐sup‐0003.cif.

**Supporting File 4**: chem71160‐sup‐0004.cif.

**Supporting File 5**: chem71160‐sup‐0005.cif.

## Data Availability

The data that support the findings of this study are available from the corresponding author upon reasonable request.

## Footnotes

1 Literature reports of solid‐state structures of chlorosulfites from early studies do exist, however, due to their poor resolution, the structural parameters are not in agreement with our results.
