# Supplementary material for: SO2 Transfer Enabled by an Easy‐to‐Handle Ionic Liquid
Source: Chemistry. 2026 May 29;32(34):e71160. doi: 10.1002/chem.71160 (PMC13569962; doi:10.1002/chem.71160)
Supplement: Supplementary file 1 [file CHEM-32-e71160-s002.pdf]

# Supporting Information: $SO_2$ Transfer Enabled by an Easy-to-Handle Ionic Liquid

Johanna S. Sturm,[a] Letizia Lanfredi,[b] Severin Erbertseder,[a] Marius Balizs,[a] Patrick Voßnacker,[a] Simon Steinhauer,[a] Merlin Kleoff,[a] Anja Wiesner,[a] Niklas Limberg,[a] Patrick Pröhm,[a] Franz-Lucas Haut,*[b] and Sebastian Riedel*[a]

[a] Johanna S. Sturm, Severin Erbertseder, Marius Balizs, Dr. Patrick Voßnacker, Dr. Simon Steinhauer, Dr. Merlin Kleoff, Dr. Anja Wiesner, Niklas Limberg, Dr. Patrick Pröhm, Prof. Dr. Sebastian Riedel
Freie Universität Berlin
Institut für Chemie und Biochemie, Anorganische Chemie
Fabeckstr. 34/36, 14195 Berlin
E-mail: s.riedel@fu-berlin.de
[b] Letizia Lanfredi, Dr. Franz-Lucas Haut
Freie Universität Berlin
Institut für Chemie und Biochemie, Organische Chemie
Takustr. 3, 14195 Berlin
E-mail: f.haut@fu-berlin.de

## Table of Content

1. Materials and Methods ..... 3

1.1 Materials ..... 3

1.2 Methods ..... 4

2. Physicochemical Properties ..... 7

2.1 Synthesis of $[NEt_3Me][Cl(SO_2)_n]$ ..... 7

2.2 Characterization of $[NEt_3Me][Cl(SO_2)_n]$ ..... 7

3. Quantum Chemical Calculations ..... 19

3.1 Chloride Ion Affinity (CIA) ..... 19

3.2 Structure Optimization and Vibrational Analysis ..... 21

3.3 Natural Bond Orbital Analysis (NBO) ..... 21

3.4 Electrostatic Potential Maps (ESP) ..... 22

3.5 Quantum Theory of Atoms in Molecules (QTAIM) ..... 23

4. $SO_2$ Transfer Reactions ..... 24

4.1 3,4-Dimethyl-2,5-dihydrothiophene 1,1-dioxide (2) ..... 24

4.2 Temperature and $SO_2$ Loading Dependence ..... 25

4.3 Decagram-Scale Synthesis of 2 ..... 26

4.4 4-(Phenylsulfonyl)morpholine (3) ..... 27

4.5 *Tert*-butyl 2-((4-acetylphenyl)sulfonyl)acetate (4) ..... 28

4.6 Synthesis of *N,N′*-Diphenylsulfamide (5) ..... 29

4.7 Synthesis of Phenylmethanesulfonyl Fluoride (6) ..... 30

5. Appendix ..... 31

5.1 Crystallographic Data ..... 31

5.2 NMR Spectra ..... 35

5.3 Coordinates of Optimized Structures ..... 42

5.4 Literature ..... 43

# 1. Materials and Methods

## 1.1 Materials

Sulfur dioxide (3.8, CAS: 7446-09-5, product no.: 10114006) was purchased from Linde GmbH. Phenylmagnesium bromide (CAS: 100-58-3, product no.: 331376, 1 mol/L solution in tetrahydrofurane), $Pd(OAc)_2$ (CAS: 3375-31-3, product no.: 520764), benzyl magnesium chloride (CAS: 6921-34-2, product no.: 302759, 1 mol/L solution in diethyl ether) were purchased from Sigma-Aldrich. Triethylmethylammonium chloride (CAS: 10052-47-8, product no.: AB139055), iodine (CAS: 7553-56-2, product no.: AB109411), di(1-adamantyl)-*n*-butylphosphine (CataCXium A®, CAS: 321921-71-5, product no.: AB152832), *t*-butyl bromoacetate (CAS: 5292-43-3, product no.: AB109214) were purchased from abcr GmbH Deutschland. Aniline (CAS: 62-53-3, product no.: 10444321) was purchased from Thermo Scientific. 2,3-Dimethylbuta-1,3-diene (CAS: 513-81-5) was purchased from Alfa Aesar (product no.: L04207.18) or TCI (product no.: D1148). *N*-Fluorobenzenesulfonimide (NFSI) (CAS: 133745-75-2, product no.: BD17056 98%) was purchased from BLD pharm.

Solvents, reagents and starting materials were used as received unless otherwise stated. Water is deionized, and solvents were anhydrous unless stated otherwise. Anhydrous tetrahydrofurane (THF) and dichloromethane ($CH_2Cl_2$) were utilized after purification with a MBraun SPS-800 solvent system employing solvents of HPLC grade purchased from Fisher Scientific and Carl Roth and were dried using activated 4 Å molecular sieves for at least 48 h prior to use. *i*-Propyl alcohol (IPA) was purchased from VWR Chemicals in analytical grade (≥99.7%) and dried using activated 4 Å molecular sieves for at least 48 h prior to use. Anhydrous acetonitrile (MeCN) and *N,N*-dimethylformamide (DMF) were purchased from Fisher Scientific. $NEt_3$ was distilled from $CaH_2$ and stored under argon over KOH to obtain dry. Triethylmethylammonium chloride $[NEt_3Me]Cl$ was dried under reduced pressure at 120 °C for 20 h.

Solvents for extraction, crystallization and flash column chromatography such as ethyl acetate (EtOAc, supplier: VWR Chemicals), *n*-pentane (supplier: Sigma-Aldrich), petroleum ether (PE, 40-60 °C, supplier: VWR Chemicals) and diethyl ether ($Et_2O$, Fisher) were purchased in technical grade and distilled under reduced pressure (rotary evaporator, 40 °C) prior to use. Methyl *tert*-butyl ether (MTBE) was purchased from Sigma-Aldrich in analytical grade and used without further purification.

## 1.2 Methods

### 1.2.1 General Information

Unless indicated otherwise, all experiments were carried out at room temperature and under inert conditions using standard Schlenk techniques and an argon atmosphere. Glassware was pre-dried before usage and greased with Triboflon III. Substances sensitive to hydrolysis and air were stored within a glovebox from Sylatech or MBraun ($O_2$ < 0.5 ppm, $H_2O$ < 0.5 ppm) under argon atmosphere. Air- and moisture-sensitive liquids were transferred via syringe through rubber septa. Addition of solids occurred under argon counter flow, or they were dissolved in appropriate solvents prior to use. Room temperature (RT) refers to temperatures between 18 °C and 23 °C. Reactions at low temperatures were carried out in a Dewar vessel filled with a cooling agent: *i*-propyl alcohol/dry ice (−78 °C), acetonitrile/dry ice (–40 °C) or distilled water/ice (0 °C). Reaction temperatures above RT were conducted in a preheated oil bath. The reactions were magnetically stirred and monitored by $^1$H NMR spectroscopy or analytical thin-layer chromatography (TLC) using aluminum plates precoated with silica gel (0.20 mm, 60 Å pore size, Merck, impregnated with a fluorescent indicator (254 nm)). TLC plates were visualized by exposure to ultraviolet light (UV), were stained by submersion in *p*-anisaldehyde solution or aq. potassium permanganate solution ($KMnO_4$) and were developed by heating with a heat gun.

**Flash-column chromatography** was carried out using silica gel (Macherey-Nagel, pore size 60 Å, 0.04-0.063 mm). The yields refer to chromatographically and spectroscopically ($^1$H and $^{13}$C NMR) pure material.

### 1.2.2 Analytics

**NMR** spectra of the ionic liquid [$NEt_3Me$][$Cl(SO_2)_n$] were recorded on a *JEOL 400 MHz ECS* or *JEOL 400 MHz ECZ* spectrometer. NMR spectra of the ILs application in organic chemistry were acquired using a *Bruker Avance 500 MHz* or *Bruker Avance 700 MHz* spectrometers. $^{19}$F NMR spectra were conducted using *JEOL ECX 400 MHz*. All NMR spectra were recorded at room temperature (20 °C). Chemical shifts (δ) are given in parts per million (ppm) and referenced to $CD_2Cl_2$ ($^1$H: 5.32 ppm referring to residual $CH_2Cl_2$, $^{13}$C: 53.8 ppm) or $CDCl_3$ ($^1$H: 7.26 ppm referring to residual $CHCl_3$, $^{13}$C: 77.2 ppm). To avoid solvent interaction, flame sealed capillaries (ACCAP), including $(CD_3)_2CO$ as external lock and $^1$H NMR standard ($^1$H: 2.05 ppm referring to residual $(CH_3)_2CO$), were used. Coupling constants (*J*) are given in Hertz (Hz) and refer to apparent multiplicities (s = singlet, bs = broad singlet, d = doublet, bd = broad doublet, t = triplet, q = quartet, m = multiplet, dd = doublet of doublets, etc.). The $^1$H NMR spectra are reported as follows: chemical shift (multiplicity, coupling constants, number of protons). All raw fid files were processed, and the spectra analyzed using the software 14.3 from Mestrelab Research SL.

**Raman** spectra were recorded using the FT-Raman spectrometer *MultiRAM* from Brucker with an excitation wavelength of 1064 nm (Nd:YAG laser). The spectrometer is equipped with a germanium detector, which was cooled with liquid nitrogen. All spectra were measured at room temperature, at 50-100 mW laser power and a spectral resolution of 4 $cm^{-1}$. The measuring range was set between 4000 $cm^{-1}$ to 50 $cm^{-1}$. OPUS 7.5 and Origin 2025 were used to visualize and analyze the spectra.

**Gravimetric measurements** were carried out with a *360ES* precision balance by Precisa Gravimetrcis AG.

**Viscosity measurements** were done with a *Ubbelohde* viscosimeter from *SCHOTT MAINZ*.

**X-Ray Diffraction** data were collected from a single crystal at 100 K on a *Bruker D8 VENTURE* dual wavelength Mo/Cu three-circle diffractometer with a microfocus sealed X-ray tube using a mirror optics as monochromator and a *Bruker PHOTON II* detector. The diffractometer was equipped with an *Oxford Cryostream 700* low temperature device and used Mo$K_\alpha$ radiation (λ = 0.71073 Å). Crystals were mounted on a MiTeGen micromount with perfluoroether oil. All data were integrated with SAINT V8.40B and a multi-scan absorption correction using SADABS 2016/2 was applied.[1,2] The structures were solved with OLEX2.15 by SHELXT 2018/2 and refined by using SHELXL-2018/3.[3,4] Crystallographic data for the structures reported in this paper have been deposited with

the Cambridge Crystallographic Data Centre.[5] CCDC 2528652, 2528646, 2528647 and 2528651 contain the supplementary crystallographic data for this paper. These data can be obtained free of charge. This report and the CIF file were generated using FinalCif.[6]

**Conductivity measurements** were carried out with a *SevenCompact Conductivity S230* conductivity meter from *METTLER TOLEDO* and an *inLab 710* sensor.

### 1.2.3 Computational Details

XYZ coordinates were generated with Avogadro 1.2.[7] All calculations were conducted on the high-performance computing system CURTA by ZEDAT, FU Berlin.[8] Structure optimizations with subsequent frequency calculations were done with Orca 5.0.4 on the B3LYP def2-QZVPPD level of density functional theory with Grimmes's D4 dispersion correction.[9–12] Electrostatic potential was calculated with the Gaussian 16 program at the B3LYP-D3BJ def2-QZVPPD level of DFT and plotted with VMD 19.2.[13,14] NBO analysis was performed with the NBO 7.0 program in conjunction with Gaussian 16 with optimized structures obtained by Orca 5.0.4.[15] The resulting NBOs were plotted with ChemCraft 1.8.[16] QTAIM analysis and topology maps were calculated and created with Multiwfn 3.8.[17] Optimized structures were edited with Diamond 4.6.4 software.[18]

# 2. Physicochemical Properties

## 2.1 Synthesis of $[NEt_3Me][Cl(SO_2)_n]$

$$[NEt_3Me]Cl + n\,SO_2 \longrightarrow [NEt_3Me][Cl(SO_2)_n]$$

Triethylmethylammonium chloride $[NEt_3Me]Cl$ was placed in a Rettberg flask which was then evacuated and weighed. Upon addition of sulfur dioxide gas, the colorless salt underwent an exothermic reaction and turned into a low viscous, colorless liquid. $SO_2$ was added until the desired vapor pressure equilibrium was reached while constantly stirring the resulting liquid. If not stated otherwise, the vapor pressure of $SO_2$ was adjusted to 1 bar. The amount of absorbed $SO_2$ in the sulfite $[NEt_3Me][Cl(SO_2)_{3.6}]$ was determined gravimetrically. For organic follow up chemistry, the IL was loaded with 800 mbar with $[NEt_3Me][Cl(SO_2)_{3.3}]$ for better handling.

**$^1$H NMR** (400 MHz, $CD_2Cl_2$): δ [ppm] = 3.34 (q, $^3J$ = 7.3 Hz, 6H), 2.97 (s, 3H), 1.32 (tt, $^3J$ = 7.3 Hz, $^3J$ = 2.0 Hz, 9H).

**$^{13}$C NMR** (101 MHz, $CD_2Cl_2$): δ [ppm] = 56.3 (t, $^2J$ = 3.0 Hz), 47.2 (t, $^2J$ = 4.2 Hz), 7.9.

**Raman** (liquid, 1064 nm, 25 °C): $\tilde{\nu}$ [$cm^{-1}$] = 2987 (m), 2950 (m), 1460 (w), 1311 (w), 1143 (s), 683 (w).

## 2.2 Characterization of $[NEt_3Me][Cl(SO_2)_n]$

### 2.2.1 Solid State Structure Determination

$CH_2Cl_2$ was stepwise (0.1 mL) added to a frozen sample of $[NEt_3Me][Cl(SO_2)]$ (1.14 g, 5.30 mmol) at −80 °C. The mixture was thawed, refrozen and again mixed with $CH_2Cl_2$ until a saturated solution at −80 °C had formed. After keeping the solution at −80 °C for several days, colorless crystals were obtained and analyzed by X-ray diffractometry.

Since it was not possible to obtain crystals of $[NEt_3Me][Cl(SO_2)_n]$ with more than one $SO_2$ equivalent, larger cations such as $[NEt_4]^+$, $[PNP]^+$ and $[PPh_4]^+$ were used as counterions for crystallization. The corresponding chloride salts were loaded with $SO_2$ by condensing the gas onto the samples in different stoichiometric amounts at −196 °C. Small amounts of $CH_2Cl_2$ were added to the ionic liquids and the solution was stored at −80 °C until colorless crystals were obtained and analyzed by X-ray diffractometry (Figure S1-S4).

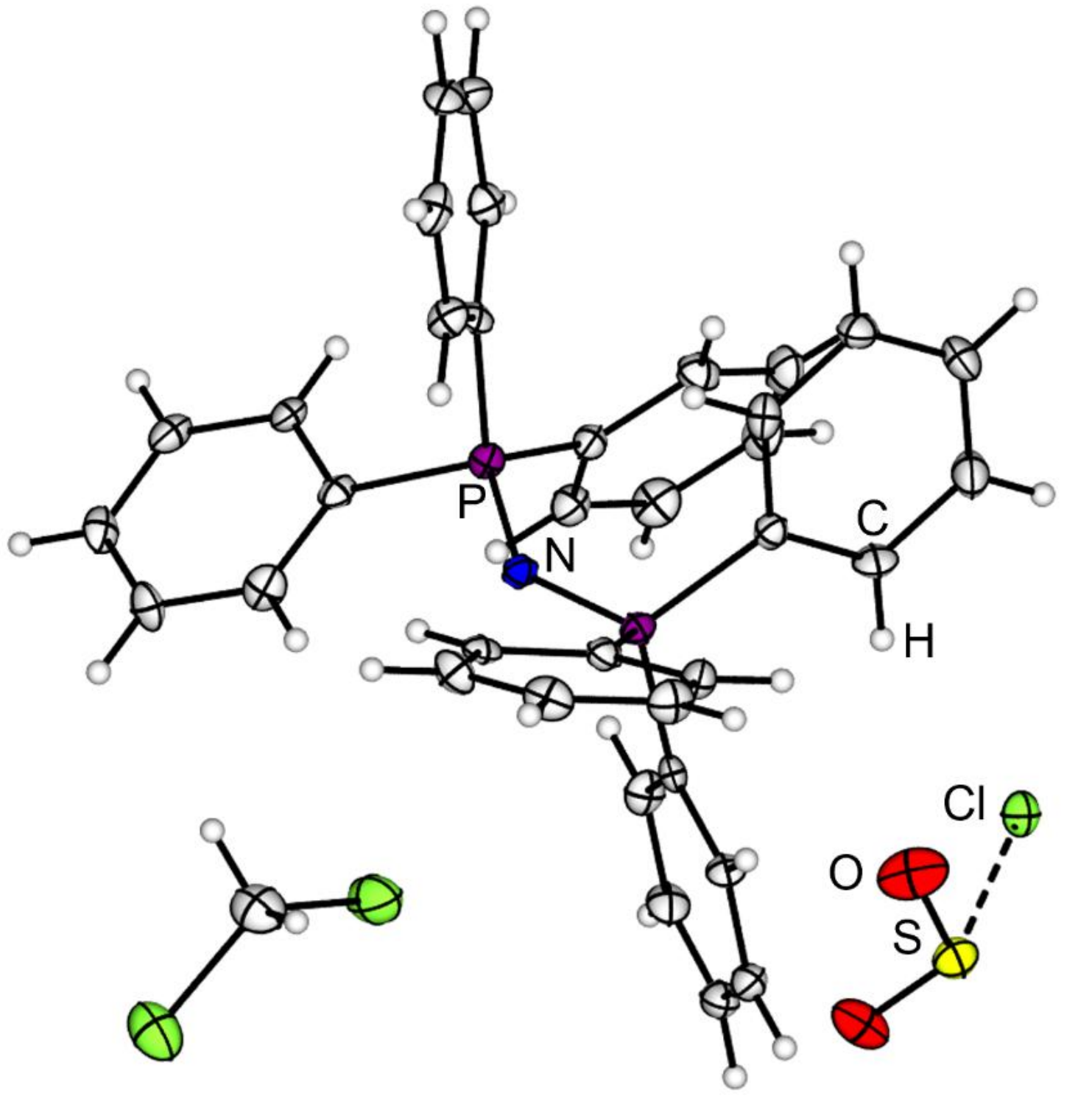


**Figure S1**: Molecular structure of [PNP][Cl(SO$_2$)]•CH$_2$Cl$_2$ in the solid state with a molecule of CH$_2$Cl$_2$ co-crystallized, obtained from CH$_2$Cl$_2$ solution. Displacement ellipsoids are set at 50% probability. Obtained bond lengths [pm]: S−O 143.6(4)-144.4(5), Cl−S 247.4(4).

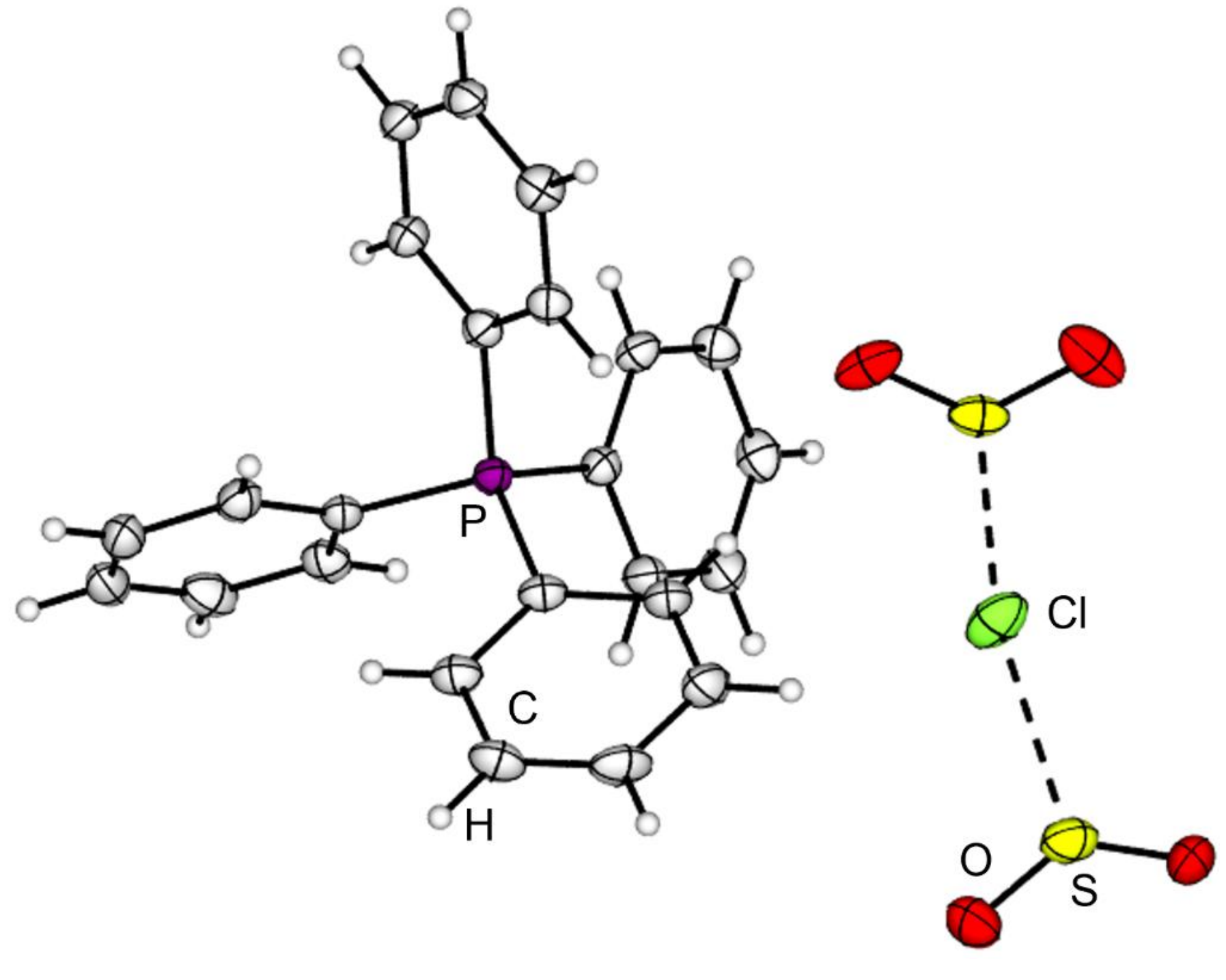


**Figure S2**: Molecular structure of [PPh$_4$][Cl(SO$_2$)$_2$] in the solid state, obtained from CH$_2$Cl$_2$ solution. Displacement ellipsoids are set at 50% probability. Obtained bond lengths [pm]: S−O 143.3(2)-143.6(2), Cl−S 259.9(4), bond angle Cl−S−Cl 168.8(1)°.

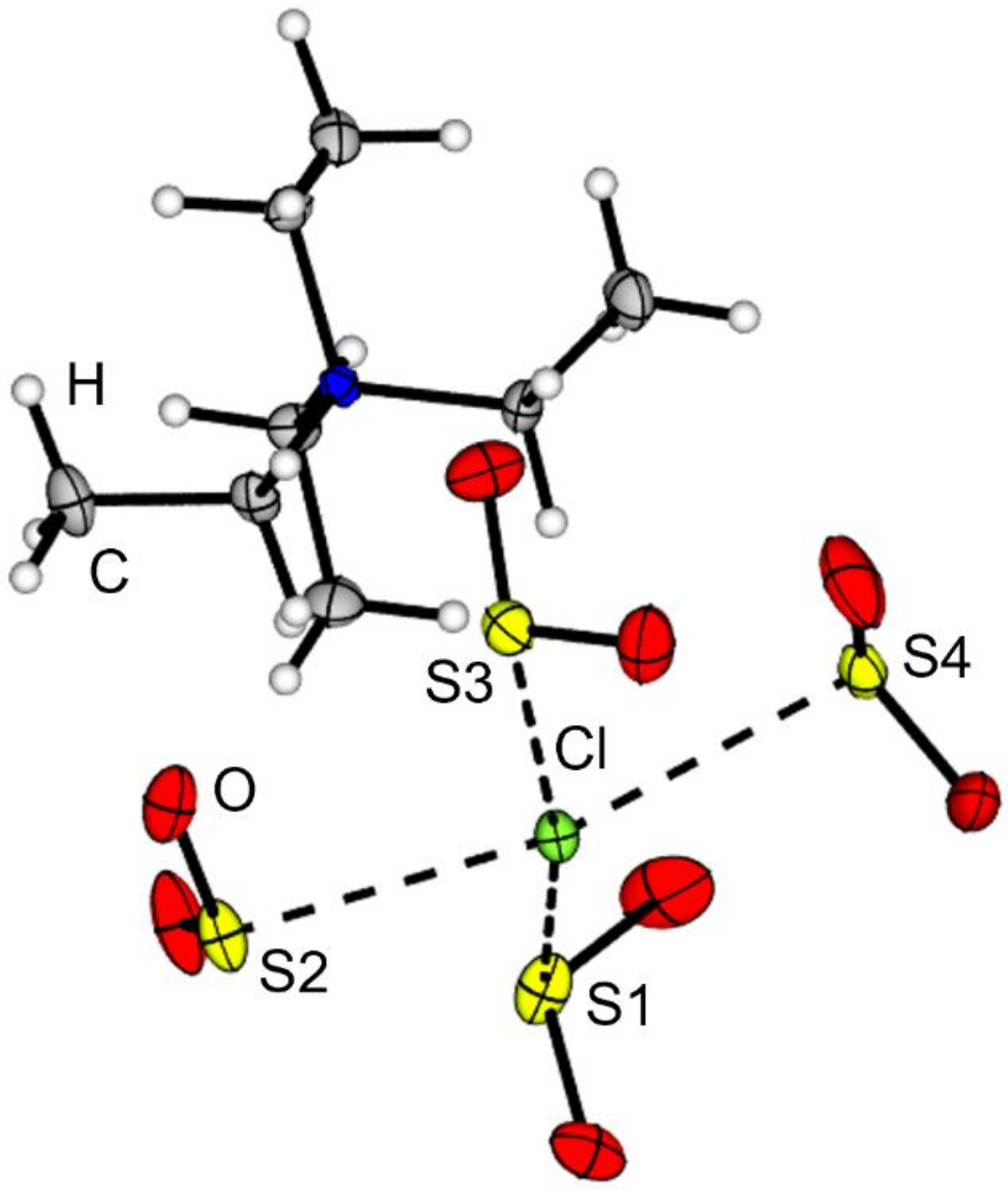


**Figure S3**: Molecular structure of $[NEt_4][Cl(SO_2)_{3.5}]_2$ in the solid state. Disorder omitted for clarity. Displacement ellipsoids are set at 50% probability. Obtained bond lengths [pm]: S−O 140.3(4)-148.5(11), Cl−S1 286.1(1), Cl−S2 287.7(1), Cl−S3 330.6(1), Cl−S4 287.4(1), bond angle Cl−S3−Cl 156.3(1)°.

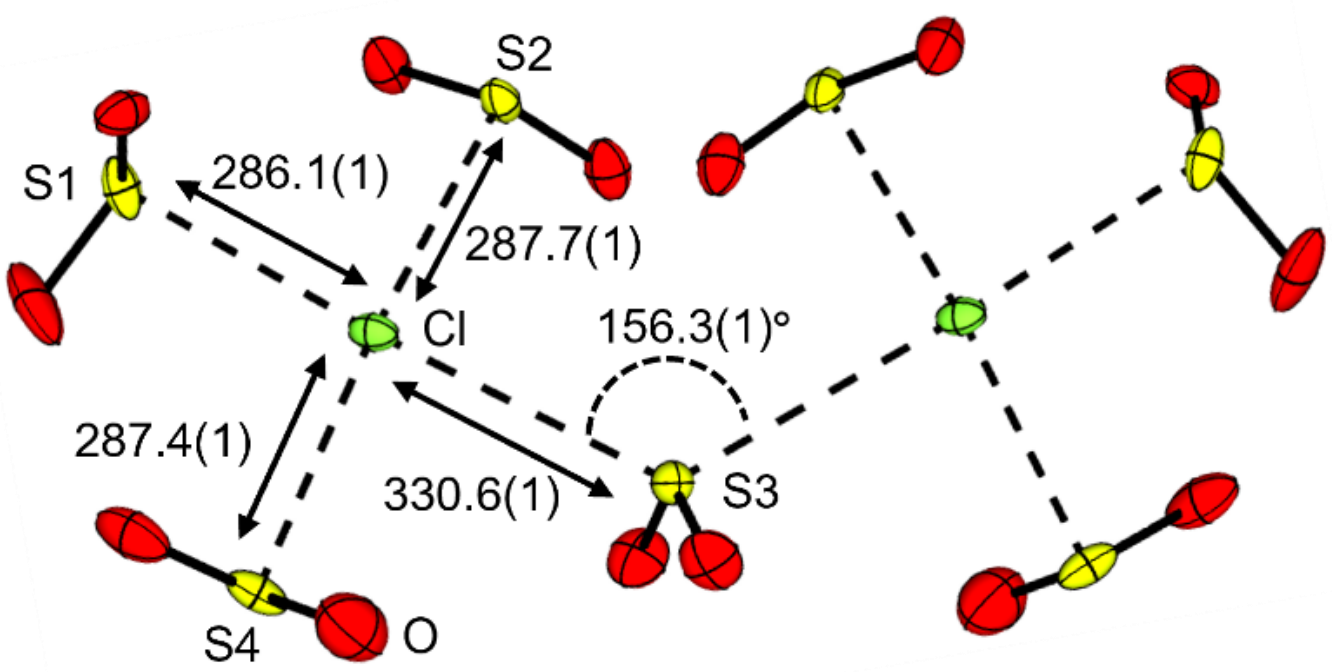


**Figure S4**: Extension on the molecular structure in solid state showing $[Cl_2(SO_2)_7]^{2-}$ of 2 $[NEt_4][Cl(SO_2)_{3.5}]$ units without cation and disorder. Displacement ellipsoids are set at 50% probability. Bond distances given in pm.

### 2.2.2 Vapor Pressure

Vapor pressure curves were determined at temperatures of 20, 50 and 80 °C by varying the amount of $SO_2$. $[NEt_3Me]Cl$ (10.0 g, 65.9 mmol) was filled into a 100 mL Rettberg flask. The gas was gradually added to the ammonium salt in steps of 50 to 100 mbar. After the equilibration had been established the vapor pressure was recorded and the amount of $SO_2$ was determined gravimetrically. Remaining $SO_2$ in the gas phase was calculated using the ideal gas law and subtracted. After reaching an equilibrium vapor pressure of 1 bar, $SO_2$ was removed under reduced pressure following the same procedure.

The maximum amount of absorbed gas at 1 bar was investigated by condensing $SO_2$ onto the IL and subsequently releasing gas until an equilibrium of 1 bar was established. [$NEt_3Me$]Cl (5.84 g, 38.5 mmol, 1.00 equiv) was placed in a 100 mL Rettberg flask and loaded with $SO_2$ until an equilibrium pressure of 1 bar was achieved. Additional $SO_2$ (0.933 g, 14.5 mmol) was condensed onto it at −196 °C. After allowing the liquid to warm to room temperature a bubbler was added to the apparatus and the excess $SO_2$ was released. The adsorbed $SO_2$ (9.02 g, 141 mmol, 3.66 equiv) was determined gravimetrically and corrected by the amount of $SO_2$ in the gas phase.

For the determination of the temperature dependent vapor pressure the ionic liquid was filled into a glass autoclave connected to a digital manometer. The sealed vessel was heated stepwise (Figure S5). The temperature was held constant for about 20 min before the equilibrium vapor pressure was measured.

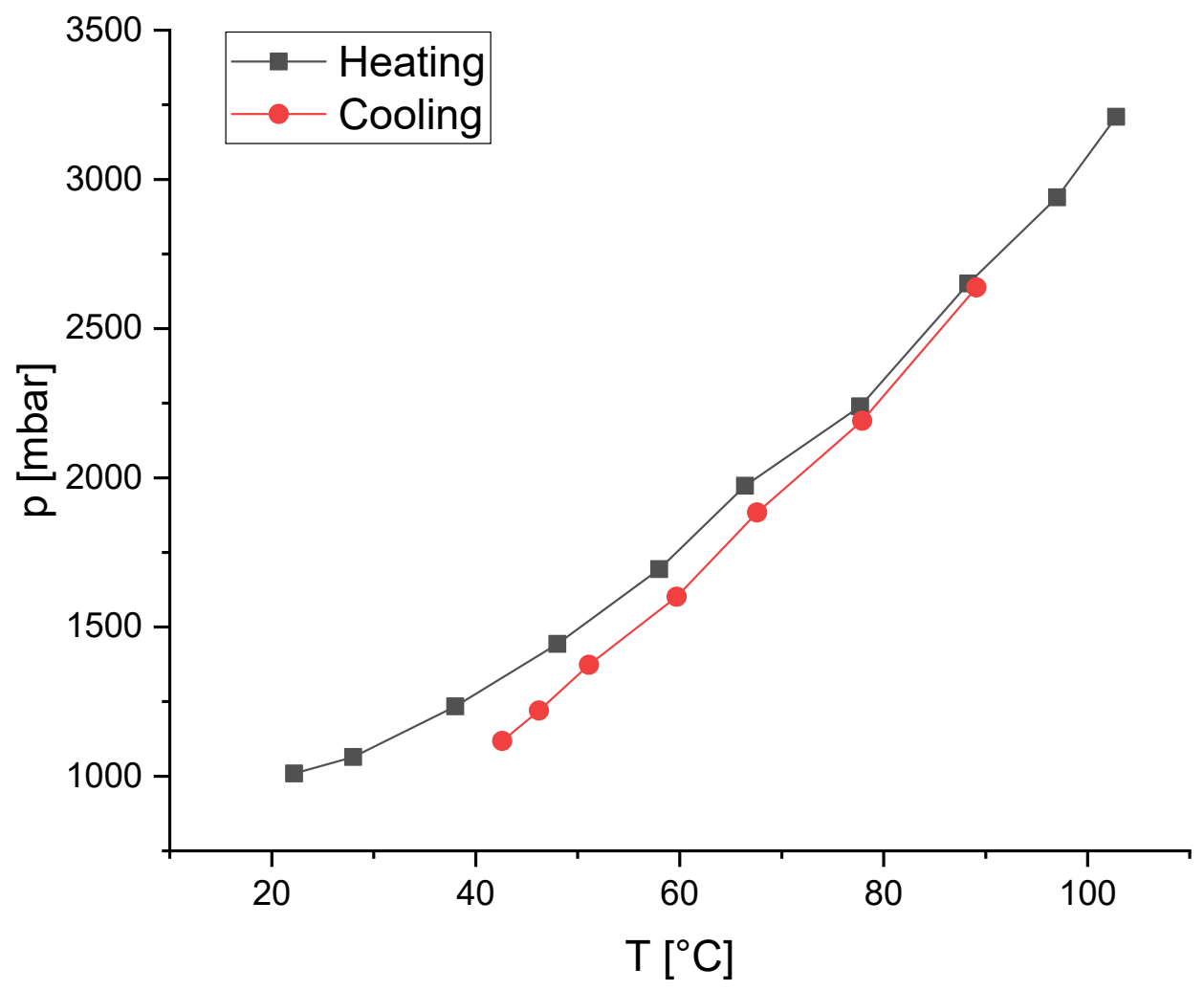


**Figure S5**: Temperature dependence of the vapor pressure of [$NEt_3Me$][$Cl(SO_2)_n$].

In order to assess the impact of water, the storage capacity of [$NEt_3Me$]Cl (9.92 g, 65.4 mmol) and water (1.18 g, 65.4 mmol, 1.00 equiv) at 20 °C was determined. For this, the ammonium chloride was loaded until the vapor pressure reached 1 bar. Then, the amount of $SO_2$ was gravimetrically determined and corrected by the amount of $SO_2$ in the gas phase revealing a storage capacity of 3.48 equiv $SO_2$. This observation showcases a modest impact of water on the $SO_2$ storage capacity of [$NEt_3Me$]Cl.

### 2.2.3 Cyclic Performance

[$NEt_3Me$][$Cl(SO_2)_n$] (9.9 g, 65.2 mmol) were added into a 50 mL Schlenk flask with bubbler on top. The flask was evacuated and the salt loaded with $SO_2$. The flask was then heated to 80 °C and the valve to the bubbler was opened to let the $SO_2$ evaporate.

The amount of loaded and released $SO_2$ was determined gravimetrically. This process was repeated for five cycles (Figure S6).

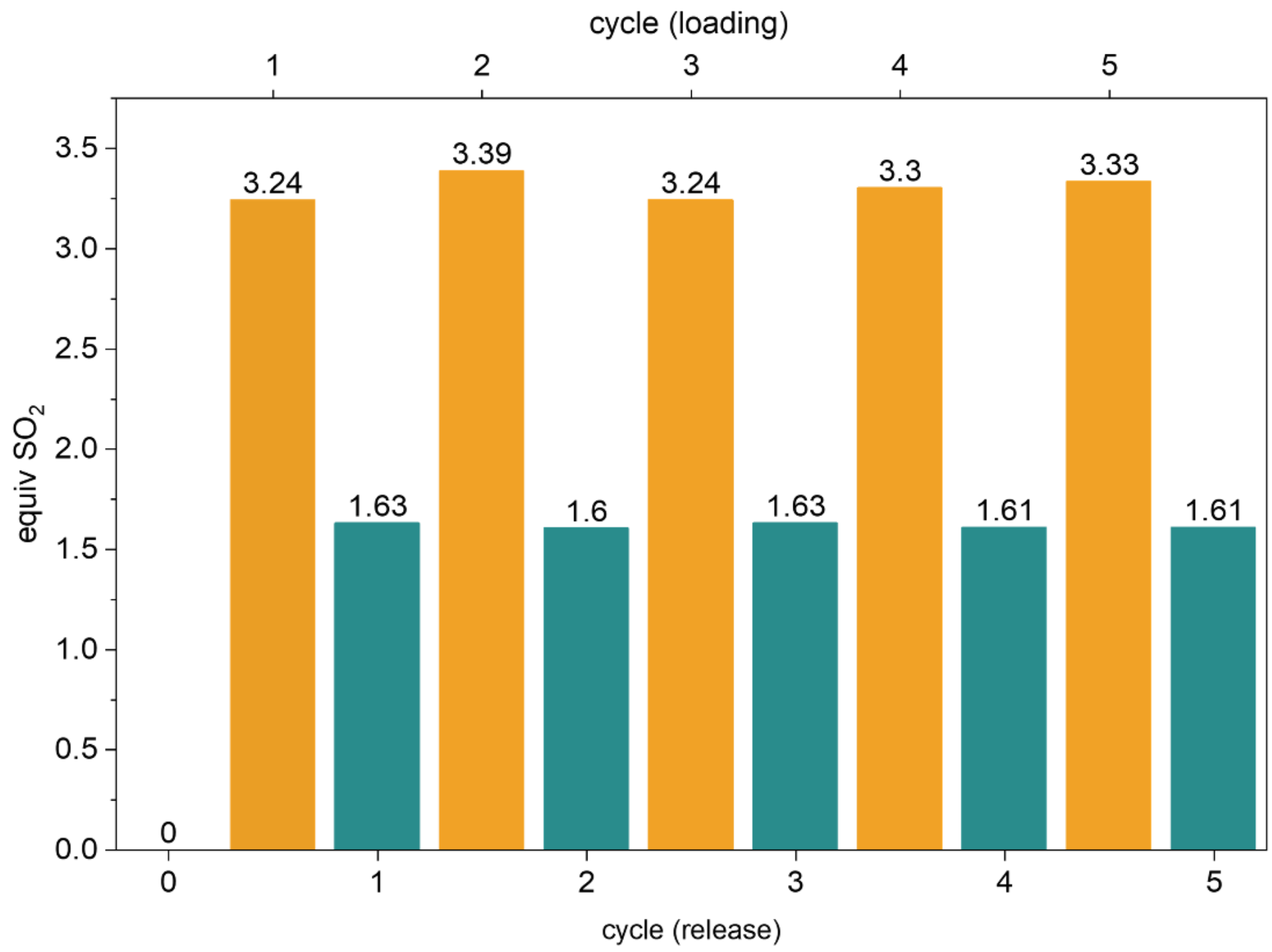


**Figure S6**: Cyclic performance of [$NEt_3Me$][$Cl(SO_2)_n$] for loading of $SO_2$ at 20 °C and $SO_2$ release performed at 80 °C.

### 2.2.4 Raman and NMR Studies

Four 8 mL Rettberg tubes were filled with [$NEt_3Me$]Cl (1.10 g, 7.25 equiv) and gravimetric determined amounts of $SO_2$ were then condensed onto the salts at −196 °C. The samples were warmed up to room temperature and characterized with Raman and NMR spectroscopy (Table S1, Figures S7-S12).

**Table S1**: Stoichiometric composition of the samples prepared for Raman measurements.

| | **Sample 1** | **Sample 2** | **Sample 3** | **Sample 4** |
|---|---|---|---|---|
| **Equiv ($SO_2$)** | 1.00 | 2.00 | 3.00 | 4.00 |
| **n($SO_2$) [mmol]** | 7.25 | 14.5 | 21.8 | 29.0 |
| **m($SO_2$) [g]** | 0.467 | 0.929 | 1.40 | 1.84 |

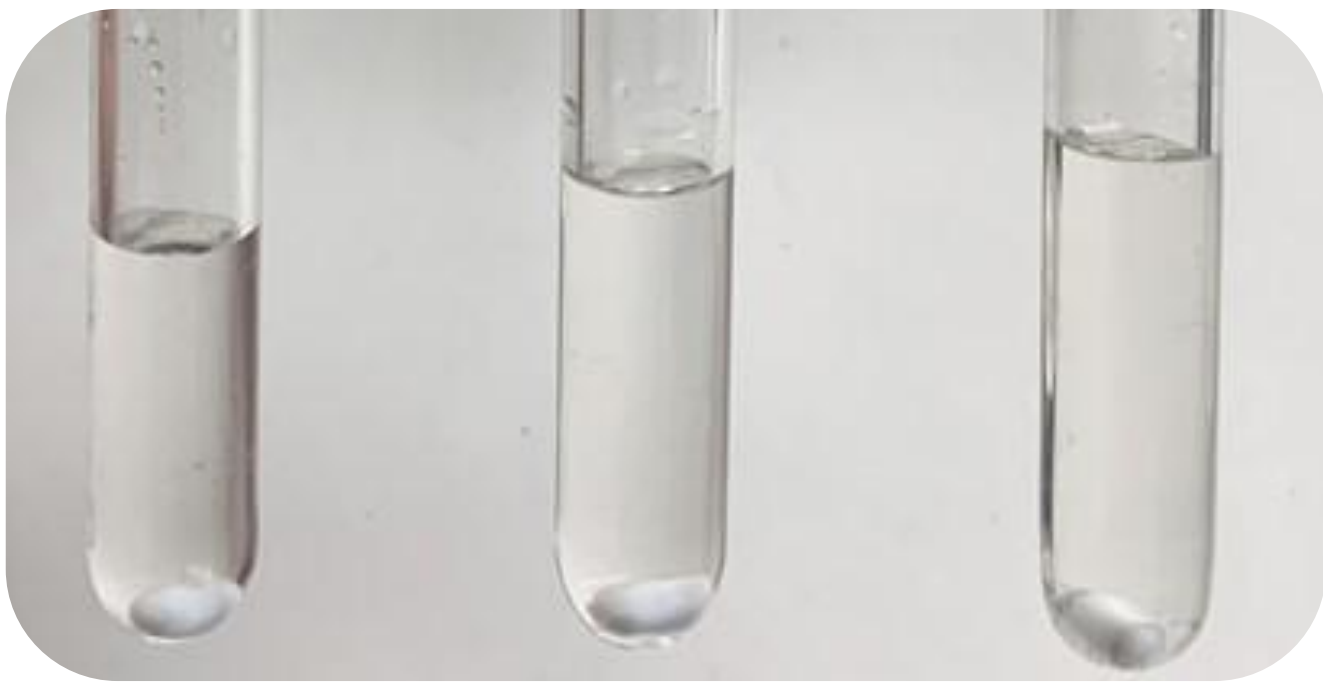

**Figure S7**: Rettberg tubes containing [NEt$_3$Me][Cl(SO$_2$]$_n$] with 1 equiv (left), 2 equiv (middle), and 3 equiv (right) SO$_2$.

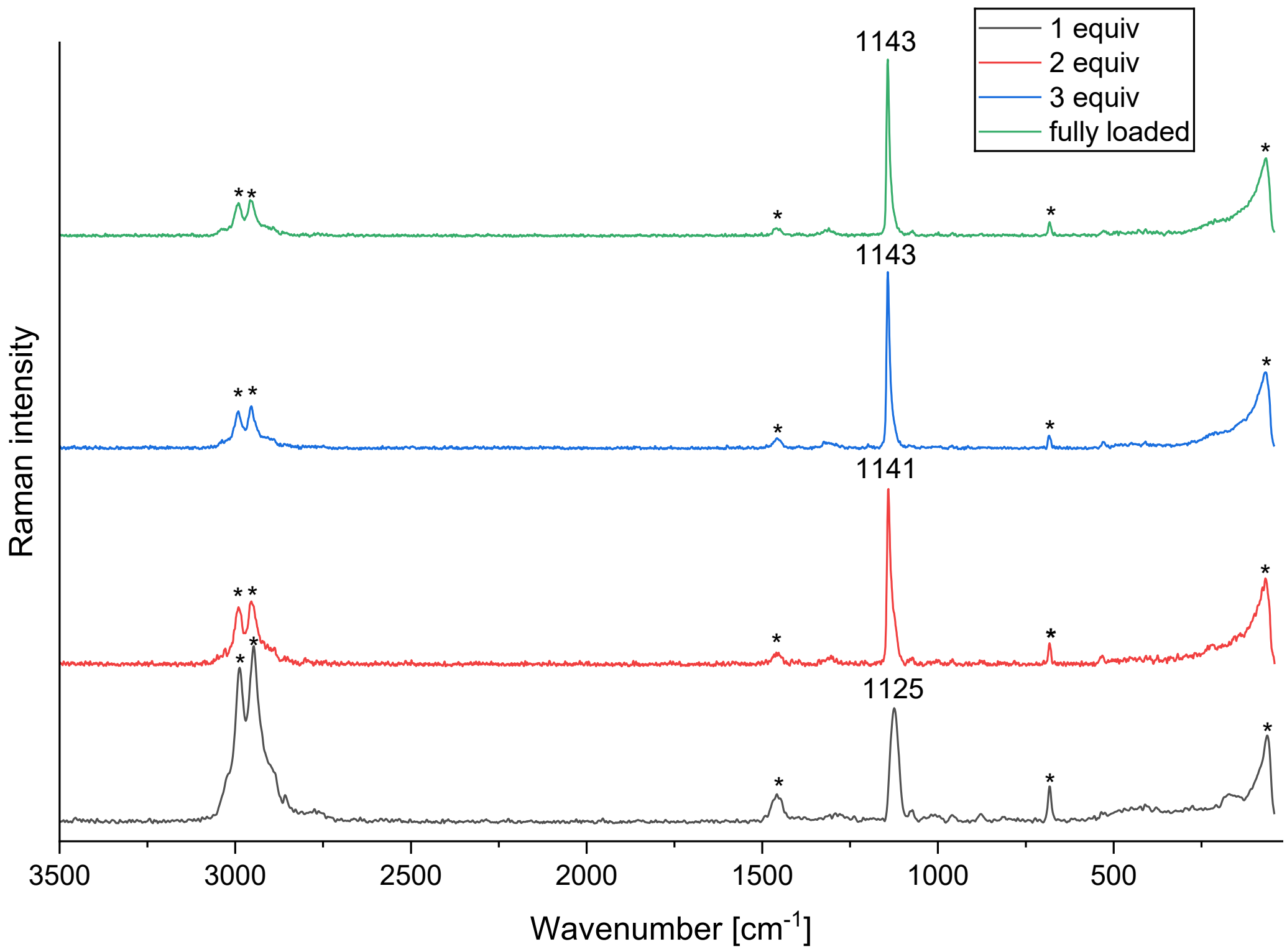


**Figure S8**: Raman spectra of [NEt$_3$Me][Cl(SO$_2$)] (grey), [NEt$_3$Me][Cl(SO$_2$)$_2$] (red), [NEt$_3$Me][Cl(SO$_2$)$_3$] (blue) and [NEt$_3$Me][Cl(SO$_2$)$_{3.66}$] (green). Bands marked with an * belong to the cation.

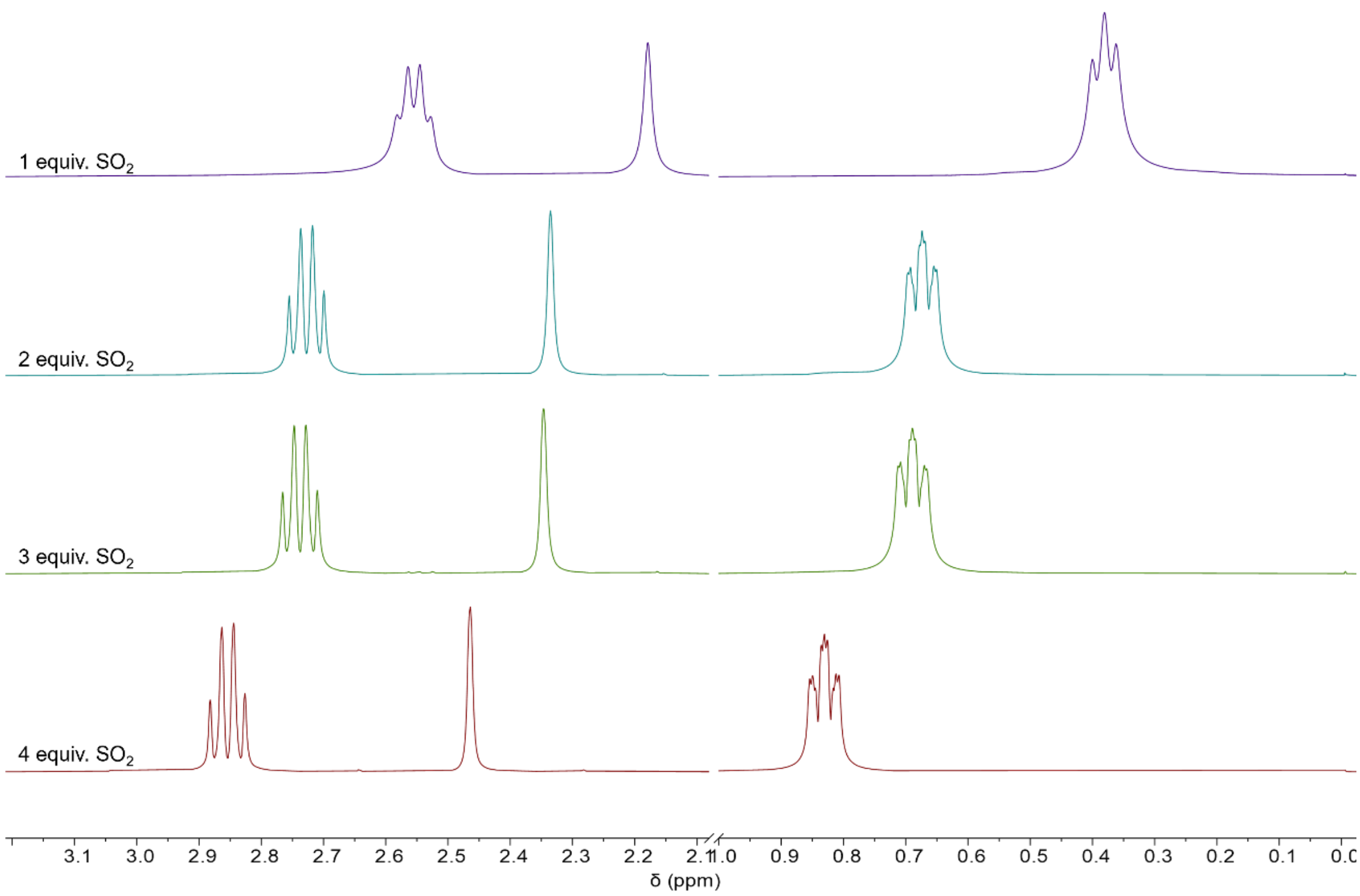


**Figure S9**: $^{1}$H NMR spectra of $[NEt_3Me][Cl(SO_2)]$ (purple), $[NEt_3Me][Cl(SO_2)_2]$ (blue), $[NEt_3Me][Cl(SO_2)_3]$ (green) and $[NEt_3Me][Cl(SO_2)_{3.66}]$ (red). ACCAP capillaries were used for reference.

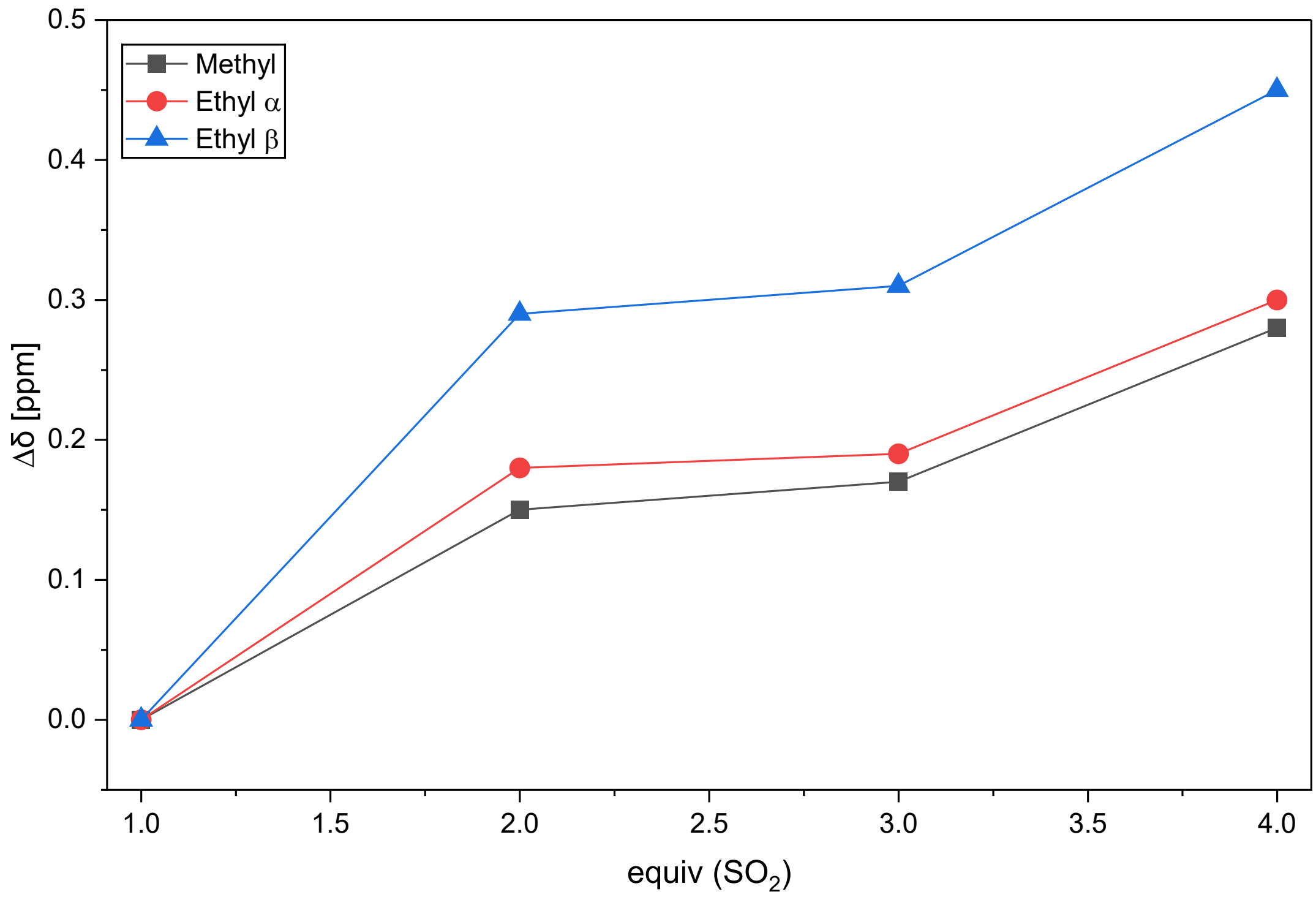


**Figure S10**: Difference in chemical shift of the $^{1}$H NMR signals of the IL with different loadings in comparison to the loading with 1 equiv.

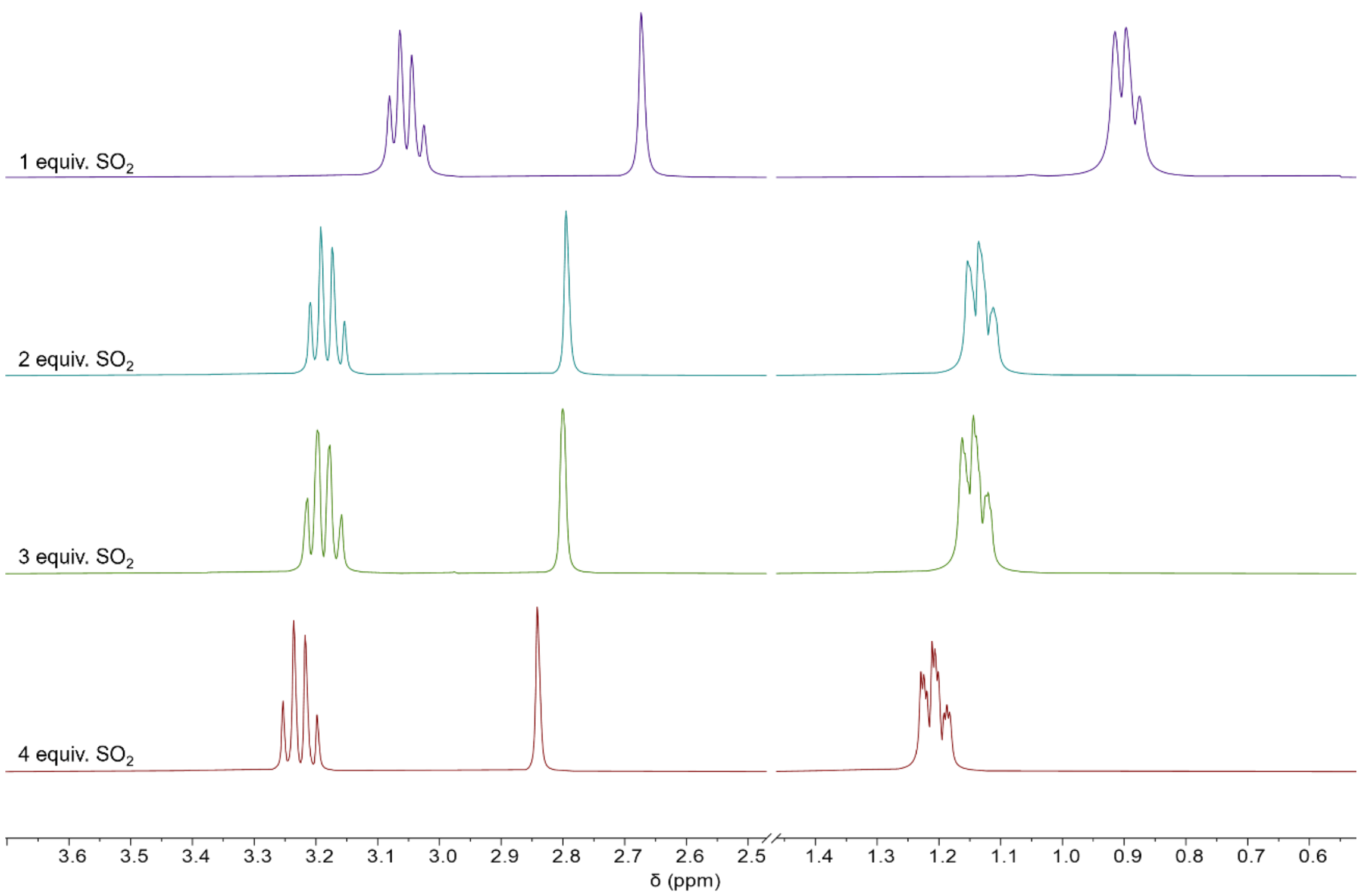


**Figure S11**: $^{1}$H NMR spectra of $[NEt_3Me][Cl(SO_2)]$ (purple), $[NEt_3Me][Cl(SO_2)_2]$ (blue), $[NEt_3Me][Cl(SO_2)_3]$ (green) and $[NEt_3Me][Cl(SO_2)_{3.66}]$ (red) in $CH_2Cl_2$. $CD_2Cl_2$ was used for reference.

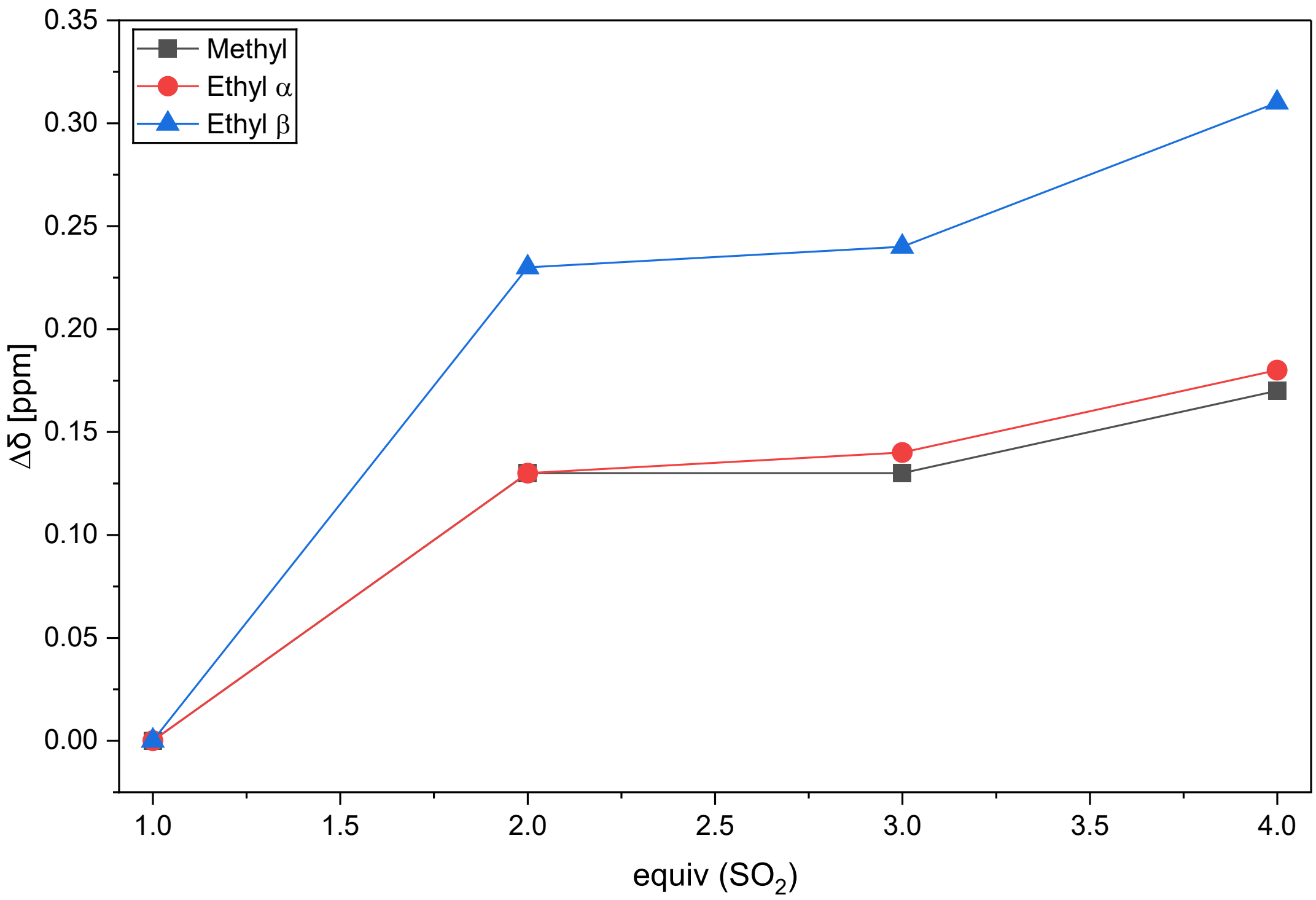

**Figure S12**: Difference in chemical shift of the $^1H$ NMR signals of the IL with different loadings in $CH_2Cl_2$ in comparison to the loading with 1 equiv.

### 2.2.5 Stability Studies

[$NEt_3Me$]Cl (10.2 g, 67.2 mmol) was loaded with $SO_2$ and divided into three batches. Each batch of ionic liquid [$NEt_3Me$][$Cl(SO_2)_{3.66}$] was stored under different environmental conditions: a) at room temperature with (day)light exposure, b) at room temperature under light exclusion and c) at 50 °C under light exclusion. To monitor their long-term stability NMR spectra were recorded on a regular basis (Figures S13-S15). Over a period of more than six months, no decomposition of the ionic liquid was observed under any of the three conditions.

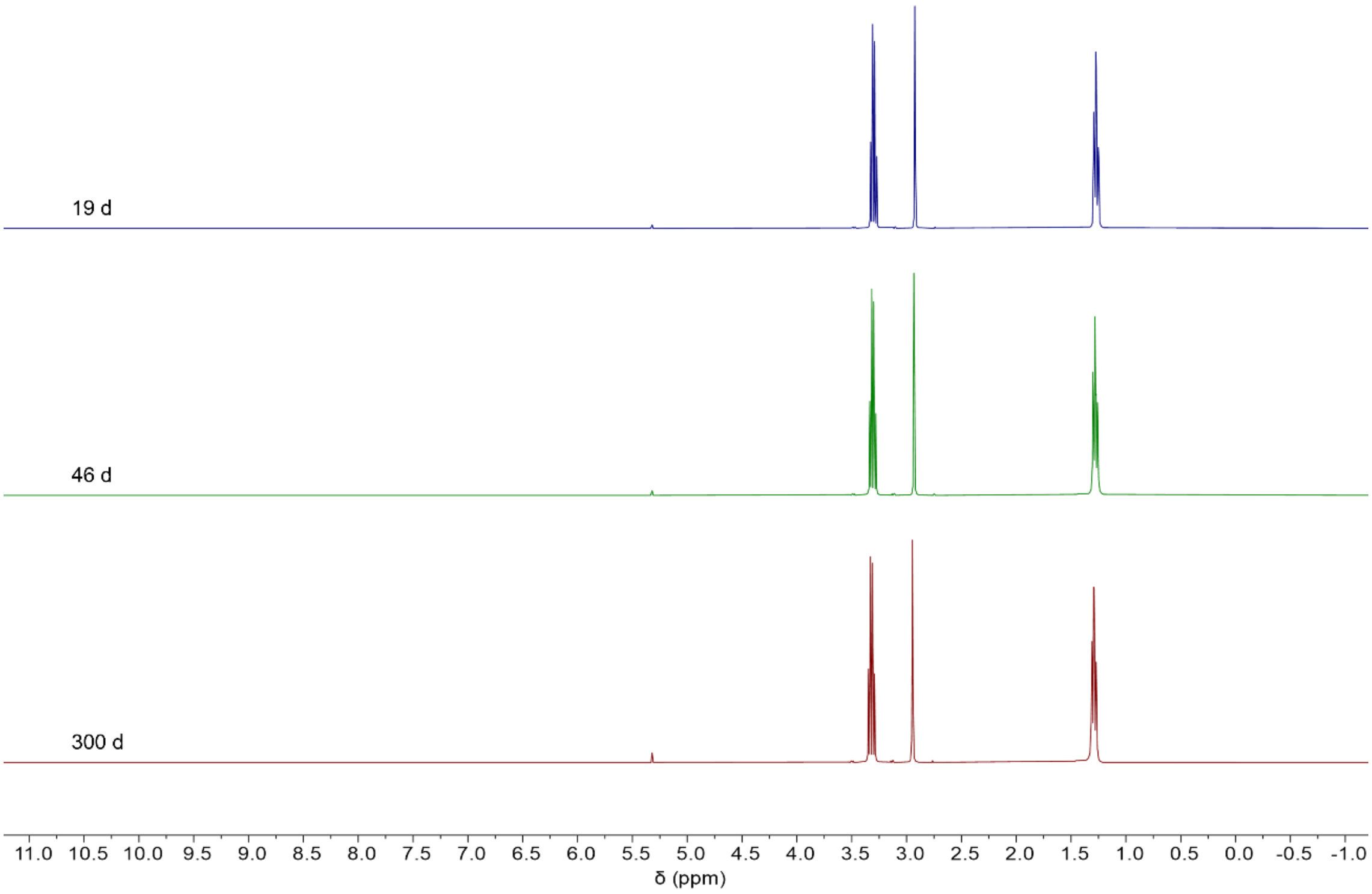


**Figure S13**: Stability $^1H$ NMR studies of the IL under light exposure after 19 days (blue), 46 days (green) and 300 days (red).

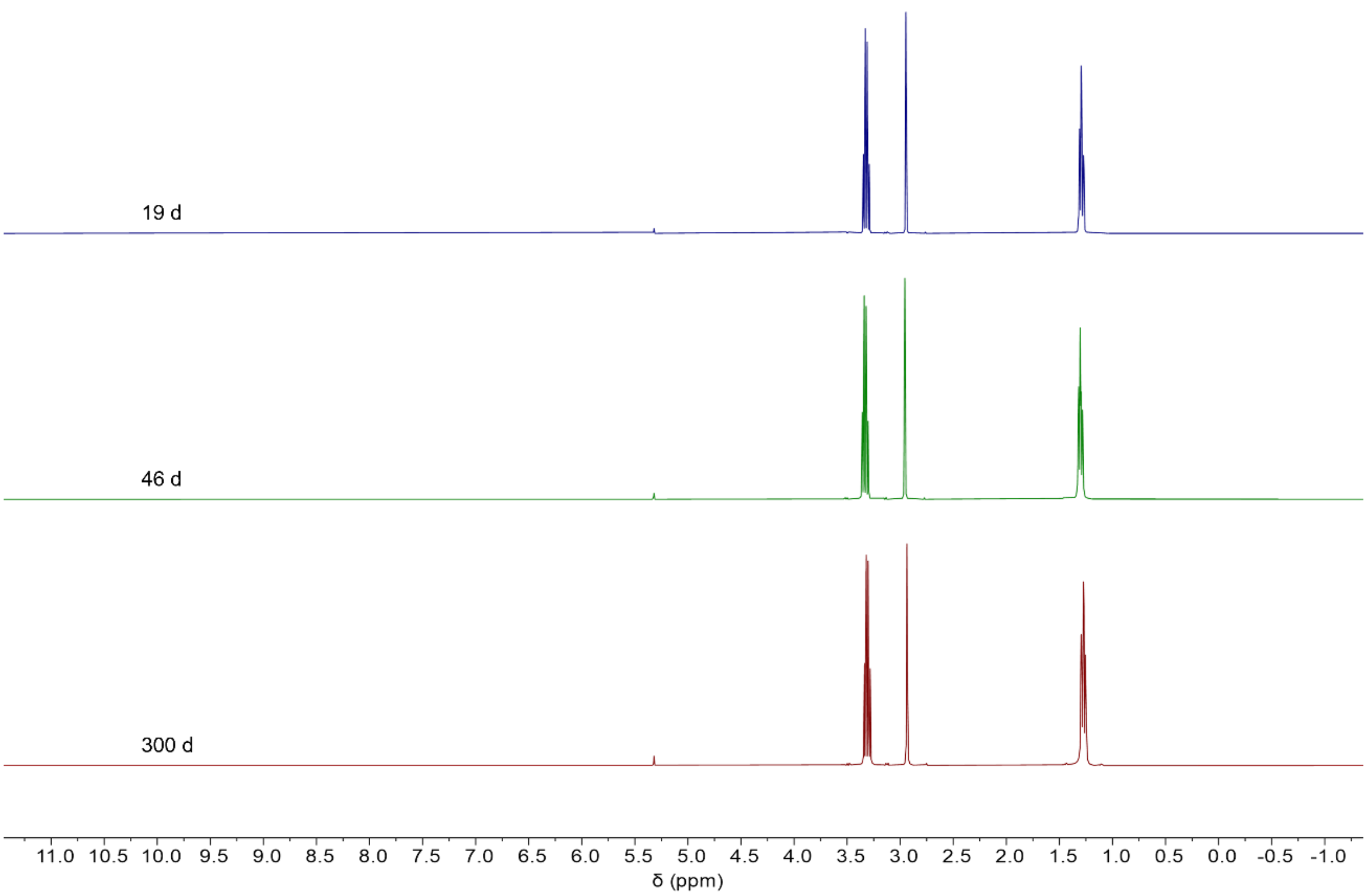


**Figure S14**: Stability $^{1}$H NMR studies of the IL under heat exposure at 50 °C after 19 days (blue), 46 days (green) and 300 days (red).

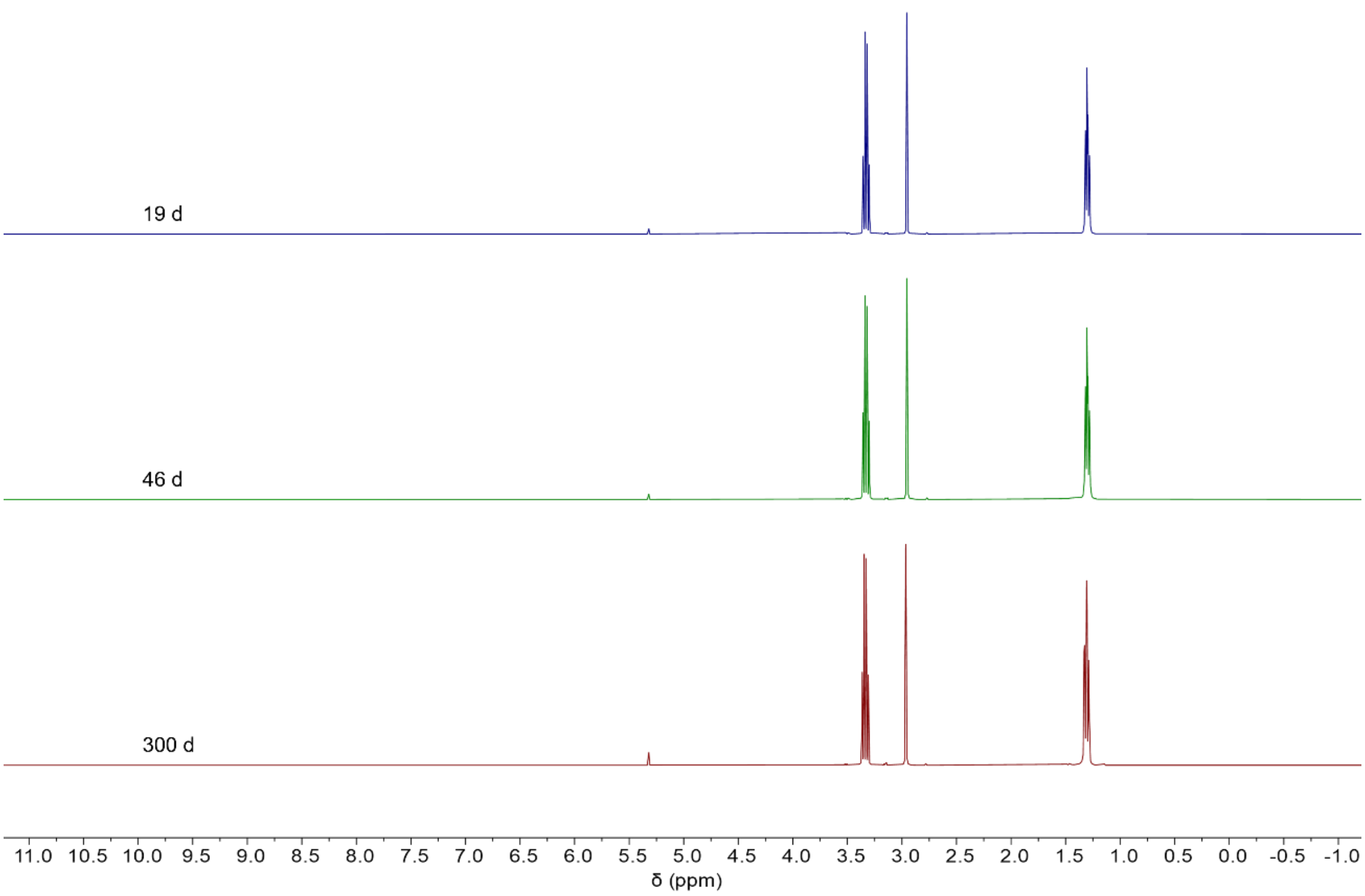

**Figure S15**: Stability $^{1}$H NMR studies of the IL under light exclusion after 19 days (blue), 46 days (green) and 300 days (red).

### 2.2.6 Melting Point

[$NEt_3Me$][$Cl(SO_2)_n$] (1.5 g, 23 mmol) was prepared in an 8 mL Schlenk tube and cooled to −196 °C. The vessel was placed in a glass Dewar filled with a cooling bath of ethanol at −80 °C (Figure S16). The aperture was allowed to slowly warm up until the sample was molten. The temperature was constantly measured. The determination of the melting point was performed three times, and the melting range was determined to be within −52.5 to −51.0 °C.

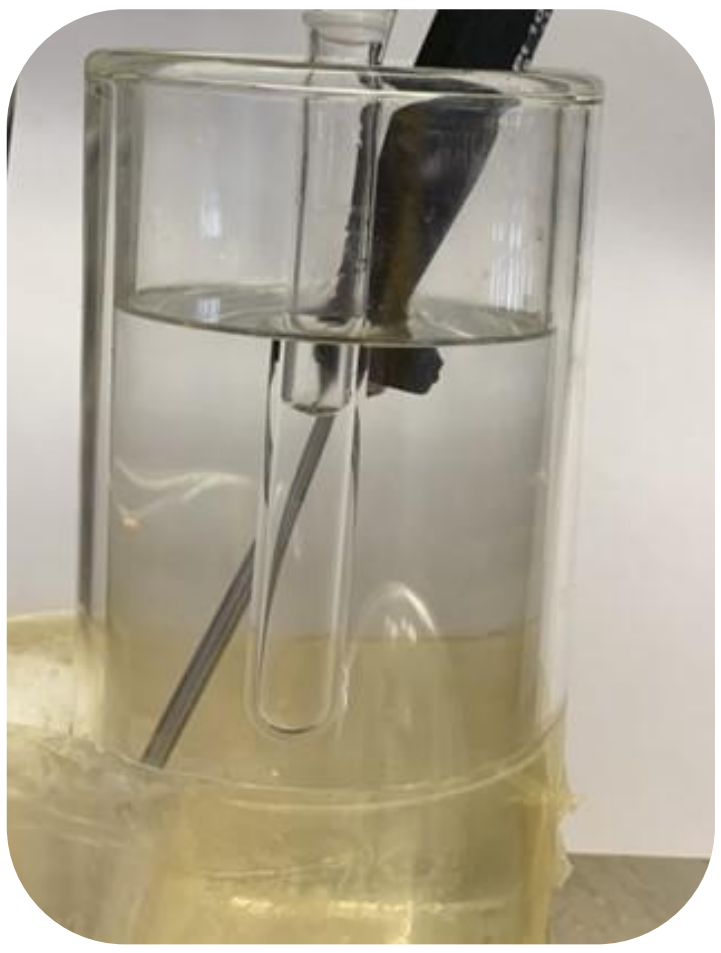

**Figure S16**: Setup for the determination of the melting range.

### 2.2.7 Conductivity

[$NEt_3Me$][$Cl(SO_2)_n$] (80.3 g, 208 mmol) was filled into a temperature controlled Dewar with a connected thermostat. A septum with a conductivity measuring electrode was added and while the ionic liquid was stirred and cooled to −5 °C (Figure S17). The conductivity measurements were carried out at temperature intervals of 1-3 °C and performed without stirring. At 25 °C, the Dewar was replaced with a heating bath. At 30 °C, above the experiment was ended due to a significant gas evolution that was observed.

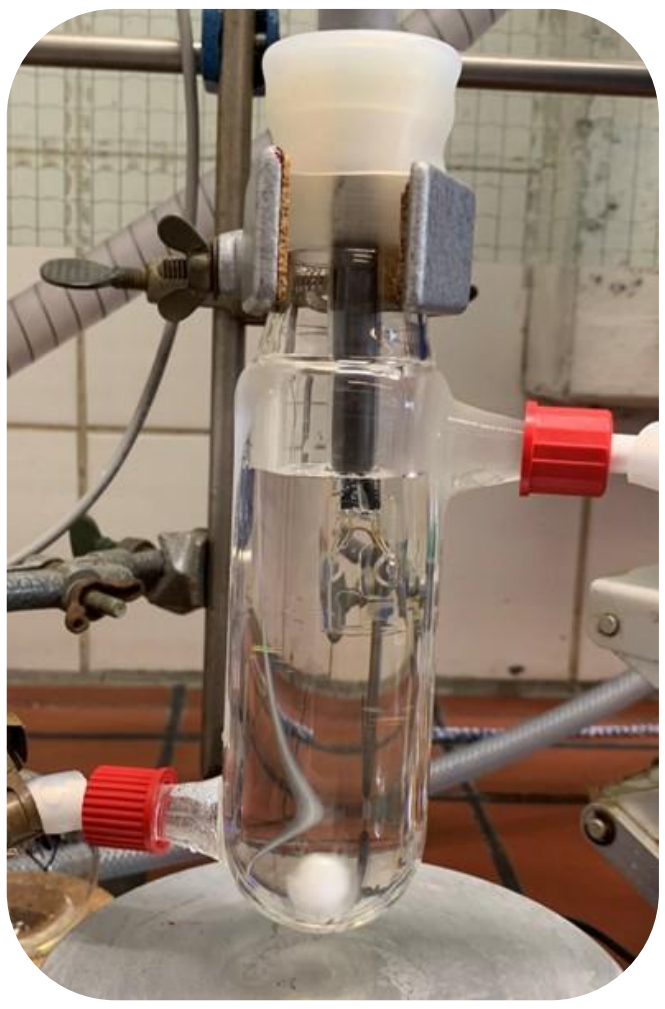

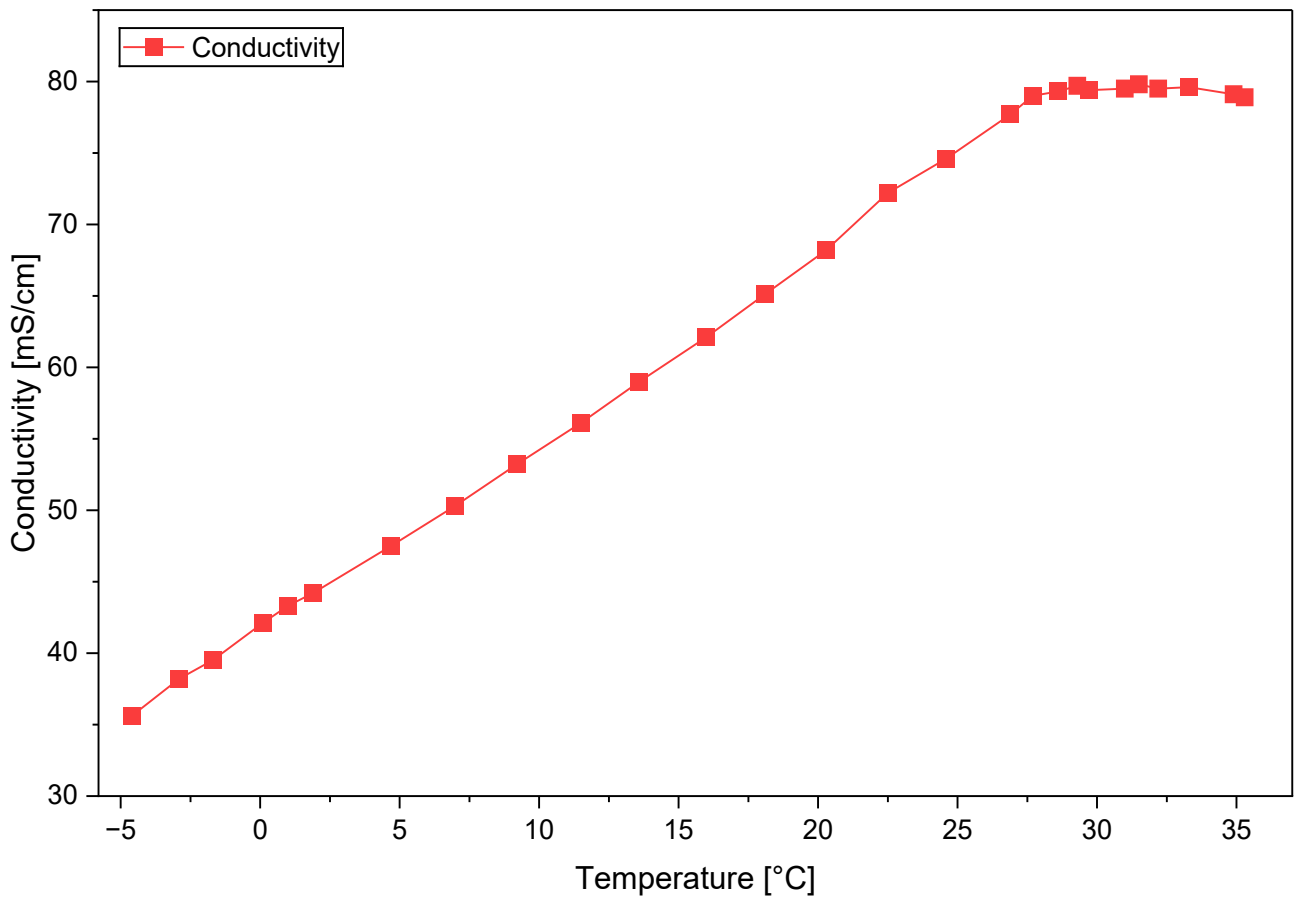



**Figure S17**: Experimental setup for the temperature dependent conductivity measurements (left) and temperature dependent conductivity curve (right).

### 2.2.8 Dynamic Viscosity and Density

For the viscosity measurements, a minimal amount of ionic liquid (~ 3 mL) was poured into an Ubbelohde viscosimeter (Figure S18) at 20 °C. A rubber bulb was used to pull liquid into the smaller arm of the apparatus, with the middle part being sealed off. The time it took for the ionic liquid to travel between two designated marks on the apparatus was measured multiple times to determine the average value. This average value was multiplied with an apparatus specific coefficient ($k = 0.9755\ mm^2/s^2$) to give the kinematic viscosity of the ionic liquid.

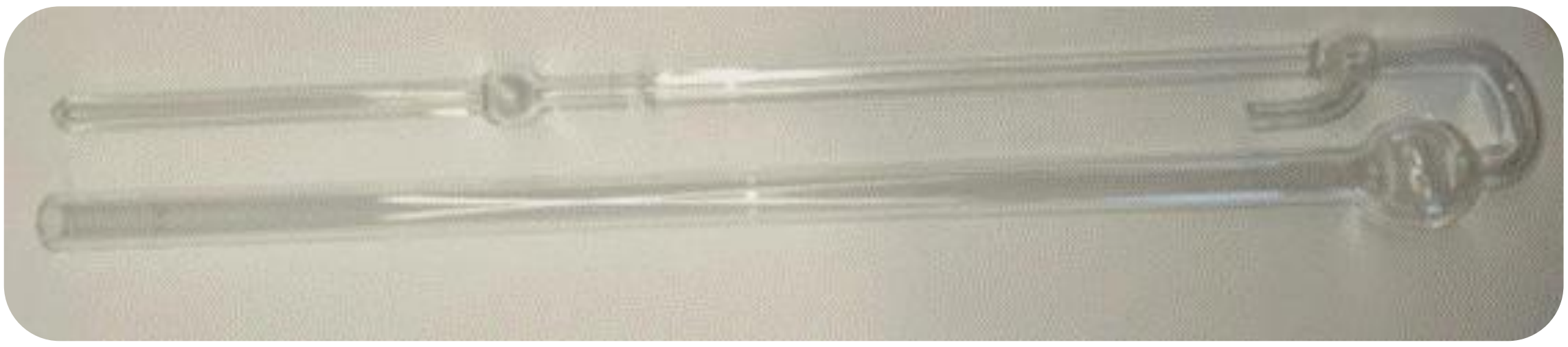

**Figure S18**: Ubbelohde viscosimeter.

The density of the ionic liquid was measured by weighing a defined volume of substance (1 mL). By multiplying the density with the kinematic viscosity, the dynamic viscosity was obtained.

Dynamic viscosity [mPa·s]: 5.70.

Density [g/cm$^3$]: 1.35.

### 2.2.9 Atom Economy and Cost Estimation

Atom economy and approximate costs were calculated for the fully loaded [$NEt_3Me$][$Cl(SO_2)_{3.66}$] and compared to the ones of DABSO, SOgen, $K_2S_2O_5$ and $Na_2S_2O_4$

(Table S2, Table S3). For SOgen *p*-methyl styrene was chosen as a dienophile. For the calculation, the molar mass of $SO_2$ ($M(SO_2)$) was multiplied by the number of $SO_2$ molecules released ($N(SO_2$ released)) and divided by the sum of the molar masses of the reactants involved for $SO_2$ release (M(reactants)). Multiplying by 100 gives the atom economy in percent (Equation S1).

**Equation S1**: Formula for determining the atom economy of $SO_2$ surrogates in percent.

$$atom\ economy = \frac{M(SO_2) * N(SO_2\ released)}{\sum M(reatants)} * 100\%$$

**Table S2**: Values for calculating the atom economy for $[NEt_3Me][Cl(SO_2)_{3.66}]$, DABSO, SOgen, $K_2S_2O_5$ and $Na_2S_2O_4$.

| | $[NEt_3Me][Cl(SO_2)_{3.66}]$ | DABSO | SOgen | $K_2S_2O_5$ | $Na_2S_2O_4$ |
|---|---|---|---|---|---|
| **N ($SO_2$ released)** | 3.66 | 2 | 1 | 2 | 1 |
| **Σ(M(reactants) [g/mol]** | 386.15 | 240.3 | 549.9 | 222.33 | 174.11 |
| **Atom Economy [%]** | 60.7 | 53.3 | 11.7 | 57.6 | 36.8 |

**Table S3**: Cost approximation for $[NEt_3Me][Cl(SO_2)_{3.66}]$, DABSO, SOgen, $K_2S_2O_5$ and $Na_2S_2O_4$.

| | M [g/mol] | Price (€/g)[19–21] |
|---|---|---|
| **$SO_2$** | 64.0648 | 0.01 |
| **DABSO** | 240.30256 | 2.59 |
| **SOgen** | 549.8993 | 10.25 |
| **Tetrabromothiophene S,S-dioxide** | 431.7236 | 4.32 |
| **Dienophile (*p*-methyl-styrene)** | 118.1757 | 5.93 |
| **$[NEt_3Me][Cl(SO_2)_{3.66}]$** | 386.154428 | 0.05 |
| **$[NEt_3Me]Cl$** | 151.67726 | 0.12 |
| **$K_2S_2O_5$** | 222.3256 | 0.22 |
| **$Na_2S_2O_4$** | 174.10914 | 0.10 |

# 3. Quantum Chemical Calculations

## 3.1 Chloride Ion Affinity (CIA)

In a similar fashion to the well-established fluoride ion affinity (FIA), the chloride ion affinity (CIA) was proposed as a measure of Lewis acidity specifically for the chloride ion

as the Lewis base.[22]In this work, the chloride ion affinity was determined using the TMS reference system, since it was already benchmarked with CCSD(T)/CBS for TMSF (FIA), TMSCl (CIA), TMSH (HIA), and TMSMe (MIA) (Table S4).[23–25]

**Table S4**: CCSD(T)/CBS benchmark values in kJ/mol for the trimethylsilylium cation.

| **$[TMS]^+$** | **FIA** | **CIA** | **HIA** | **MIA** |
|---|---|---|---|---|
| **[kJ/mol]** | 952 | 745 | 924 | 981 |

With this system, the difficulty of calculating a naked chloride ion can be circumvented. The CIA of any Lewis acid **A** can now be calculated as the negative of the difference between two enthalpy terms $\Delta H_1$ and $\Delta H_2$, where $\Delta H_2$ has been determined experimentally as 745 kJ/mol (Scheme S1).

$$\mathrm{A} + [\mathrm{Cl}]^- \xrightarrow{-\,\mathrm{CIA}} [\mathrm{ACl}]^- \qquad \mathrm{CIA} = -(\Delta H_1 - \Delta H_2)$$

$$\Delta H_1:\quad \mathrm{A} + \mathrm{TMSCl} \longrightarrow [\mathrm{ACl}]^- + [\mathrm{TMS}]^+$$

$$\Delta H_2:\quad \mathrm{TMSCl} \longrightarrow [\mathrm{Cl}]^- + [\mathrm{TMS}]^+$$

$$\Delta H_2 = 745\ \mathrm{kJ/mol}$$

**Scheme S1**: Definition of chloride ion affinity (CIA) with TMS reference system. **A** represents any Lewis acidic substrate.

Structure optimizations and frequency calculations were performed to verify minima on the potential energy surface. Calculated CIAs are given in table S5, including the ones for $AlCl_3$ and $BCl_3$ that can also be used as anchoring points and are within a deviation of up to 6 kJ/mol, in good agreement with experimental values.[26]

**Table S5**: Calculated chloride ion affinities for **A** ($CIA_A$) at B3LYP-D4/def2-QZVPPD level of density functional theory. Marked entries as references. Enthalpy term **D*H*1** in kJ/ mol for CIA calculation.

| **Substrate A** | **$\Delta H_1$ [kJ/mol]** | **$CIA_A$ [kJ/mol]** |
|---|---|---|
| **$AlCl_3$** | 426.7 | 318.3* |
| **$BCl_3$** | 558.7 | 186.3* |
| **$Cl_2$** | 609.0 | 136.0 |
| **HCl** | 629.8 | 115.2 |
| **$SO_2$** | 622.0 | 123.0 |

### 3.2 Structure Optimization and Vibrational Analysis

Structure Optimizations and vibrational analysis (Table S6) were performed for $[Cl(SO)_n]^-$ with 1-4 equiv $SO_2$.

**Table S6**: Optimized structures of $[Cl(SO_2)_n]^-$ (n = 1-4, top left to bottom right) at the B3LYP-D4/def2-QZVPPD level of density functional theory. Significant bond parameters and vibrations added as reference.

| Structure optimization B3LYP-D4/def2-QZVPPD | |
|---|---|
| 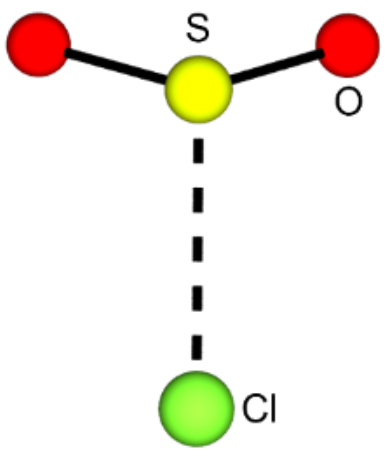 | 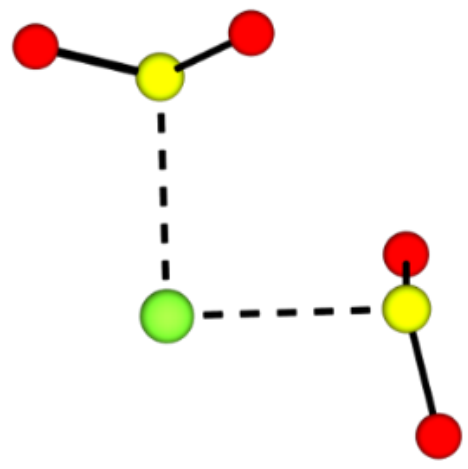 |
| $[Cl(SO_2)]^-$<br>$\bar{\nu}_{sym}$ (S–O) = 1116 $cm^{-1}$<br>r (S–O) = 145.8 pm<br>r (Cl–S) = 253.3 pm | $[Cl(SO_2)_2]^-$<br>$\bar{\nu}_{sym}$ (S–O) = 1135-1141 $cm^{-1}$<br>r (S–O) = 145.0 pm<br>r (Cl–S) = 269.9-270.2 pm |
| 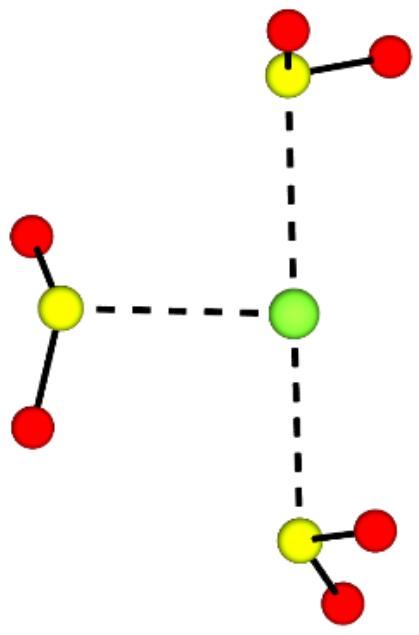 | 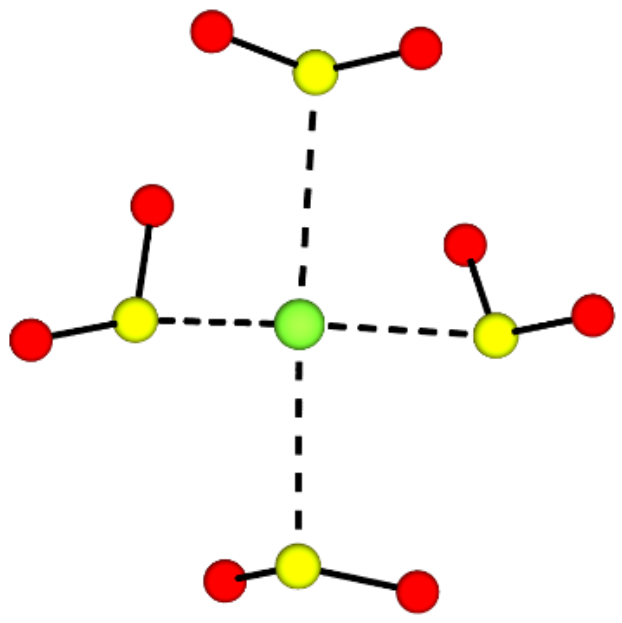 |
| $[Cl(SO_2)_3]^-$<br>$\bar{\nu}_{sym}$ (S–O) = 1145-1157 $cm^{-1}$<br>r (S–O) = 144.4-144.7 pm<br>r (Cl–S[1]) = 275.3-284.5 pm | $[Cl(SO_2)_4]^-$<br>$\bar{\nu}_{sym}$ (S–O) = 1150-1163 $cm^{-1}$<br>r (S–O) = 144.1-144.5 pm<br>r (Cl–S) = 284.1-298.8 pm |

### 3.3 Natural Bond Orbital Analysis (NBO)

Calculated NBOs for $[Cl(SO_2)]^-$ and $SO_2$ were plotted with ChemCraft 1.8 (Figure S19). The interacting orbitals were determined by evaluation of the NBO analysis. The interaction between the chlorine lone pair and the $\pi^*$ orbital from the S−O bond forms the foundation of the charge transfer bond in the chlorosulfite anion. This interaction corresponds to 192 kJ/mol according to second-order perturbation theory of the Fock matix in NBO basis analysis.

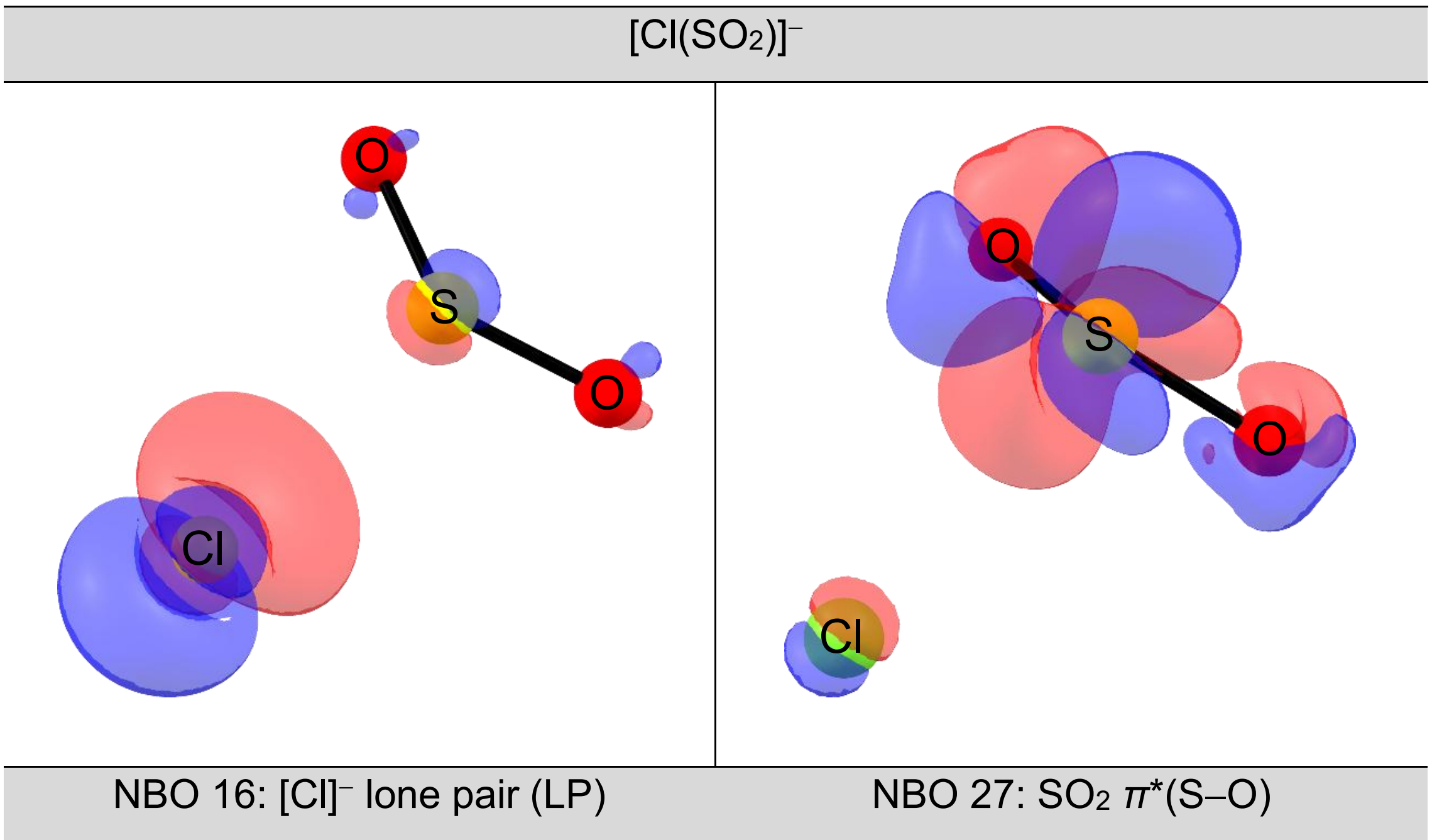


**Figure S19**: Plotted natural bonding orbitals for the $[Cl(SO_2)]^-$ anion. Red and blue isosurfaces represent positive and negative orbital lobes (Isovalue = 0.4). The interacting orbitals are the lone pair of chloride (NBO 16) and the $\pi^*$-Orbital of S−O (NBO 27).

## 3.4 Electrostatic Potential Maps (ESP)

Coulombic interaction-sites were predicted by calculating and visualizing the electrostatic potential map for $SO_2$ and $[Cl(SO_2)]^-$ (Figure S20).

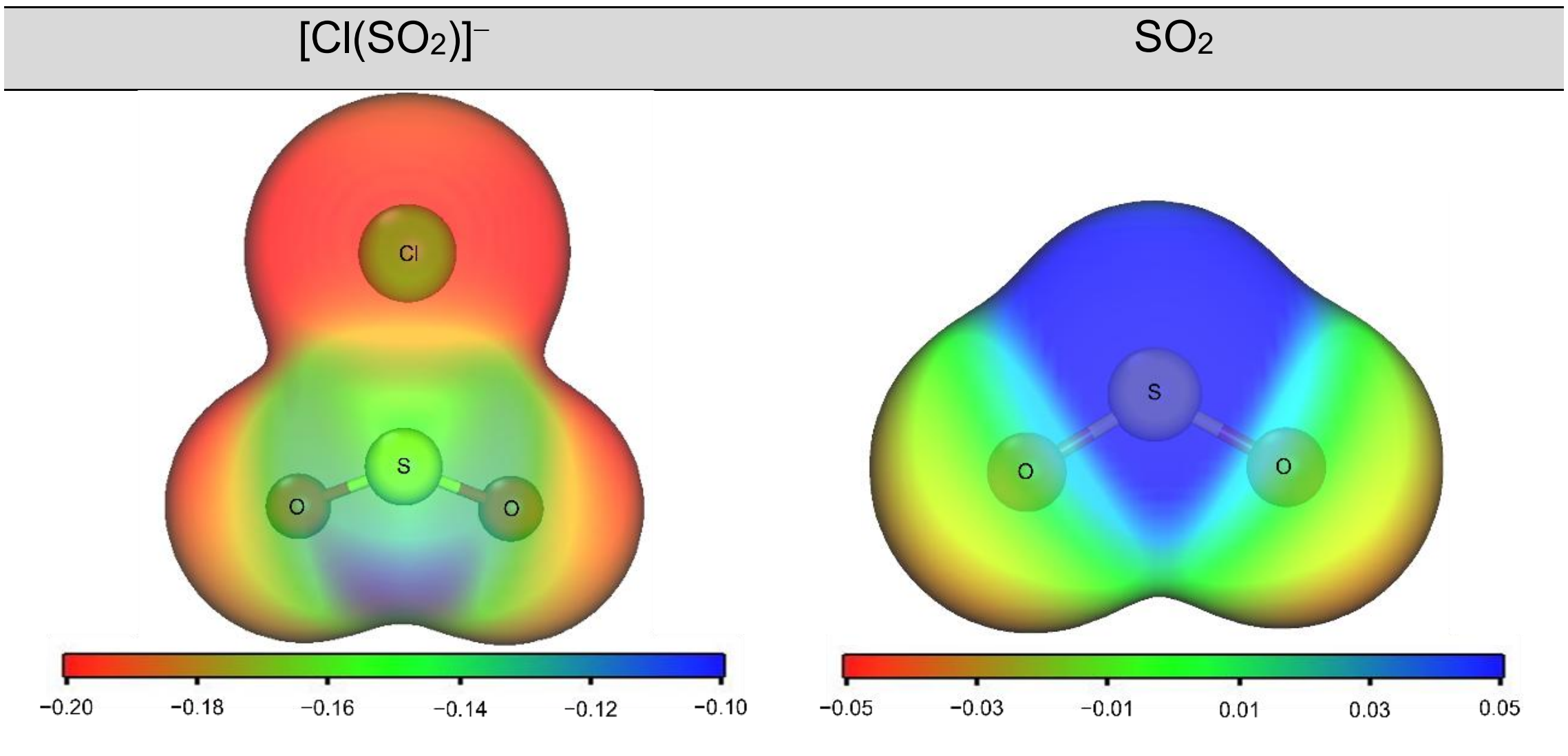


**Figure S20**: Electrostatic potential maps of sulfur dioxide $SO_2$ (right) and the chlorosulfite anion $[Cl(SO_2)]^-$ (left). Range for $[Cl(SO_2)]^-$ from −0.2 a.u (red) to −0.1 a.u (blue). Range for $SO_2$ from −0.05 a.u (red) to 0.05 a.u (blue). Isosurface value for both cases set at 0.0035 a.u. Calculated at B3LYP-D3BJ/def2-QZVPP level of DFT in gas phase.

## 3.5 Quantum Theory of Atoms in Molecules (QTAIM)

QTAIM was performed for $SO_2$ and $[Cl(SO_2)]^-$ and the contour maps are shown in figure S21. For $[Cl(SO_2)]^-$ the Cl−S−O molecular plane is depicted and due to symmetry the second sulfur-oxygen bond with its bond critical point (BCP 3) is omitted. Properties of the bond critical points are listed in table S7.

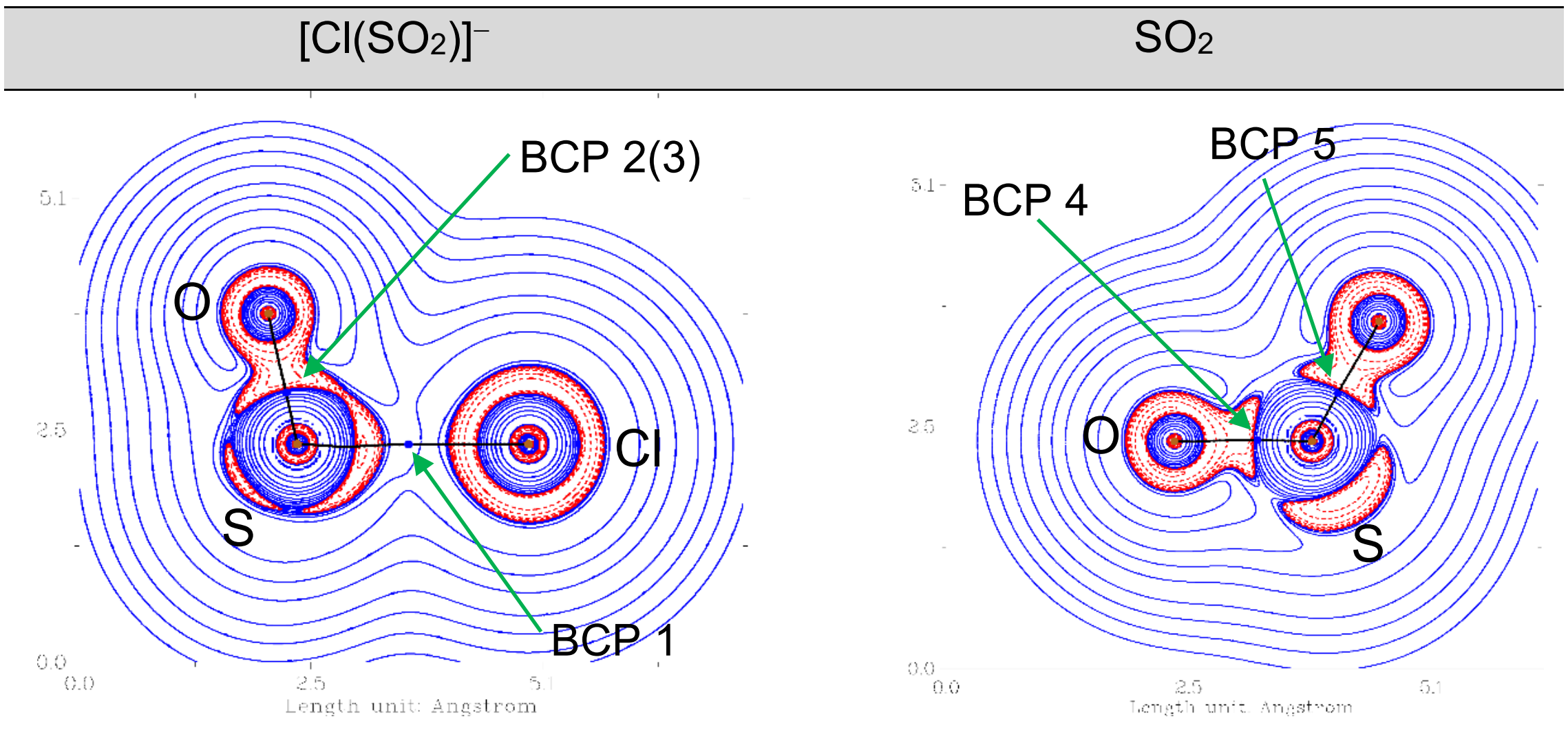


**Figure S21**: Contour maps of the $[Cl(SO_2)]^-$ anion along Cl−S−O plane and (left) $SO_2$ at the molecular plane (right). For $[Cl(SO_2]_n]^-$ the Cl−S−O plane is depicted with the second S−O bond omitted due to symmetry. Blue and red contour lines represent positive and negative isovalues, respectively. Black lines indicate bond paths of electron density between atoms with bond critical points (BCP 1-5, blue dots) at the maxima, highlighted with green arrows.

**Table S7**: Properties of bond critical points (BCP) from calculated electron density for $[Cl(SO_2)]^-$ and $SO_2$. $\rho$: Electron density at bond critical point; $\nabla^2\rho$: Laplacian of electron density at BCP; ELF: Electron localization function; $V$(r): Potential energy density at BCP; $G$(r): Lagrangian kinetic energy; $\varepsilon$: Ellipticity of electron density.

| **Molecule** | **BCP Nr.** | **Bond** | $\rho$ [eA$^{-3}$] | $\nabla^2\rho$ [eA$^{-5}$] | **ELF** | $V$(r) | $G$(r) | $\|V\|/G$ | $\varepsilon$ |
|---|---|---|---|---|---|---|---|---|---|
| $[Cl(SO_2)]^-$ | BCP 1 | S–Cl | 0.385 | 1.70 | 0.44 | –0.04 | 0.03 | 1.36 | 0.065 |
| | BCP 2 | S–O | 2.031 | 19.28 | 0.31 | –0.96 | 0.58 | 1.66 | 0.119 |
| | BCP 3 | S–O | 2.031 | 19.26 | 0.31 | –0.96 | 0.58 | 1.66 | 0.119 |
| $SO_2$ | BCP 4 | S–O | 2.117 | 25.35 | 0.29 | –1.05 | 0.66 | 1.60 | 0.135 |
| | BCP 5 | S–O | 2.116 | 25.31 | 0.29 | –1.05 | 0.66 | 1.60 | 0.135 |

# 4. $SO_2$ Transfer Reactions

## 4.1 3,4-Dimethyl-2,5-dihydrothiophene 1,1-dioxide (2)

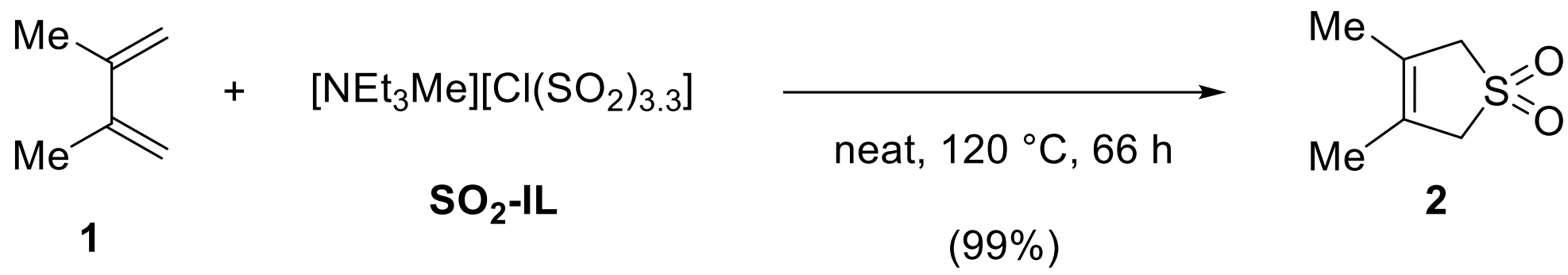


Dihydrothiophene **2** was synthesized according to a modified literature procedure.[27] A 30 mL Rettberg tube was charged with 2,3-dimethyl-1,3-butadiene (**1**) (60.0 µL, 500 µmol, 1.00 equiv) and $[NEt_3Me][Cl(SO_2)_{3.3}]$ (240 µL, 910 µmol, 1.82 equiv) under argon atmosphere. Upon addition, the mixture was heated at 120 °C for 66 h. After cooling to RT, EtOAc (10 mL) was added, and the mixture was filtered. The filtrate was concentrated under reduced pressure, and the crude product was purified by flash column chromatography on silica gel (50% MTBE in petroleum ether) to yield the title compound **2** (72.2 mg, 494 µmol, 99%) as a white, crystalline solid. The analytical data obtained were in full agreement with those reported in the literature.[27]

**TLC** (50% MTBE in petroleum ether): $R_f$ = 0.30 ($KMnO_4$).

**$^1$H NMR** (500 MHz, $CDCl_3$): δ [ppm] = 3.71 (s, 4H), 1.77 (s, 6H).

**$^{13}$C NMR** (126 MHz, $CDCl_3$): δ [ppm] = 125.7, 60.8, 14.7.

<u>*Comparison to the literature (Ref. 28):*</u> Yield of **2** employing DABSO = 80%.

## 4.2 Temperature and $SO_2$ Loading Dependence

**Table S8**: Influence of temperature and $SO_2$ loading.

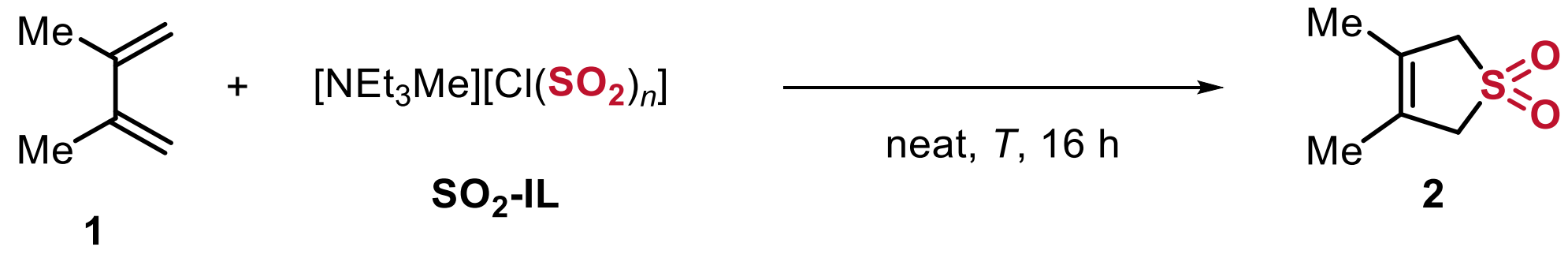


| Entry | $SO_2$-IL [equiv] | *n* | *T* [°C] | $^1$H NMR Yield [%][a] |
|---|---|---|---|---|
| **1** | 1.8 | 3.3 | 120 | 99 |
| **2** | 1.8 | 3.3 | 80 | 89 |
| **3** | 1.8 | 3.3 | 50 | 91 |
| **4** | 1.8 | 3.3 | 20 | 47 |
| **5** | 6.0 | 1.0 | 120 | 99 |

[a] Determined using $CH_2Br_2$ as internal standard.

*Conclusion:* While the reaction remains highly efficient at higher $SO_2$ loading ($n$ = 3.3, Entries 1–3) at 50 °C, product formation decreases at room temperature (Entry 4). Nevertheless, the 3-sulfolene **2** could still be obtained in 47% NMR yield. To our delight, $SO_2$ transfer can also be achieved at 120 °C when the ammonium chloride is loaded with only one equivalent of $SO_2$ ($n$ = 1.0, Entry 5). These observations are consistent with the vapor pressure curves discussed in the main text (Figure 2A).

## 4.3 Decagram-Scale Synthesis of 2

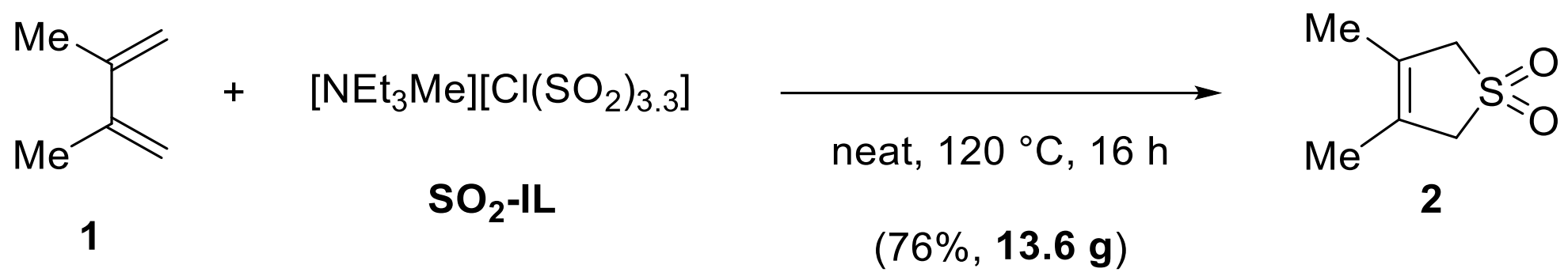


A mixture of 2,3-dimethyl-1,3-butadiene (**1**) (13.7 mL, 122 mmol, 1.00 equiv) and [$NEt_3Me$][$Cl(SO_2)_{3.0}$] (42.0 g, 31.1 mL, 122 mmol, 1.00 equiv) placed in a 350 mL pressure tube (purchased from FengTecEx) was heated to 120 °C. After 16 h, the reaction mixture was allowed to cool to RT (Figure S22A) and sat. aq. NaCl solution (250 mL) and EtOAc (400 mL) were added. The layers were separated, the aq. layer was extracted with EtOAc (3 × 250 mL), the combined organic layers were dried over $MgSO_4$, filtered, and concentrated under reduced pressure (Figure S22B). Purification of the crude product by recrystallisation from refluxing $H_2O$ (100 mL), followed by trituration with *n*-pentane (2 × 200 mL) at 0 °C afforded the title compound **2** (13.6 g, 92.9 mmol, 76%) as an off-white, crystalline solid (Figure S22C). The analytical data obtained were in full agreement with those reported in the literature.[27]

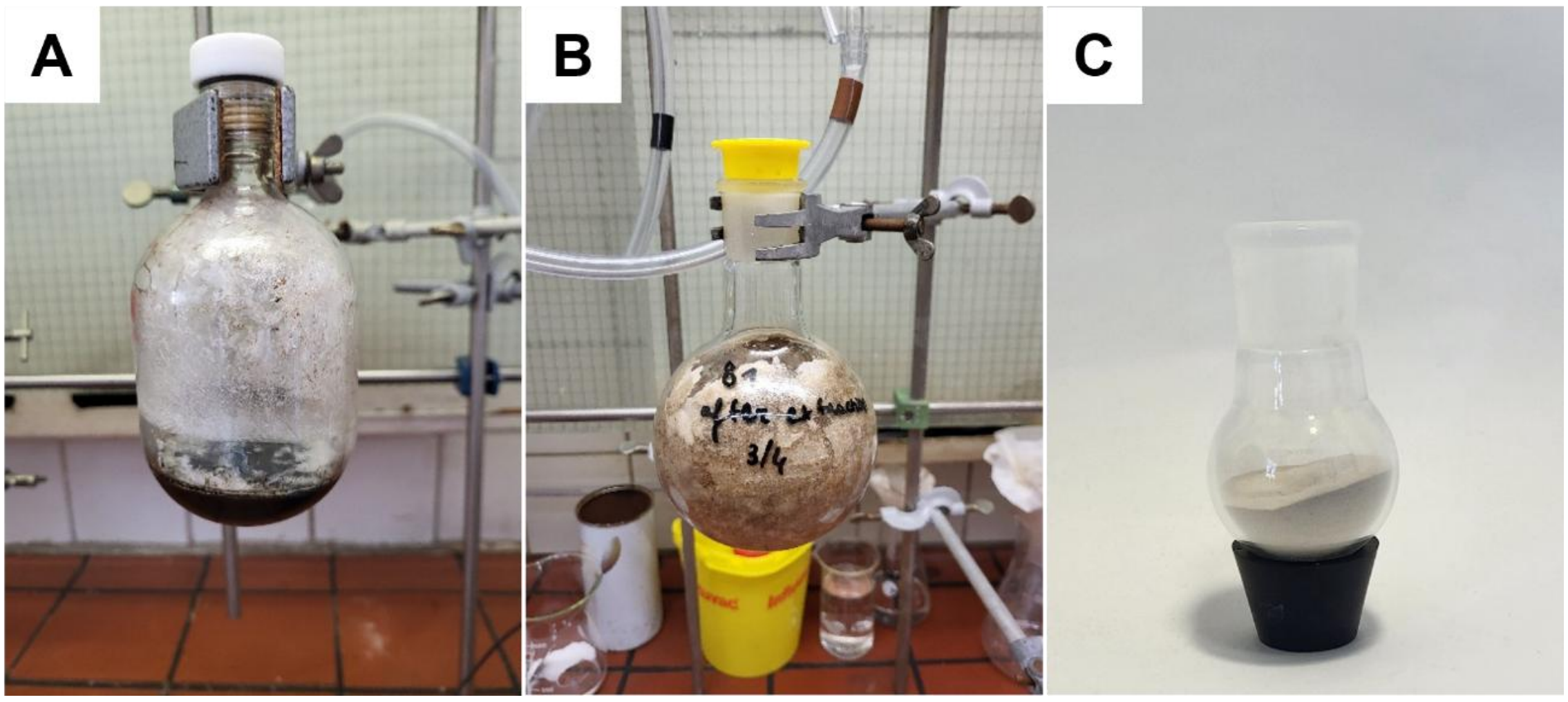


**Figure S22:** Graphical guide for the decagram-scale synthesis of 3-sulfolene **2**. A) Reaction mixture after 16 h. B) Crude product after extraction. C) Product after recrystallization and trituration.

## 4.4 4-(Phenylsulfonyl)morpholine (3)

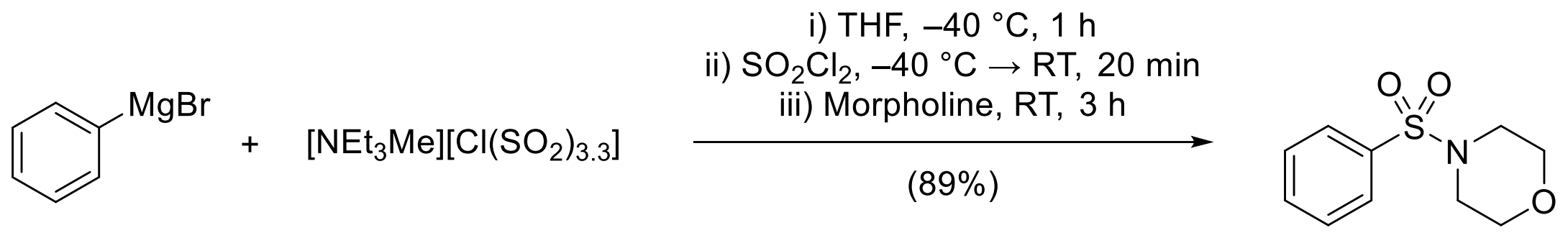


**S7** **$SO_2$-IL** **3**

The title compound **3** was synthesized according to a modified literature-known procedure.[27] To a 50 mL Schlenk flask charged with $[NEt_3Me][Cl(SO_2)_{3.3}]$ (410 µL, 1.52 mmol, 1.52 equiv), THF (12 mL, 0.08 M) was added at RT and the suspension was cooled to –40 °C. Phenylmagnesium bromide solution (**S7**, 1.00 mL, 1.00 mmol, 1.00 equiv, 1.00 M in THF) was then added dropwise and upon addition, the reaction mixture was kept stirring at –40 °C. After one hour, sulfuryl chloride (100 µL, 1.20 mmol, 1.20 equiv) was added dropwise at –40 °C. After five minutes, the reaction mixture was allowed to warm to RT. After 20 minutes, morpholine (870 µL, 10.0 mmol, 10.0 equiv) was added dropwise at RT. After three hours, $H_2O$ (20 mL) and $CHCl_3$ (20 mL) were added and the layers were separated. The aq. layer was extracted with $CHCl_3$ (2 × 20 mL), the combined organic layers were dried over $MgSO_4$, filtered, and concentrated under reduced pressure. Purification of the crude product by flash column chromatography on silica gel (33% → 50% MTBE in petroleum ether) afforded the title compound **3** (202 mg, 890 µmol, 89%) as a white, crystalline solid. The analytical data obtained were in full agreement with those reported in the literature.[27]

**TLC** (50% MTBE in petroleum ether): $R_f$ = 0.32 (UV, $KMnO_4$).

**$^1$H NMR** (400 MHz, $CDCl_3$): δ [ppm] = 7.75 (d, $^3J$ = 7.1 Hz, 2H), 7.66 – 7.60 (m, 1H), 7.58 – 7.53 (m, 2H), 3.74 (t, $^3J$ = 4.7 Hz, 4H), 2.99 (t, $^3J$ = 4.7 Hz, 4H).

**$^{13}$C NMR** (126 MHz, $CDCl_3$): δ [ppm] = 135.2, 133.2, 129.3, 128.0, 66.2, 46.1.

<u>*Comparison to the literature (Ref. 28):*</u> Yield of **3** employing DABSO = 74%.

## 4.5 *Tert*-butyl 2-((4-acetylphenyl)sulfonyl)acetate (4)

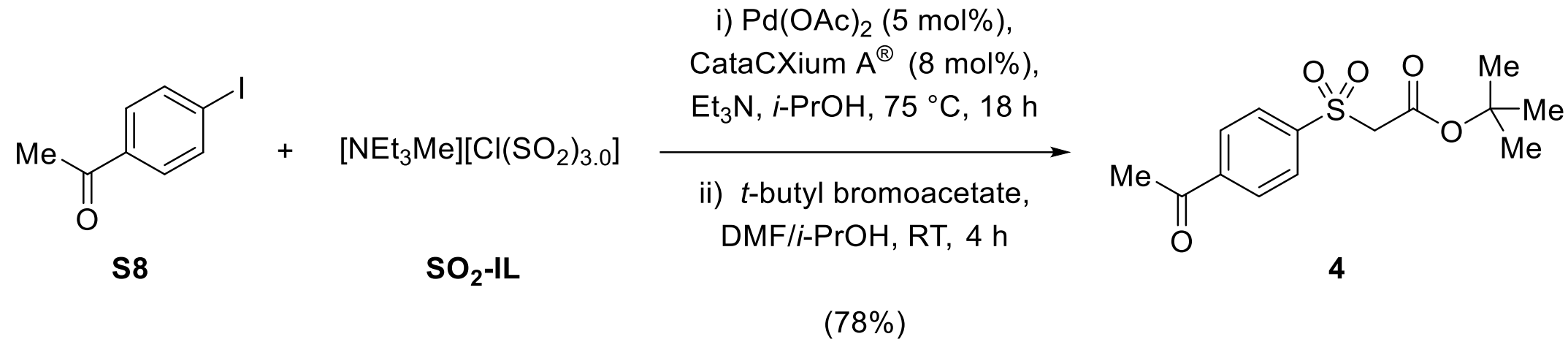


Sulfone **3** was synthesized according to a modified literature procedure.[28] A 15 mL Rettberg tube was charged with *iso*-propyl alcohol (2.5 mL, 0.20 M) and the solvent was degassed by performing freeze, pump, thaw technique three times. Then, 4'-iodoacetophenone (**S8**, 123 mg, 500 µmol, 1.00 equiv) was added, followed by $Pd(OAc)_2$ (5.61 mg, 25.0 µmol, 5.00 mol%), CataCXium A® (14.3 mg, 40.0 µmol, 8.00 mol%), $NEt_3$ (210 µL, 1.50 mmol, 3.00 equiv) and $[NEt_3Me][Cl(SO_2)_{3.0}]$ (50.0 µL, 0.20 mmol, 0.400 equiv) under argon counterflow at RT. Upon addition, the reaction mixture was heated to 75 °C and stirred overnight. After 18 h, the mixture was allowed to cool to RT and a solution of *t*-butyl bromoacetate (150 µL, 1.00 mmol, 2.00 equiv) in DMF (0.83 mL, 0.60 M) was added dropwise. The reaction mixture was then stirred at RT for 3.5 h. It was poured onto distilled $H_2O$ (40 mL) and extracted with EtOAc (3 × 40 mL). The combined organic layers were dried over $MgSO_4$, filtered, and concentrated under reduced pressure. Purification of the crude product by flash column chromatography on silica gel (10% → 50% MTBE in petroleum ether) afforded the title compound **4** (116 mg, 389 µmol, 78%) as a white, crystalline solid. The analytical data obtained were in full agreement with those reported in the literature.[28]

**TLC** (25% MTBE in petroleum ether): $R_f$ = 0.20 (UV, dark red on *p*-anisaldehyde).

**$^1$H NMR** (500 MHz, $CDCl_3$): δ [ppm] = 8.13 (d, $^3J$ = 8.5 Hz, 2H), 8.04 (d, $^3J$ = 8.6 Hz, 2H), 4.06 (s, 2H), 2.67 (s, 3H), 1.37 (s, 9H).

**$^{13}$C NMR** (126 MHz, $CDCl_3$): δ [ppm] = 196.6, 161.2, 142.7, 141.2, 129.1, 129.0, 84.2, 62.0, 27.8, 27.1.

<u>*Comparison to the literature (Ref. 29):*</u> Yield of **4** employing DABSO = 74%.

## 4.6 Synthesis of *N,N′*-Diphenylsulfamide (5)

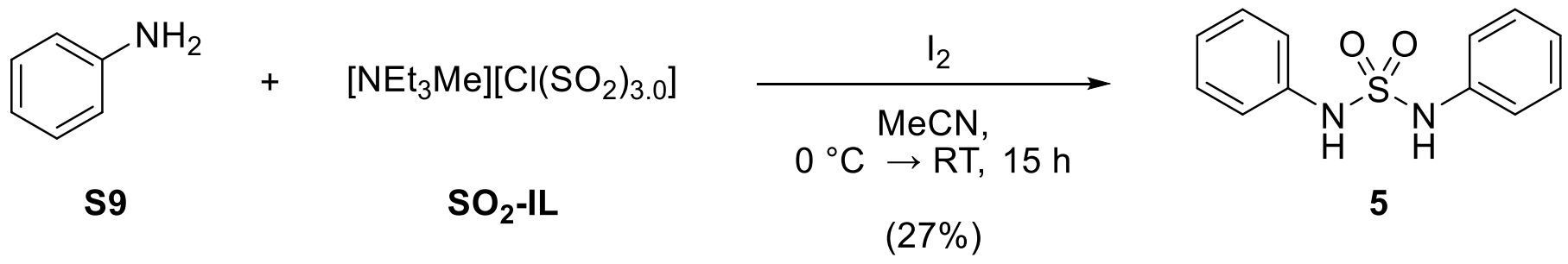


Sulfamide **5** was synthesized according to a modified literature procedure.[27] A 50 mL Schlenk flask was charged with a suspension of $[NEt_3Me][Cl(SO_2)_{3.0}]$ (340 µL, 1.33 mmol, 1.33 equiv) in MeCN (9.5 mL, 0.12 M): After cooling to 0 °C, iodine (381 mg, 1.50 mmol, 1.50 equiv) was added to the mixture and stirred for 25 min resulting in a homogenous solution. Subsequently, aniline (**S9**, 90.0 µL, 1.00 mmol, 1.00 equiv) was added dropwise at 0 °C. Upon addition, the reaction mixture was slowly allowed to warm to RT. After 15 h, the reaction mixture was quenched with aq. NaOH solution (10%, 40 mL), followed by careful acidification by the addition of citric acid (pH value = 6). The aq. mixture was extracted with $CH_2Cl_2$ (3 × 10 mL) and the combined organic layers were successively washed with aq. HCl solution (1 M, 10 mL) and aq. sat. NaCl solution (10 mL), dried over $MgSO_4$, filtered, and concentrated under reduced pressure. Purification of the crude product by flash column chromatography on silica gel (25% → 33% MTBE in petroleum ether) afforded the title compound **5** (34.0 mg, 137 µmol, 27%) as a light-yellow solid. The analytical data obtained were in full agreement with those reported in the literature.[27]

**TLC** (33% MTBE in petroleum ether): $R_f$ = 0.43 (UV, $KMnO_4$).

**$^1$H NMR** (400 MHz, $CDCl_3$): δ [ppm] = 7.28 (t, $^3J$ = 7.9 Hz, 4H), 7.14 (t, $^3J$ = 7.3 Hz, 2H), 7.08 (d, $^3J$ = 7.5 Hz, 4H), 6.92 (s, 2H).

**$^{13}$C NMR** (126 MHz, $CDCl_3$): δ [ppm] = 136.4, 129.6, 125.5, 121.3.

<u>*Comparison to the literature (Ref. 28):*</u> Yield of **5** employing DABSO = 63%.

## 4.7 Synthesis of Phenylmethanesulfonyl Fluoride (6)

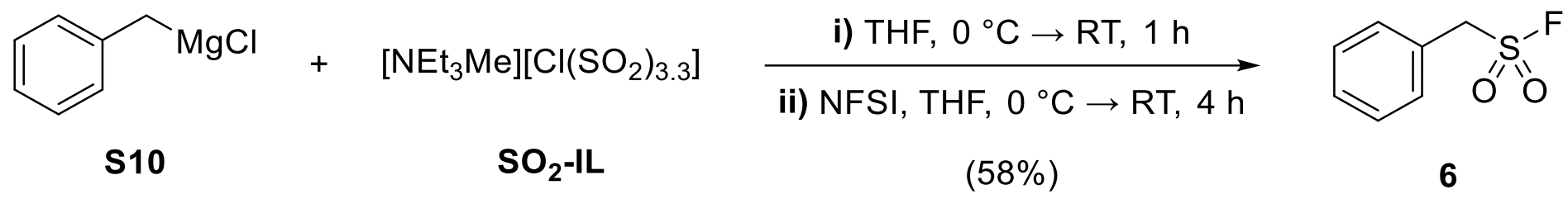


Sulfonyl fluoride **6** was synthesized according to a modified literature procedure.[29] A 25 mL Schlenk flask was charged with a suspension of $[NEt_3Me][Cl(SO_2)_{3.0}]$ (90.0 µL, 350 µmol, 0.350 equiv) in THF (2.0 mL, 0.50 M) and cooled to 0 °C. Then, benzylmagnesium chloride solution (**S10**, 1.00 M in $Et_2O$, 1.00 mL, 1.00 mmol, 1.00 equiv) was added dropwise and the reaction mixture was allowed to warm to RT and stirred for 1 h. Then, NFSI (473 mg, 1.50 mmol, 1.50 equiv) was added portion wise at 0 °C and the mixture stirred at RT for 4 h. The reaction mixture was quenched with aq. sat. $NH_4Cl$ solution (25 mL) and EtOAc (10 mL) was added. The layers were separated and the aq. layer was extracted with EtOAc (3 × 20 mL). The combined organic layers were dried over $MgSO_4$, filtered, and concentrated under reduced pressure. Purification of the crude product by flash column chromatography on silica gel (2% → 5% MTBE in petroleum ether) yielded the title compound **6** (101 mg, 580 µmol, 58%) as a white, crystalline solid. The analytical data obtained were in full agreement with those reported in the literature.[29]

**TLC** (10% MTBE in petroleum ether): $R_f$ = 0.41 ($KMnO_4$).

**$^1$H NMR** (500 MHz, $CDCl_3$): δ [ppm] = 7.47 – 7.42 (m, 5H), 4.60 (d, $^3J$ = 3.2 Hz, 2H).

**$^{19}$F NMR** (377 MHz, $CDCl_3$): δ [ppm] = 51.4 (t, $^3J$ = 3.3 Hz).

**$^{13}$C NMR** (126 MHz, $CDCl_3$): δ [ppm] = 130.8, 130.1, 129.5, 125.6, 57.0 (d, $^2J$ = 17.6 Hz).

<u>*Comparison to the literature (Ref. 30):*</u> Yield of **6** employing DABSO = 64%.

# 5. Appendix

## 5.1 Crystallographic Data

**Table S9**: Crystal data and structure refinement for [$NEt_3Me$][$Cl(SO_2)$].

| | |
|---|---|
| **CCDC number** | **2528652** |
| **Empirical formula** | $C_7H_{18}ClNO_2S$ |
| **Formula weight** | 215.73 |
| **Temperature [K]** | 100(2) |
| **Crystal system** | monoclinic |
| **Space group (number)** | $P2_1/n$ (14) |
| ***a* [Å]** | 8.5764(5) |
| ***b* [Å]** | 12.4846(6) |
| ***c* [Å]** | 10.2607(5) |
| **α [°]** | 90 |
| **β [°]** | 92.637(2) |
| **γ [°]** | 90 |
| **Volume [Å³]** | 1097.48(10) |
| ***Z*** | 4 |
| $\rho_{calc}$ **[gcm⁻³]** | 1.306 |
| $\mu$ **[mm⁻¹]** | 0.505 |
| ***F*(000)** | 464 |
| **Crystal size [mm³]** | 0.218×0.313×0.352 |
| **Crystal color** | colorless |
| **Crystal shape** | prism |
| **Radiation** | Mo$K_\alpha$ (λ=0.71073 Å) |
| **2θ range [°]** | 5.14 to 56.58 (0.75 Å) |
| **Index ranges** | −11 ≤ h ≤ 11<br>−16 ≤ k ≤ 16<br>−13 ≤ l ≤ 13 |
| **Reflections collected** | 34979 |
| **Independent reflections** | 2721<br>$R_{int}$ = 0.0579<br>$R_{sigma}$ = 0.0226 |
| **Completeness to θ = 25.242°** | 99.8% |
| **Data / Restraints / Parameters** | 2721 / 0 / 113 |
| **Absorption correction $T_{min}/T_{max}$ (method)** | 0.6087 / 0.7457 (multi-scan) |
| **Goodness-of-fit on $F^2$** | 1.068 |

| | |
|---|---|
| **Final *R* indexes [*I*≥2σ(*I*)]** | $R_1$ = 0.0252<br>w$R_2$ = 0.0585 |
| **Final *R* indexes [all data]** | $R_1$ = 0.0305<br>w$R_2$ = 0.0615 |
| **Largest peak/hole [eÅ$^{-3}$]** | 0.29/−0.29 |

**Table S10**: Crystal data and structure refinement for [PNP][Cl($SO_2$)]•$CH_2Cl_2$.

| | |
|---|---|
| **CCDC Number** | **2528646** |
| **Empirical formula** | $C_{37}H_{32}Cl_3NO_2P_2S$ |
| **Formula weight** | 722.98 |
| **Temperature/K** | 100.00 |
| **Crystal system** | triclinic |
| **Space group** | P-1 |
| **a/Å** | 9.6187(13) |
| **b/Å** | 10.5641(17) |
| **c/Å** | 16.860(3) |
| **α/°** | 89.345(5) |
| **β/°** | 86.494(5) |
| **γ/°** | 86.441(4) |
| **Volume/Å$^3$** | 1706.7(5) |
| **Z** | 2 |
| **$\rho_{calc}$g/cm$^3$** | 1.407 |
| **μ/mm$^{-1}$** | 0.459 |
| **F(000)** | 748.0 |
| **Crystal size/mm$^3$** | 0.134 × 0.128 × 0.048 |
| **Radiation** | MoKα (λ = 0.71073) |
| **2Θ range for data collection/°** | 4.25 to 50.984 |
| **Index ranges** | −11 ≤ h ≤ 11<br>−12 ≤ k ≤ 12<br>−15 ≤ l ≤ 20 |
| **Reflections collected** | 14258 |
| **Independent reflections** | 6062<br>$R_{int}$ = 0.0978<br>$R_{sigma}$ = 0.1261 |
| **Data/restraints/parameters** | 6062/0/415 |
| **Goodness-of-fit on F$^2$** | 1.013 |
| **Final R indexes [I>=2σ (I)]** | $R_1$ = 0.0580<br>w$R_2$ = 0.1082 |

| | |
|---|---|
| **Final R indexes [all data]** | $R_1$ = 0.1232<br>$wR_2$ = 0.1404 |
| **Largest diff. peak/hole / e Å$^{-3}$** | 0.54/−0.60 |

**Table S11**: Crystal data and structure refinement for $[PPh_4][Cl(SO_2)_2]$.

| | |
|---|---|
| **Identification code** | **2528647** |
| **Empirical formula** | $C_{24}H_{20}ClO_4PS_2$ |
| **Formula weight** | 502.94 |
| **Temperature/K** | 100.00 |
| **Crystal system** | monoclinic |
| **Space group** | $P2_1/n$ |
| **a/Å** | 16.0103(15) |
| **b/Å** | 7.4217(6) |
| **c/Å** | 20.8130(17) |
| **α/°** | 90 |
| **β/°** | 109.886(3) |
| **γ/°** | 90 |
| **Volume/Å$^3$** | 2325.6(3) |
| **Z** | 4 |
| **$\rho_{calc}$g/cm$^3$** | 1.436 |
| **μ/mm$^{-1}$** | 0.442 |
| **F(000)** | 1040.0 |
| **Crystal size/mm$^3$** | 0.294 × 0.151 × 0.102 |
| **Radiation** | MoKα (λ = 0.71073) |
| **2Θ range for data collection/°** | 4.162 to 50.7 |
| **Index ranges** | −19 ≤ h ≤ 18<br>−8 ≤ k ≤ 8<br>−25 ≤ l ≤ 25 |
| **Reflections collected** | 21433 |
| **Independent reflections** | 4251<br>$R_{int}$ = 0.0513<br>$R_{sigma}$ = 0.0383 |
| **Data/restraints/parameters** | 4251/0/289 |
| **Goodness-of-fit on F$^2$** | 1.057 |
| **Final R indexes [I>=2σ (I)]** | $R_1$ = 0.0413<br>$wR_2$ = 0.0943 |
| **Final R indexes [all data]** | $R_1$ = 0.0540<br>$wR_2$ = 0.1064 |

| | |
|---|---|
| **Largest diff. peak/hole / e Å$^{-3}$** | 0.32/−0.40 |

**Table S12**: Crystal data and structure refinement for $[NEt_4][Cl(SO_2)_{3.5}]_2$.

| | |
|---|---|
| **CCDC number** | **2528651** |
| **Empirical formula** | $C_8H_{20}ClNO_7S_{3.5}$ |
| **Formula weight** | 389.91 |
| **Temperature/K** | 99.99 |
| **Crystal system** | monoclinic |
| **Space group** | C2/c |
| **a/Å** | 26.5670(14) |
| **b/Å** | 7.6379(4) |
| **c/Å** | 17.0444(9) |
| **α/°** | 90 |
| **β/°** | 95.996(2) |
| **γ/°** | 90 |
| **Volume/Å$^3$** | 3439.7(3) |
| **Z** | 4 |
| **$\rho_{calc}$g/cm$^3$** | 1.506 |
| **μ/mm$^{-1}$** | 0.673 |
| **F(000)** | 1632.0 |
| **Crystal size/mm$^3$** | 0.649 × 0.305 × 0.257 |
| **Radiation** | MoKα (λ = 0.71073) |
| **2Θ range for data collection/°** | 4.806 to 56.556 |
| **Index ranges** | −35 ≤ h ≤ 35<br>−10 ≤ k ≤ 10<br>−22 ≤ l ≤ 22 |
| **Reflections collected** | 60572 |
| **Independent reflections** | 4274<br>$R_{int}$ = 0.0277<br>$R_{sigma}$ = 0.0120 |
| **Data/restraints/parameters** | 4274/18/208 |
| **Goodness-of-fit on F$^2$** | 1.242 |
| **Final R indexes [I>=2σ (I)]** | $R_1$ = 0.0555<br>$wR_2$ = 0.1175 |
| **Final R indexes [all data]** | $R_1$ = 0.0560<br>$wR_2$ = 0.1177 |
| **Largest diff. peak/hole / e Å$^{-3}$** | 1.06/−0.83 |

## 5.2 NMR Spectra

[NEt$_3$Me][Cl(SO$_2$)$_n$]

$^1$H NMR (400 MHz, CD$_2$Cl$_2$)

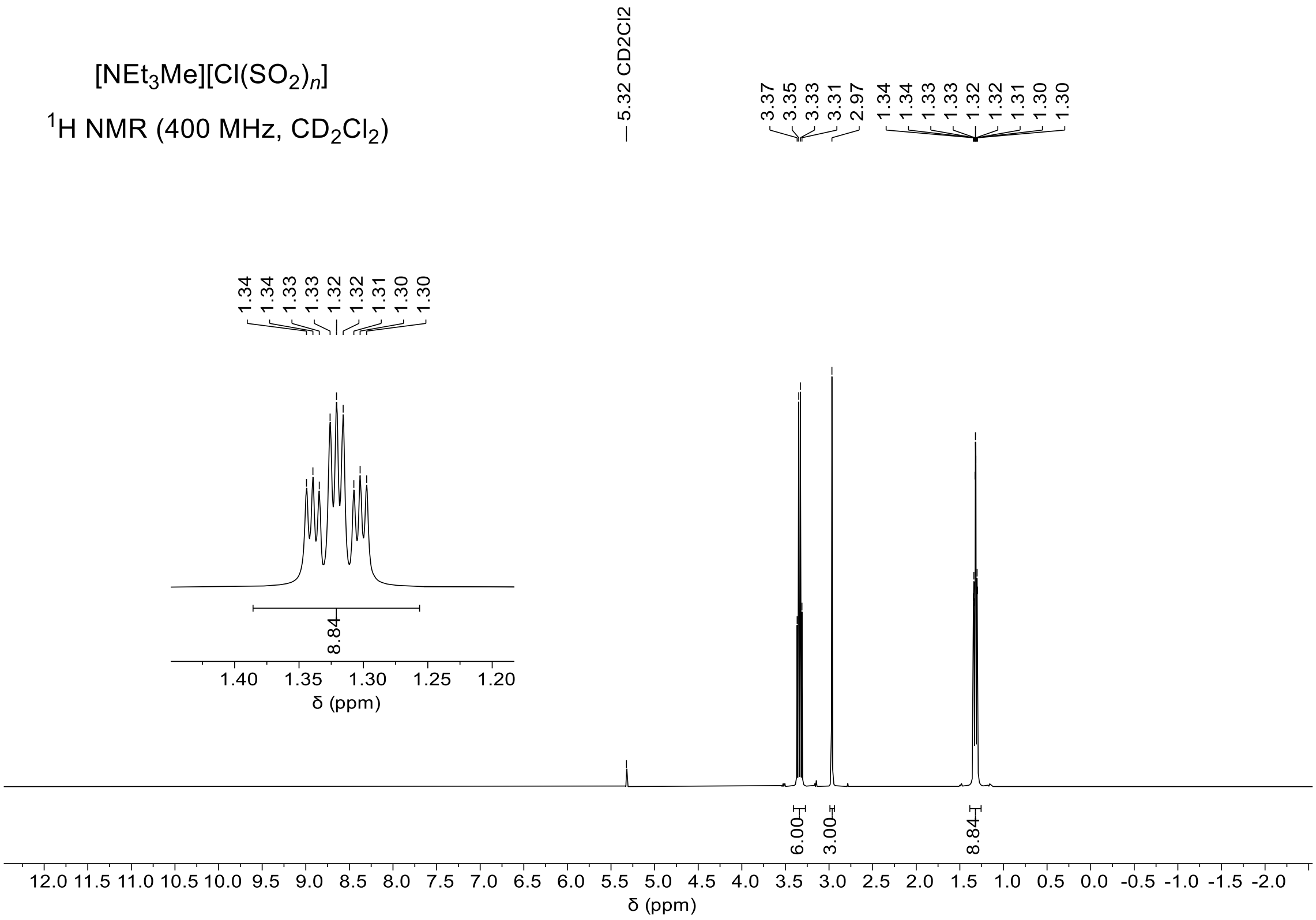


[NEt$_3$Me][Cl(SO$_2$)$_n$]

$^{13}$C NMR (101 MHz, CD$_2$Cl$_2$)

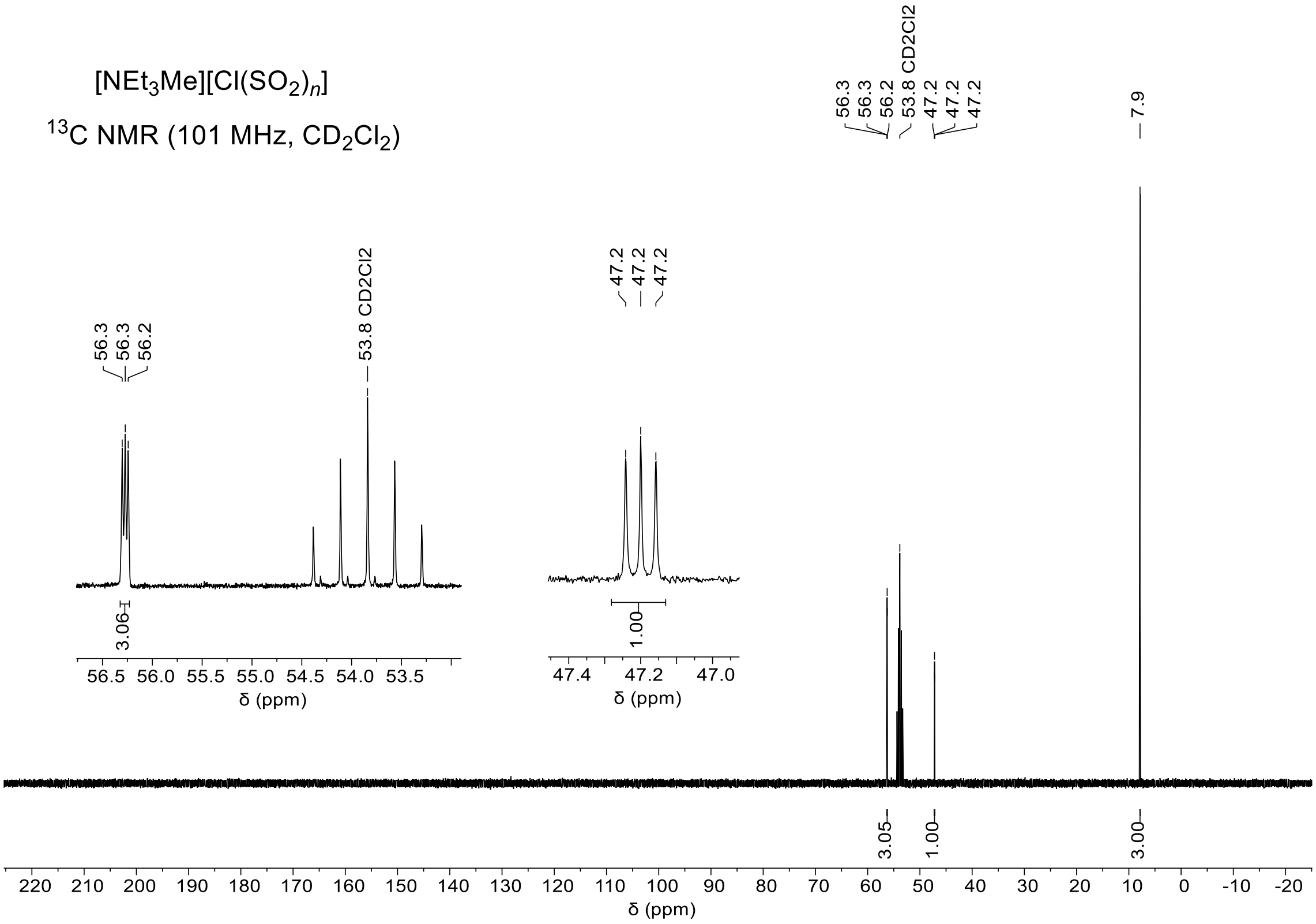

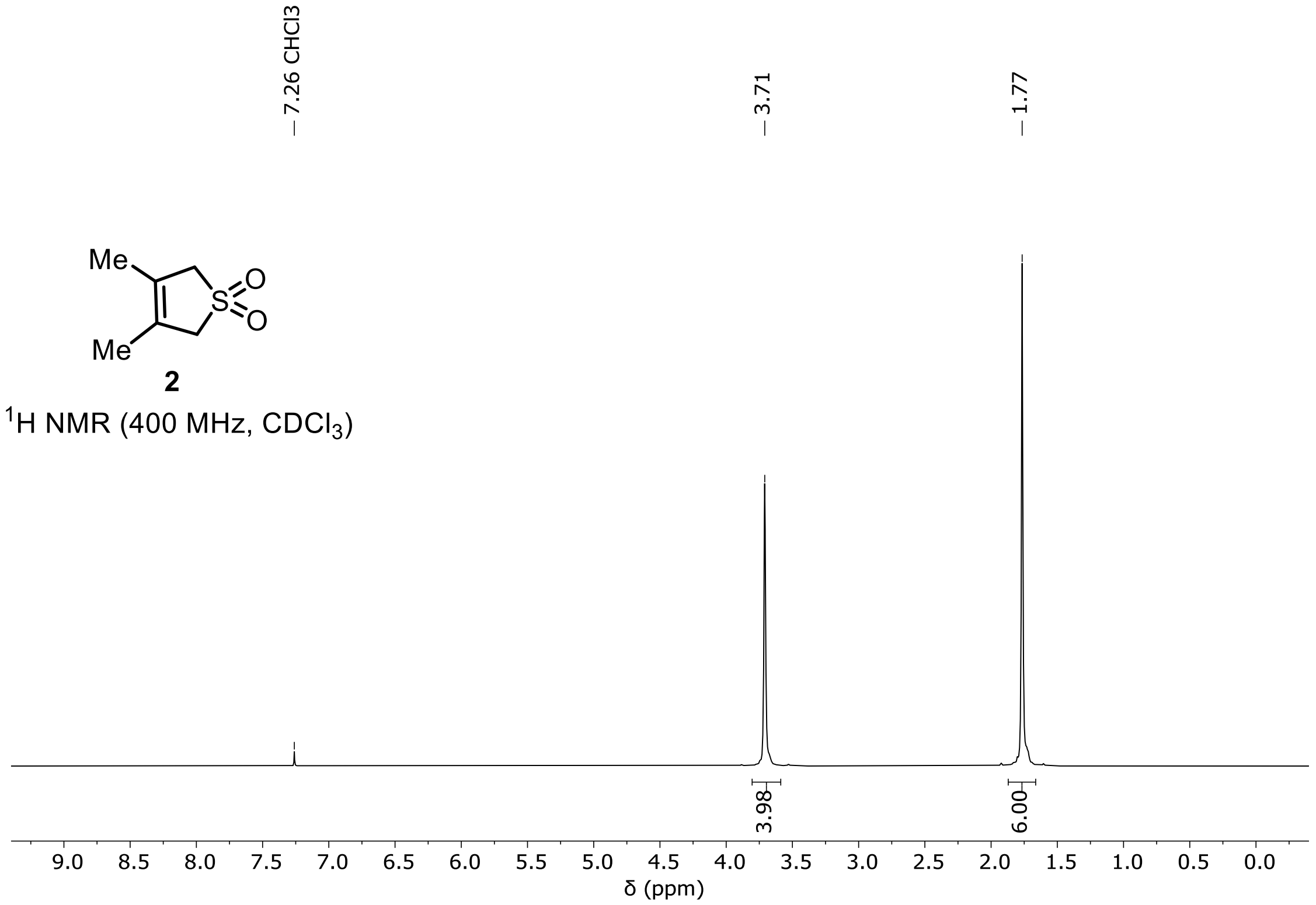
7.26 CHCl3
3.71
1.77
Me
Me
S
O
O
2
$^{1}$H NMR (400 MHz, $CDCl_3$)
3.98
6.00
9.0 8.5 8.0 7.5 7.0 6.5 6.0 5.5 5.0 4.5 4.0 3.5 3.0 2.5 2.0 1.5 1.0 0.5 0.0
δ (ppm)

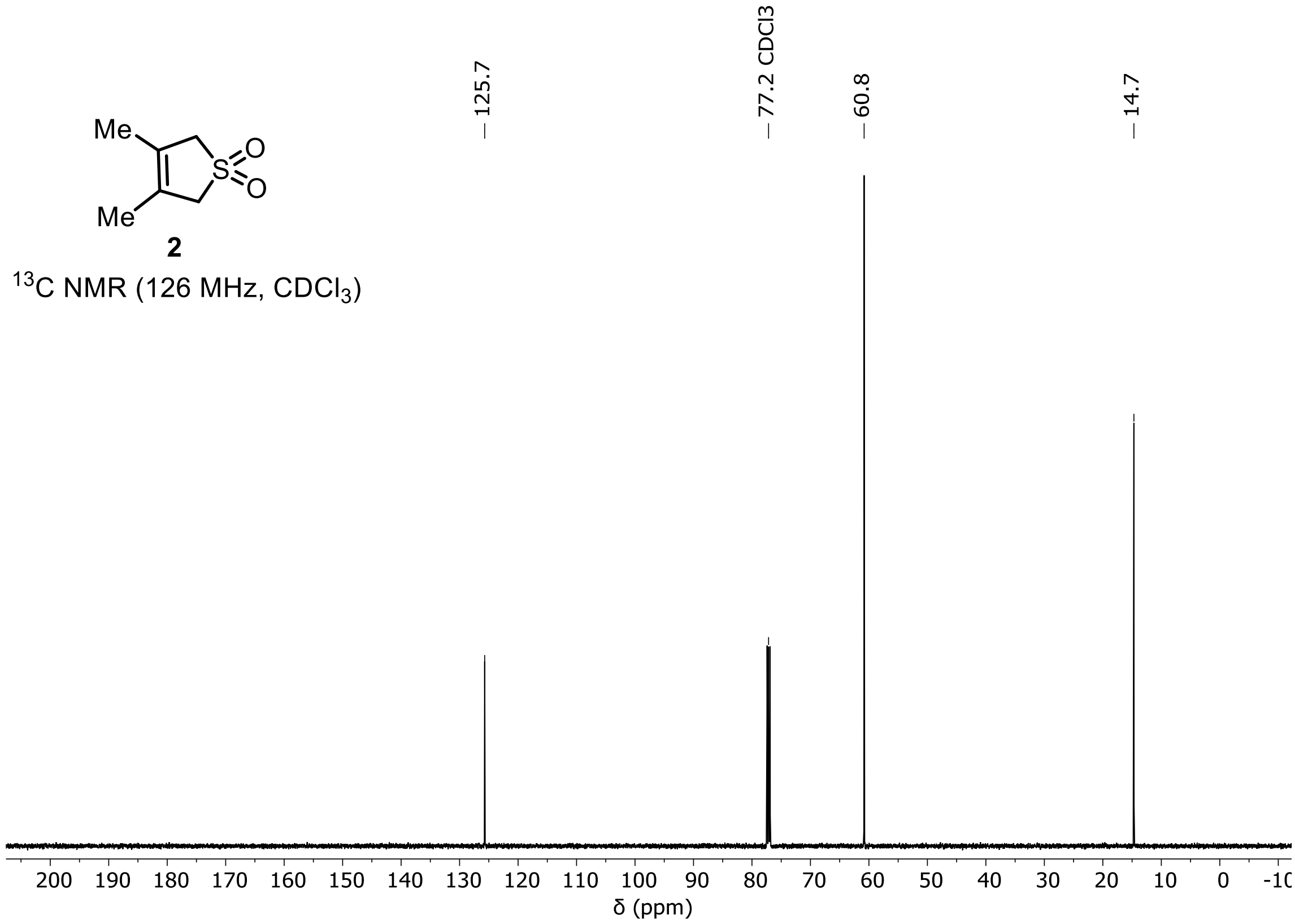
125.7
77.2 CDCl3
60.8
14.7
Me
Me
S
O
O
2
$^{13}$C NMR (126 MHz, $CDCl_3$)
200 190 180 170 160 150 140 130 120 110 100 90 80 70 60 50 40 30 20 10 0 -10
δ (ppm)

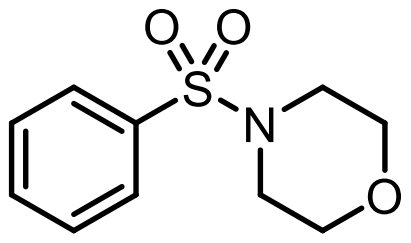

**3**

$^{1}$H NMR (400 MHz, $CDCl_3$)

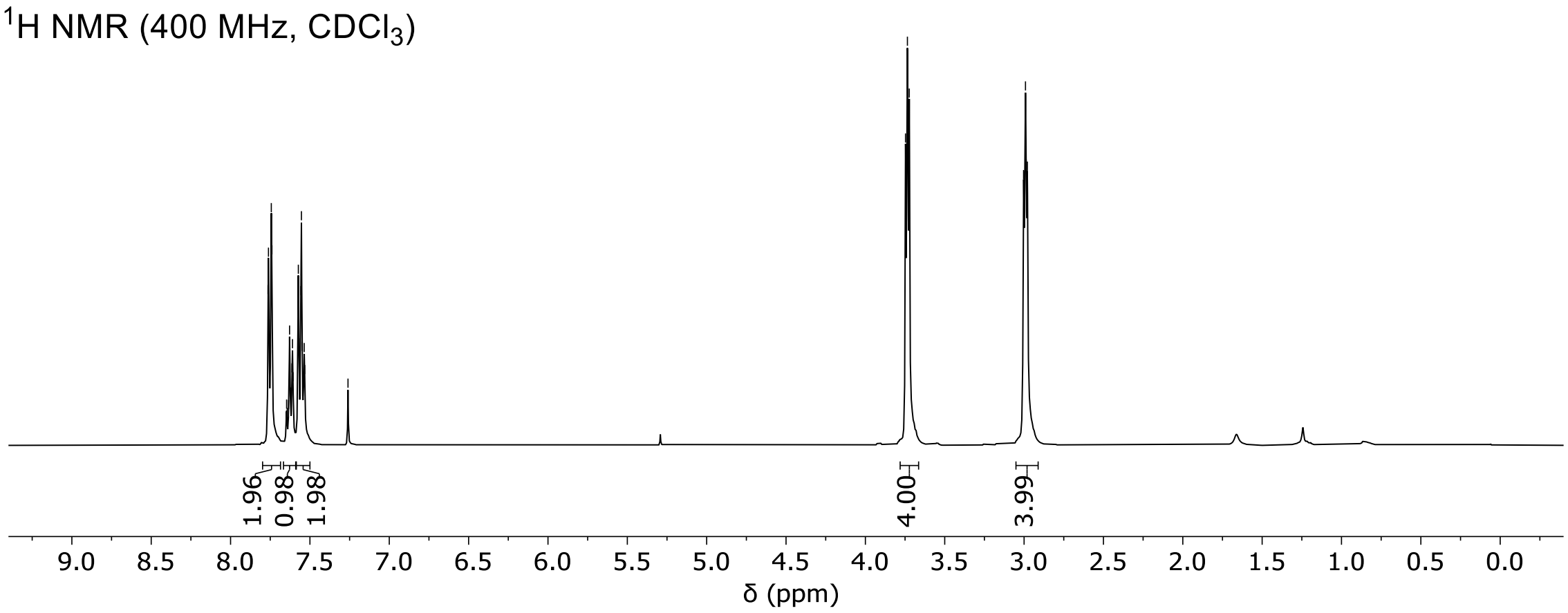


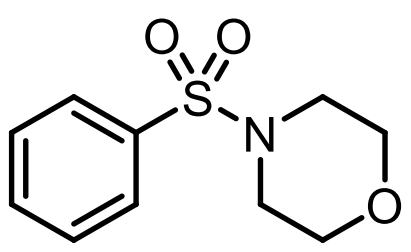

**3**

$^{13}$C NMR (126 MHz, $CDCl_3$)

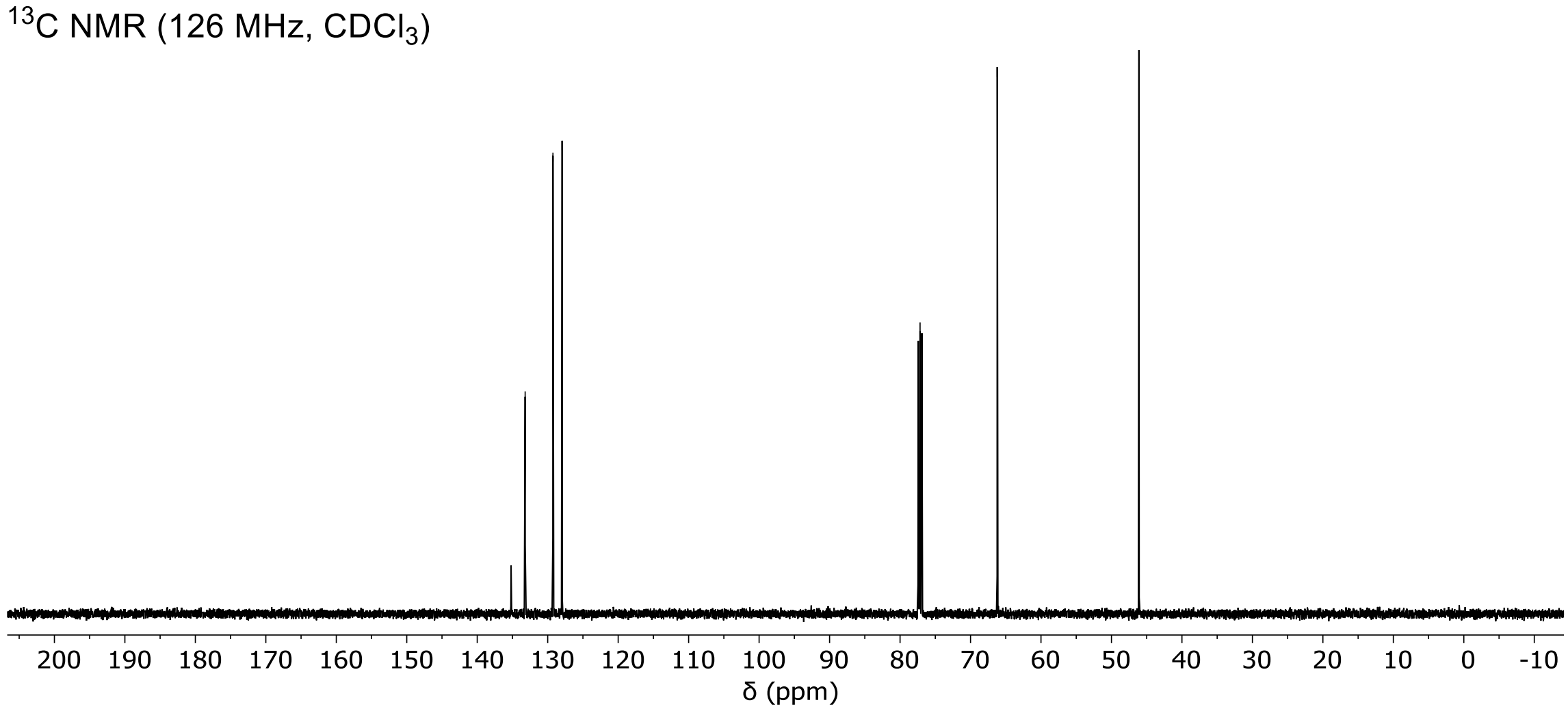

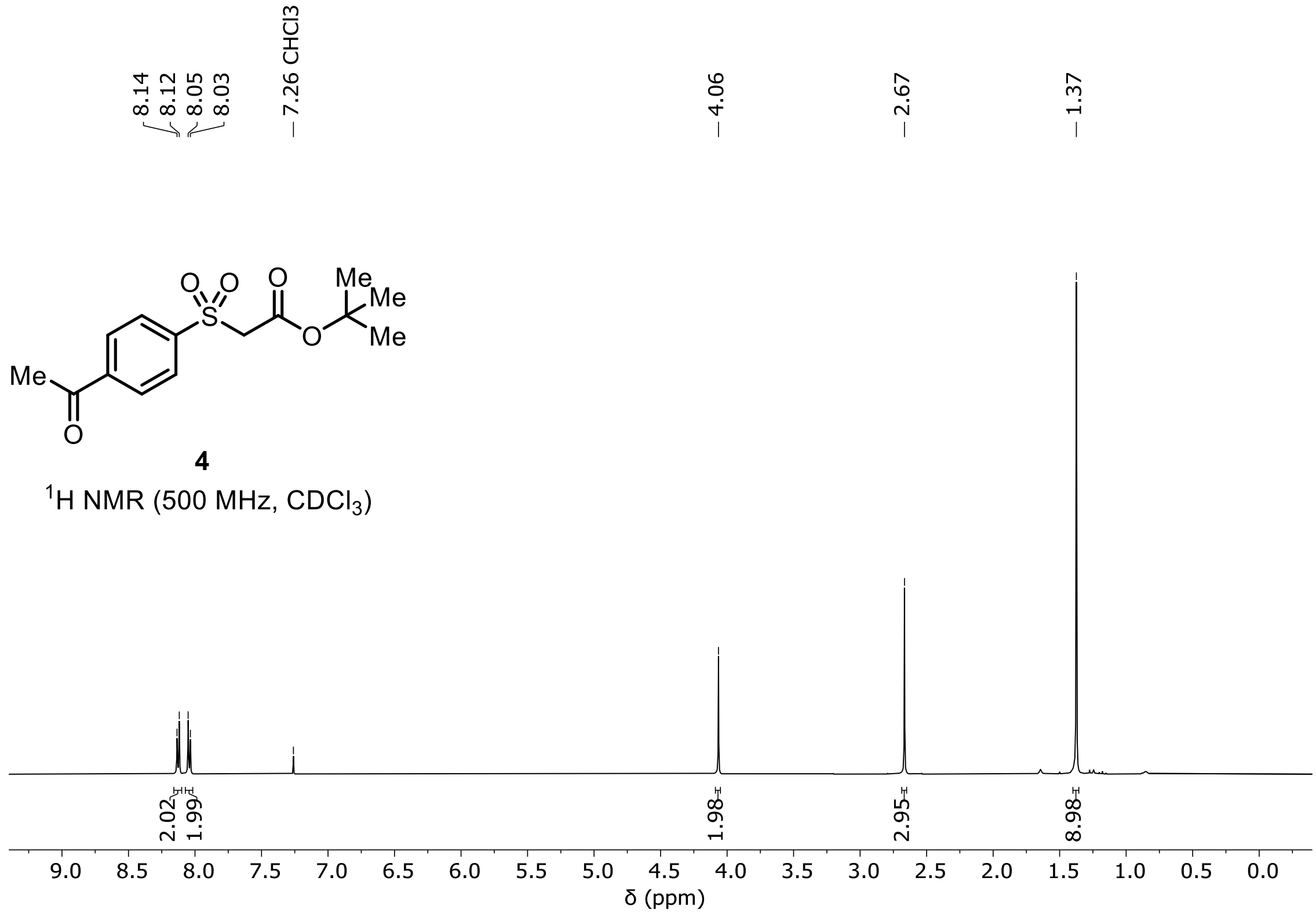
8.14
8.12
8.05
8.03
7.26 CHCl3
4.06
2.67
1.37
Me
O
O
S
O
O
Me
Me
Me
O
4
$^{1}$H NMR (500 MHz, $CDCl_3$)
2.02
1.99
1.98
2.95
8.98
9.0 8.5 8.0 7.5 7.0 6.5 6.0 5.5 5.0 4.5 4.0 3.5 3.0 2.5 2.0 1.5 1.0 0.5 0.0
δ (ppm)

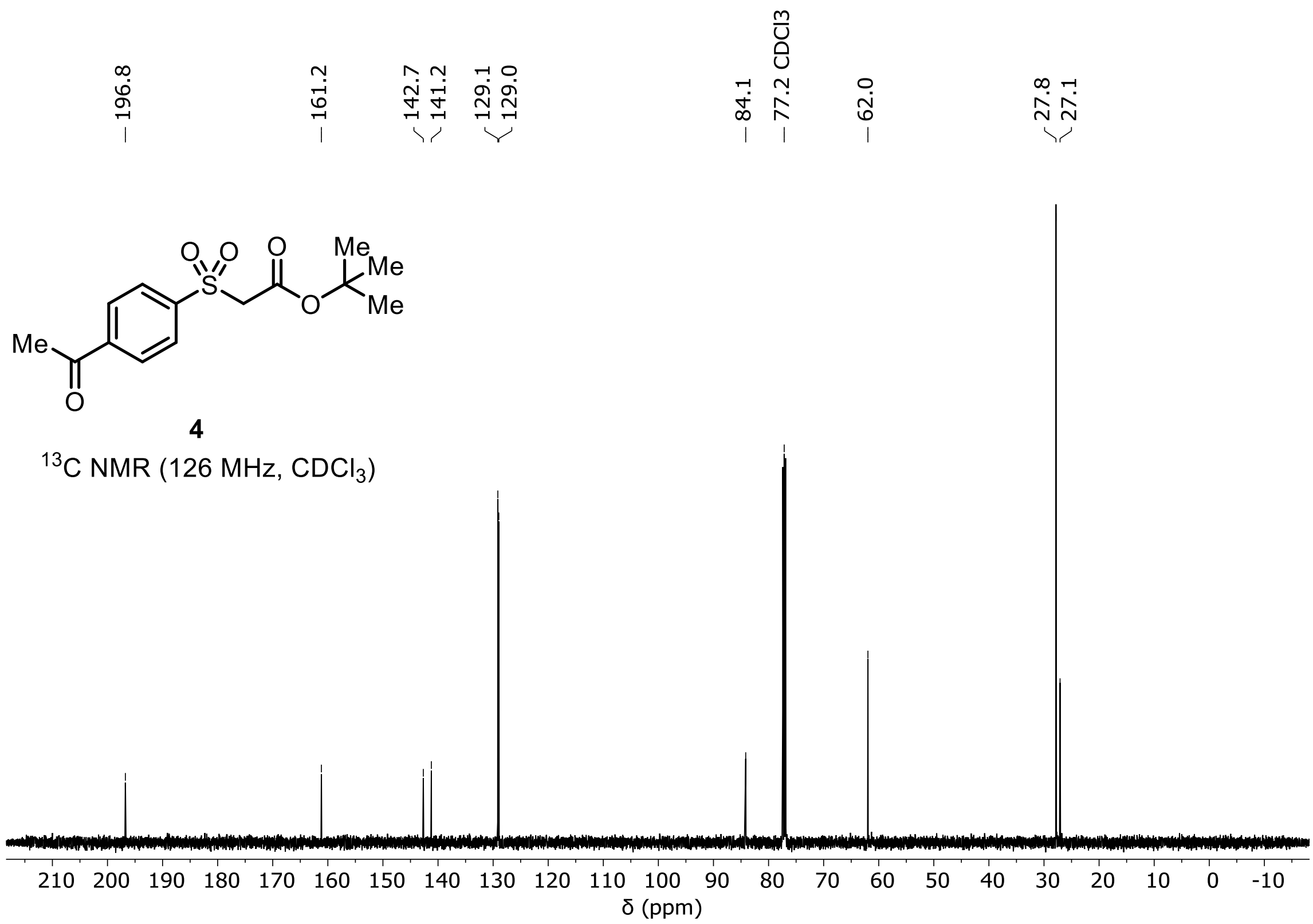
196.8
161.2
142.7
141.2
129.1
129.0
84.1
77.2 CDCl3
62.0
27.8
27.1
Me
O
O
S
O
O
Me
Me
Me
O
4
$^{13}$C NMR (126 MHz, $CDCl_3$)
210 200 190 180 170 160 150 140 130 120 110 100 90 80 70 60 50 40 30 20 10 0 -10
δ (ppm)

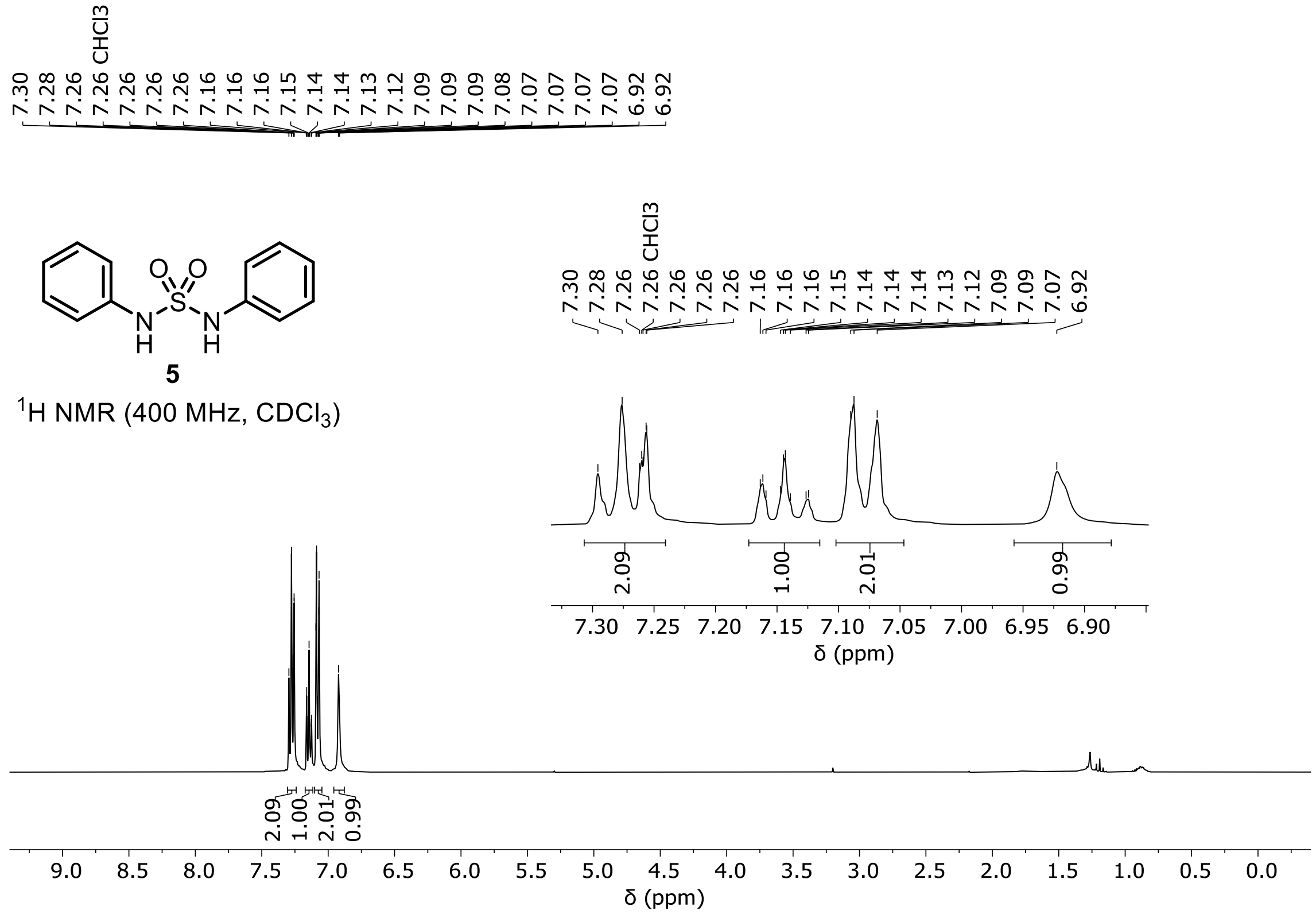
7.30
7.28
7.26
7.26 CHCl3
7.26
7.26
7.26
7.16
7.16
7.16
7.15
7.14
7.14
7.13
7.12
7.09
7.09
7.09
7.08
7.07
7.07
7.07
7.07
6.92
6.92
5
1H NMR (400 MHz, CDCl3)
2.09
1.00
2.01
0.99
δ (ppm)

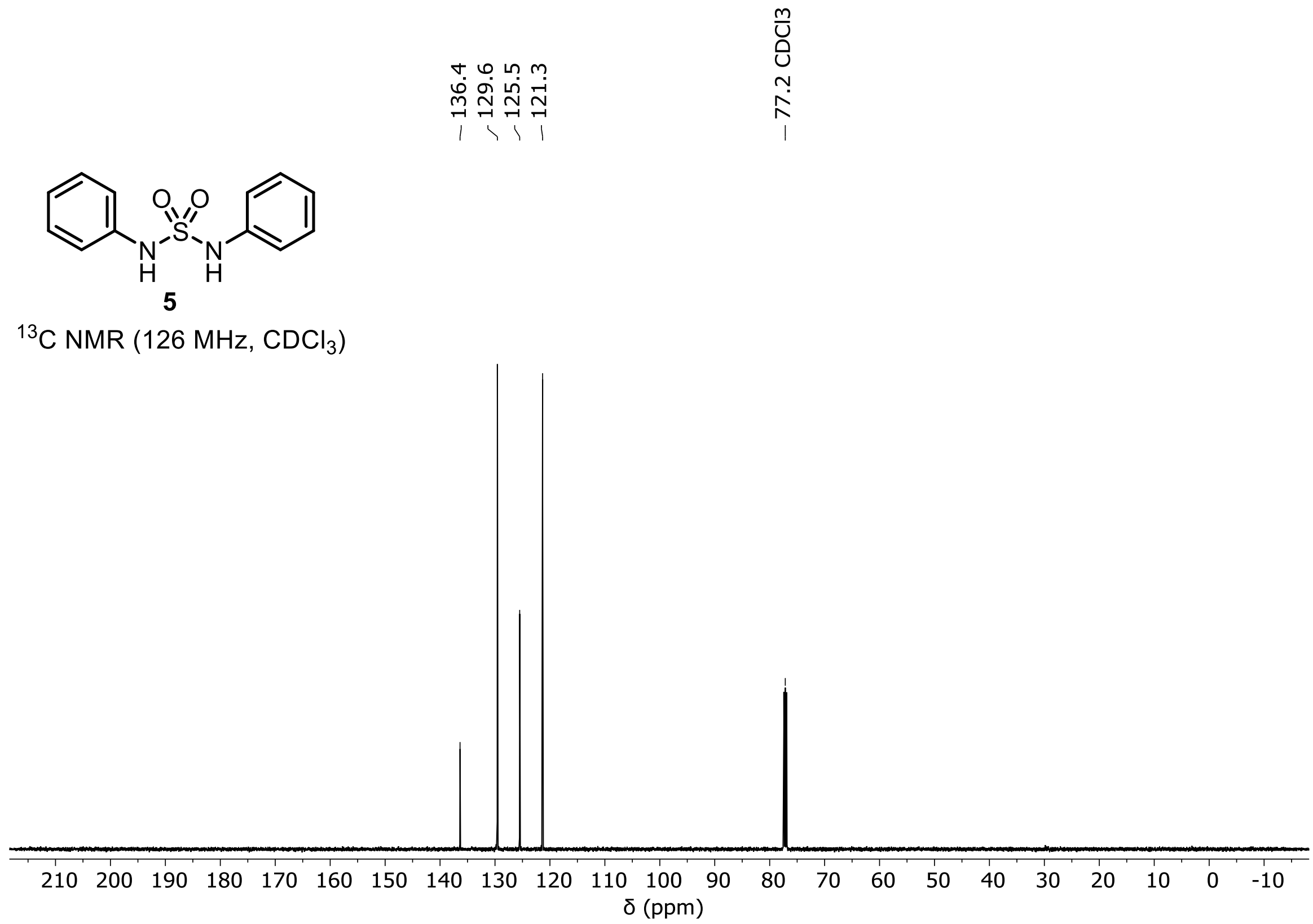
136.4
129.6
125.5
121.3
77.2 CDCl3
5
13C NMR (126 MHz, CDCl3)
δ (ppm)

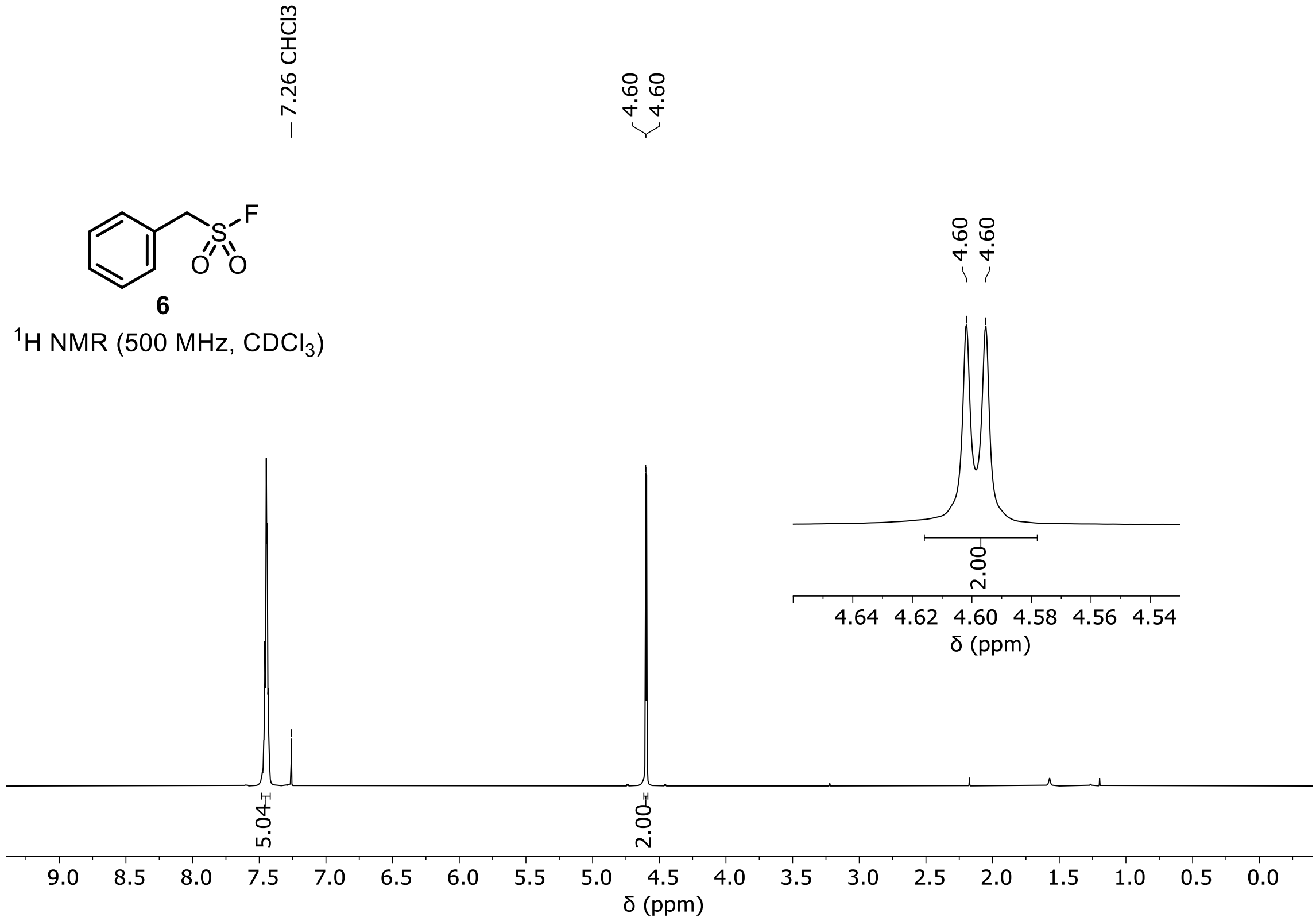


**6**

$^1H$ NMR (500 MHz, $CDCl_3$)

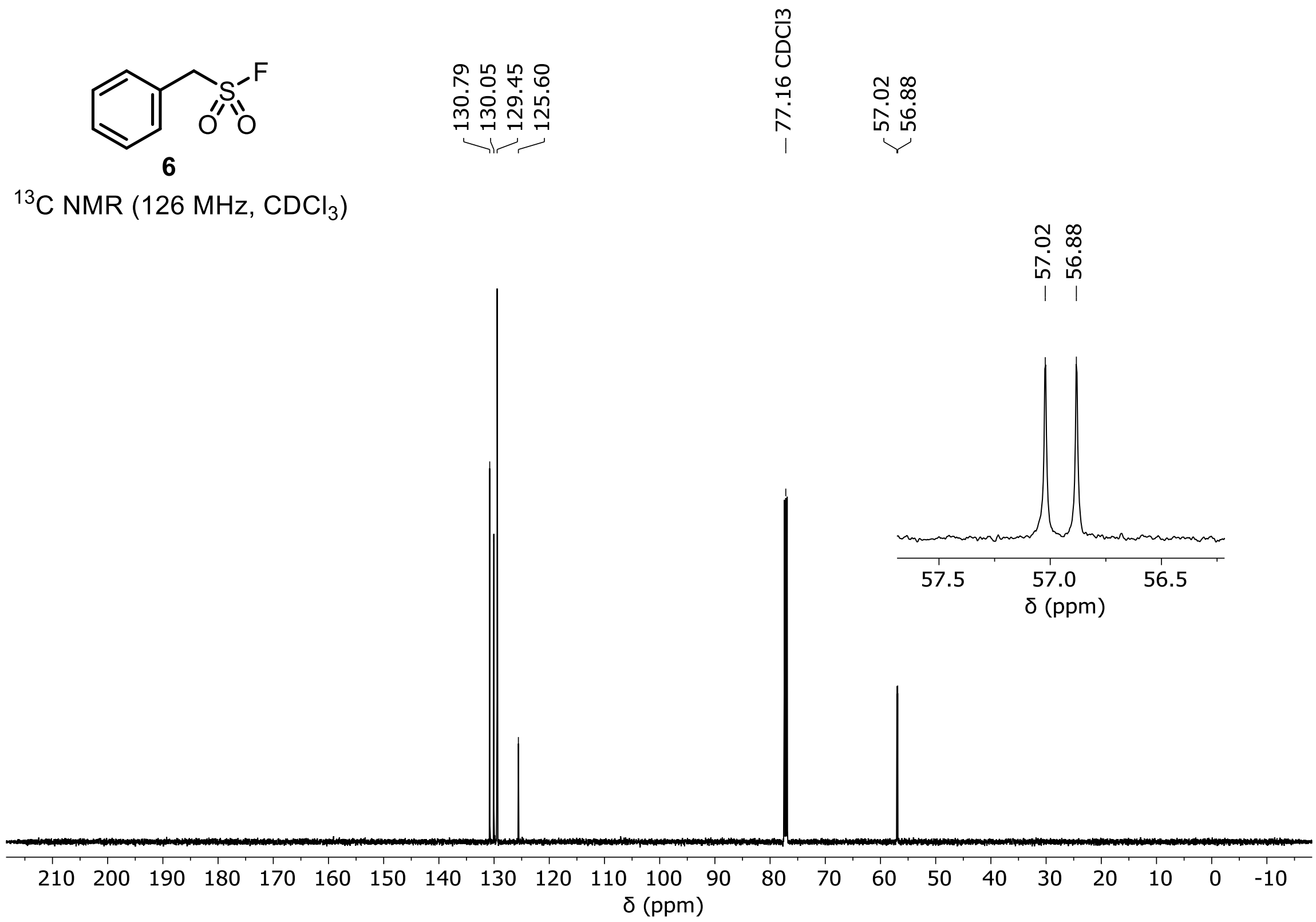


**6**

$^{13}C$ NMR (126 MHz, $CDCl_3$)

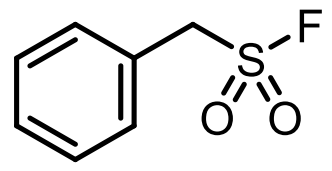

**6**

$^{19}$F NMR (376 MHz, $CDCl_3$)

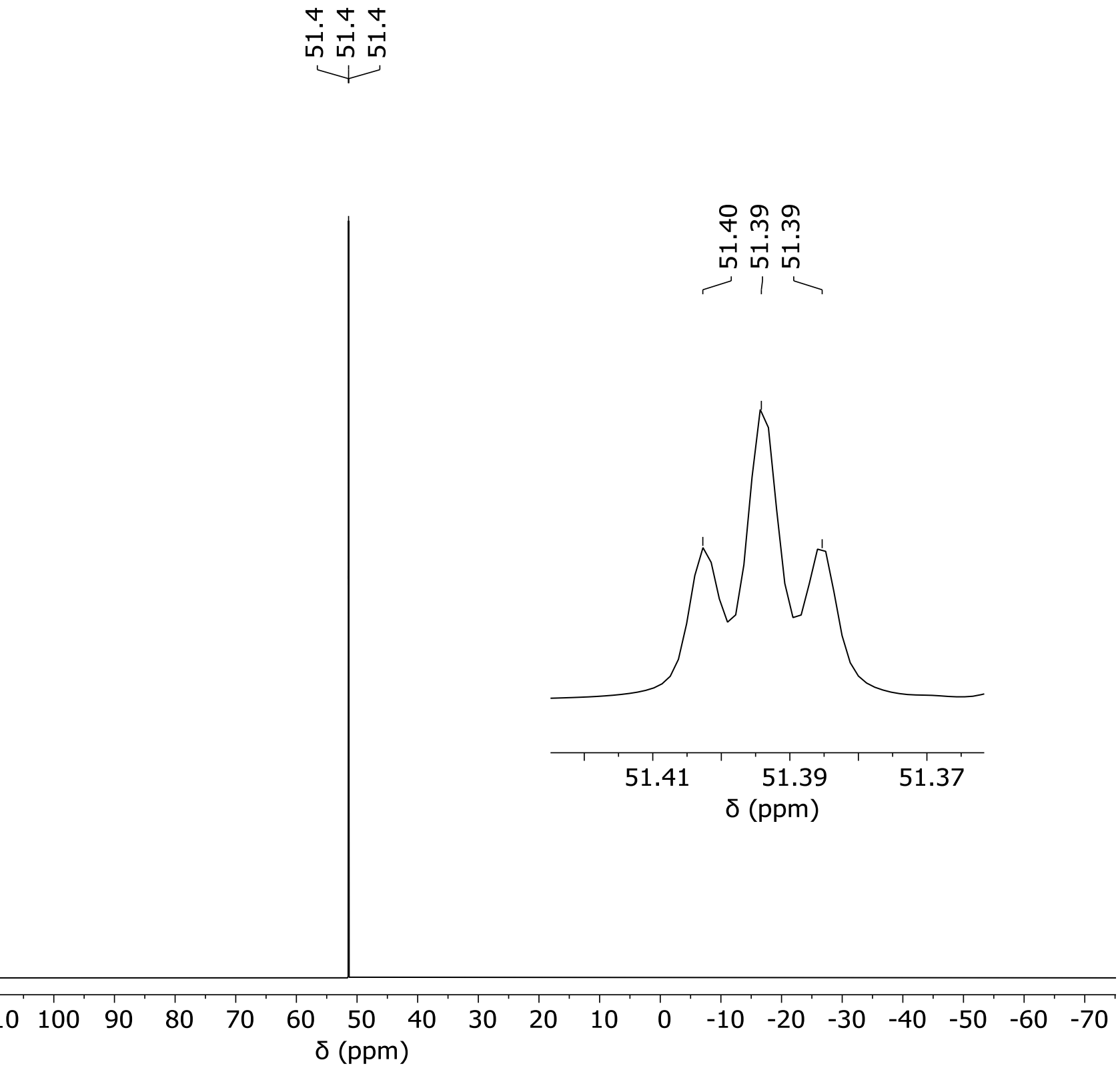

## 5.3 Coordinates of Optimized Structures

**$[ClSO_2]^-$ [Å]**

| | | | |
|---|---|---|---|
| Cl | −5.011230000 | −0.867122000 | −0.433680000 |
| S | −3.906419000 | 1.311473000 | 0.236314000 |
| O | −4.657213000 | 1.694690000 | 1.426378000 |
| O | −2.530819000 | 0.901447000 | 0.493452000 |

**$[Cl(SO_2)_2]^-$ [Å]**

| | | | |
|---|---|---|---|
| S | −4.999302000 | 0.849004000 | 1.269787000 |
| O | −4.912011000 | 0.786863000 | −0.176793000 |
| O | −6.011338000 | 1.741031000 | 1.800300000 |
| Cl | −6.078766000 | −1.548594000 | 1.874005000 |
| S | −6.188433000 | −0.730836000 | 4.445965000 |
| O | −6.621807000 | −1.939277000 | 5.120929000 |
| O | −4.809299000 | −0.345331000 | 4.672247000 |

**$[Cl(SO_2)_3]^-$ [Å]**

| | | | |
|---|---|---|---|
| S | −5.56411401910460 | 1.62662114837994 | 0.48158864586805 |
| O | −4.39560140170404 | 1.26128470639531 | −0.28481514177982 |
| O | −6.83666546509481 | 1.16424438958607 | −0.02513040042809 |
| Cl | −5.22732154543730 | 0.06958667792689 | 2.85586405110292 |
| S | −5.47039559889270 | −1.39610975071917 | 5.28179428815487 |
| O | −6.88129048845857 | −1.27860333587835 | 5.57357515211363 |
| O | −4.99947716996490 | −2.70714572787448 | 4.89766546870690 |
| S | −4.97965366813660 | 2.35462811562682 | 4.37064850755655 |
| O | −4.05084837725087 | 1.89592084386407 | 5.38077449589198 |
| O | −4.45769626595555 | 3.28682993269289 | 3.39427893281299 |

**$[Cl(SO_2)_4]^-$ [Å]**

| | | | |
|---|---|---|---|
| S | −4.666479000 | 1.149490000 | 0.956711000 |
| O | −4.135777000 | −0.158868000 | 0.650439000 |

| | | | |
|---|---|---|---|
| O | −5.465128000 | 1.781719000 | −0.062991000 |
| Cl | −6.602207000 | 0.399532000 | 3.028025000 |
| S | −4.709689000 | −1.241205000 | 4.464983000 |
| O | −5.426201000 | −1.456987000 | 5.698310000 |
| O | −4.604435000 | −2.371381000 | 3.574837000 |
| S | −5.645892000 | 2.657001000 | 4.462753000 |
| O | −4.561287000 | 2.030873000 | 5.181884000 |
| O | −5.275590000 | 3.556744000 | 3.392800000 |
| S | −7.060831000 | −2.022438000 | 1.339256000 |
| O | −7.880090000 | −2.847406000 | 2.195075000 |
| O | −7.714182000 | −1.425050000 | 0.199757000 |

## 5.4 Literature


[1] SAINT. *Bruker AXS Inc.*, Bruker, Madison, Wisconsin, (USA), 2016.

[2] L. Krause, R. Herbst-Irmer, G. M. Sheldrick, D. Stalke, "Comparison of Silver and Molybdenum Microfocus X-Ray Sources for Single-Crystal Structure Determination" *Journal of applied crystallography* **1**, (2015) 48, https://doi.org/10.1107/S1600576714022985.

[3] G. M. Sheldrick, "Crystal Structure Refinement with SHELXL" *Acta Crystallographica Section C* **1**, (2015) 71, https://doi.org/10.1107/S2053229614024218.

[4] G. M. Sheldrick, "SHELXT - Integrated Space-Group and Crystal-Structure Determination" *Acta crystallographica. Section A, Foundations and advances* **1**, (2015) 71, https://doi.org/10.1107/S2053273314026370.

[5] C. R. Groom, I. J. Bruno, M. P. Lightfoot, S. C. Ward, "The Cambridge Structural Database" *Acta crystallographica Section B, Structural science, crystal engineering and materials* **2**, (2016) 72, https://doi.org/10.1107/S2052520616003954.

[6] D. Kratzert, "FinalCif, V149", can be found under https://dkratzert.de/finalcif.html, 2026, (accessed: 03.02.2026).

[7] M. D. Hanwell, D. E. Curtis, D. C. Lonie, T. Vandermeersch, E. Zurek, G. R. Hutchison, "Avogadro: An Advanced Semantic Chemical Editor, Visualization, and Analysis Platform" *Journal of cheminformatics* **1**, (2012) 4, https://doi.org/10.1186/1758-2946-4-17.

[8] L. Bennett, B. Melchers, B. Proppe, Curta: A General-Purpose High-Performance Computer at ZEDAT, Freie Universität Berlin, Freie Universität Berlin, 2020.

[9] C. Lee, W. Yang, R. G. Parr, "Development of the Colle-Salvetti Correlation-Energy Formula into a Functional of the Electron Density" *Physical Review B* **2**, (1988) 37, https://doi.org/10.1103/physrevb.37.785.

[10] A. D. Becke, "Density-Functional Exchange-Energy Approximation with Correct Asymptotic Behavior" *Physical Review A* **6**, (1988) 38, https://doi.org/10.1103/physreva.38.3098.

[11] F. Neese, "The ORCA Program System" *WIREs Computational Molecular Science* **1**, (2012) 2, https://doi.org/10.1002/wcms.81.

[12] E. Caldeweyher, S. Ehlert, A. Hansen, H. Neugebauer, S. Spicher, C. Bannwarth, S. Grimme, "A Generally Applicable Atomic-Charge Dependent London Dispersion Correction" *The Journal of chemical physics* **15**, (2019) 150, https://doi.org/10.1063/1.5090222.

[13] M. J. Frisch, G. W. Trucks, H. B. Schlegel, G. E. Scuseria, M. A. Robb, J. R. Cheeseman, G. Scalmani, V. Barone, G. A. Petersson, H. Nakatsuji, X. Li, M. Caricato, A. V. Marenich, J. Bloino, B. G. Janesko, R. Gomperts, B. Mennucci, H. P. Hratchian, J. V. Ortiz, A. F. Izmaylov, J. L. Sonnenberg, D. Williams-Young, F. Ding, F. Lipparini, F. Egidi, J. Goings, B. Peng, A. Petrone, T. Henderson, D. Ranasinghe, V. G. Zakrzewski, J. Gao, N. Rega, G. Zheng, W. Liang, M. Hada, M. Ehara, K. Toyota, R. Fukuda, J. Hasegawa, M. Ishida, T. Nakajima, Y. Honda, O. Kitao, H. Nakai, T. Vreven, K. Throssell, J. A. Montgomery, Jr., J. E. Peralta, F. Ogliaro, M. J. Bearpark, J. J. Heyd, E. N. Brothers, K. N. Kudin, V. N. Staroverov, T. A. Keith, R. Kobayashi, J. Normand, K. Raghavachari, A. P. Rendell, J. C. Burant, S. S. Iyengar, J. Tomasi, M. Cossi, J. M. Millam, M. Klene, C. Adamo, R. Cammi, J. W. Ochterski, R. L. Martin, K. Morokuma, O. Farkas, J. B. Foresman, and D. J. Fox, Gaussian 16, Gaussian, Inc., Wallingford CT, (USA), 2016.

[14] W. Humphrey, A. Dalke, K. Schulten, "VMD: Visual Molecular Dynamics" *Journal of molecular graphics* **1**, (1996) 14, https://doi.org/10.1016/0263-7855(96)00018-5.

[15] E. D. Glendening, J. K. Badenhoop, A. E. Reed, J. E. Carpenter, J. A. Bohmann, C. M. Morales, P. Karafiloglou, C. R. Landis, F. Weinhold, NBO 7.0, Theoretical Chemistry Institute, University of Wisconsin, Madison, WI, (USA), 2018.

[16] G. A. Zhurko, Chemcraft - Graphical Program for Visualization of Quantum Chemistry Computations. https://www.chemcraftprog.com, Ivanovo (Russia), 2005.

[17] T. Lu, F. Chen, "Multiwfn: A Multifunctional Wavefunction Analyzer" *Journal of Computational Chemistry* **5**, (2012) 33, https://doi.org/10.1002/jcc.22885.

[18] H. Putz, K. Brandenburg, DIAMOND 4.6.4, Crystal Impact GbR, Bonn, (Germany), 2020.

[19] "abcr", can be found under https://abcr.com/de_de/produkte, 2025, (accessed: 02.12.2025).

[20] "Linde", can be found under https://www.linde-gas.de/shop/de/de-ig/home, 2026, (accessed: 02.02.2026).

[21] "Sigmaaldrich", can be found under https://www.sigmaaldrich.com/DE/de, 2025, (accessed: 02.12.2025).

[22] J. C. Poutsma, O. E. Schroeder, R. R. Squires, "Experimental Chloride Ion Affinities and Theoretical Predictions for the Absolute Heats of Formation of $SiCl_2$ and SiClF" *Chemical Physics Letters* **4-6**, (2004) 389, https://doi.org/10.1016/j.cplett.2004.03.137.

[23] H. Böhrer, N. Trapp, D. Himmel, M. Schleep, I. Krossing, "From Unsuccessful $H_2$-Activation with FLPs Containing $B(Ohfip)_3$ to a Systematic Evaluation of the Lewis Acidity of 33 Lewis Acids Based on Fluoride, Chloride, Hydride and Methyl Ion Affinities" *Dalton Transactions* **16**, (2015) 44, https://doi.org/10.1039/c4dt02822h.

[24] P. Erdmann, L. Greb, "Multidimensional Lewis Acidity: A Consistent Data Set of Chloride, Hydride, Methide, Water and Ammonia Affinities for 183 p-Block Element Lewis Acids" *ChemPhysChem* **10**, (2021) 22, https://doi.org/10.1002/cphc.202100150.

[25] P. Erdmann, J. Leitner, J. Schwarz, L. Greb, "An Extensive Set of Accurate Fluoride Ion Affinities for p-Block Element Lewis Acids and Basic Design Principles for Strong Fluoride Ion Acceptors" *ChemPhysChem* **10**, (2020) 21, https://doi.org/10.1002/cphc.202000244.

[26] S. G. Lias, J. E. Bartmess, J. F. Liebman, J. L. Holmes, R. D. Levin, W. G. Mallard, "Gas-Phase Ion and Neutral Thermochemistry" *Journal of Physical and Chemical Reference Data*, (1988) 17.

[27] H. Woolven, C. González-Rodríguez, I. Marco, A. L. Thompson, M. C. Willis, "DABCO-Bis(sulfur dioxide), DABSO, as a Convenient Source of Sulfur Dioxide for Organic Synthesis: Utility in Sulfonamide and Sulfamide Preparation" *Organic letters* **18**, (2011) 13, https://doi.org/10.1021/ol201957n.

[28] E. J. Emmett, B. R. Hayter, M. C. Willis, "Palladium-Catalyzed Synthesis of Ammonium Sulfinates from Aryl Halides and a Sulfur Dioxide Surrogate: a Gas- and Reductant-Free Process" *Angewandte Chemie International Edition* **38**, (2014) 53, https://doi.org/10.1002/anie.201404527.

[29] A. T. Davies, J. M. Curto, S. W. Bagley, M. C. Willis, "One-Pot Palladium-Catalyzed Synthesis of Sulfonyl Fluorides from Aryl Bromides" *Chemical Science* **2**, (2017) 8, https://doi.org/10.1039/c6sc03924c.
